# Artificial intelligence driven microalgae based green fabrication and bioenergy systems for sustainable energy materials and biowaste valorization

**DOI:** 10.3389/fchem.2026.1858141

**Published:** 2026-06-24

**Authors:** Mohaddeseh Abbaszadeh, Sai Kumar Punna, Suvarshitha Pusuluru, Melvin S. Samuel, Rahul Sampat Khandge, Selvarajan Ethiraj, Hanadi A. Almukhlifi, Farid Menaa

**Affiliations:** 1 W Booth School of Engineering Practice and Technology, McMaster University, Hamilton, ON, Canada; 2 Department of Materials Science and Engineering, University of Houston, Houston, TX, United States; 3 Department of Engineering Data Science, University of Houston, Houston, TX, United States; 4 Department of Materials Science and Engineering, University of Wisconsin Milwaukee, Milwaukee, WI, United States; 5 Department of Bioengineering, Saveetha School of Engineering, Saveetha Institute of Medical and Technical, Chennai, India; 6 Department of Biochemistry, Chemistry and Physics, Centre for Advanced Materials Science, Georgia Southern University, Statesboro, GA, United States; 7 Department of Genetic Engineering, College of Engineering and Technology, School of Bioengineering, SRM Institute of Science and Technology, Kattankulathur, Tamil Nadu, India; 8 Department of Chemistry, Faculty of Science, University of Tabuk, Tabuk, Saudi Arabia; 9 Department of Biomedical and Environmental Engineering (BEE) California Innovations Corporation (CIC), San Diego, CA, United States

**Keywords:** artificial intelligence (AI), biowaste valorization, energy storage materials, green fabrication, machine learning (ML), microalgae bioenergy

## Abstract

In recent years, the bioenergy domain has experienced substantial advancement, largely driven by the integration of advanced technologies such as artificial intelligence (AI) and machine learning (ML), particularly in optimizing microalgae-based systems for biofuel production and sustainable biowaste conversion. AI techniques, including support vector machines (SVM) and artificial neural networks (ANN), have demonstrated strong capabilities in modelling complex nonlinear relationships, enabling improved prediction of process parameters and enhanced system performance. In microalgal bioenergy systems, ANN-based models have achieved high predictive accuracy, with coefficients of determination exceeding 0.93, facilitating efficient biomass production, pollutant removal, and resource optimization. Beyond biofuel generation, microalgal biomass represents a promising renewable feedstock for the green fabrication of advanced energy materials, including carbon-based nanostructures and bio-derived electrodes applicable in energy storage systems such as batteries and supercapacitors. Techniques such as genetic algorithms and ANN-based control systems enable real-time optimization of photobioreactor operations, improving energy recovery efficiency and reducing operational costs. Furthermore, AI-assisted catalytic and thermochemical process have contributed to higher conversion efficiencies and improved sustainability outcomes. The integration of AI with microalgae-based bioenergy and material fabrication systems supports circular economy principles by enabling the conversion of biowaste into high value energy products and functional materials. Despite these advancements, challenges such as computational complexity, data availability, and feedstock variability remain. Addressing these issues through interdisciplinary research is essential for scaling AI-enabled bioenergy platforms. Overall, this study highlights the transformative potential of AI in advancing sustainable bioenergy systems and eco-friendly material fabrication, contributing to global decarbonization and zero-waste goals.

## Highlights

- AI enhanced microalgae-based biofuel production and biowaste valorization
- Machine learning enables green fabrication of carbon-based energy materials
- Microalgal biomass serves as a sustainable precursor for energy storage applications
- AI driven optimization improves catalytic, fermentation, and photobioreactor processes.
- Integrated bioenergy-materials systems support circular economy and decarbonization goals.

## 1 Introduction

Global energy demand is projected to increase substantially in the coming decades, driven by industrialization, rapid urban expansion, and sustained economic development (Long et al., 2022). This escalating requirement has intensified reliance of fossil-based fuel including coal, crude oil, and natural gas, which collectively contribute approximately 86% of total global energy consumption (Shahbeik et al., 2022). This extensive utilization of these finite resources has generated significant environmental and socio-economic concerns, including greenhouse gas emissions, climate change, and geopolitical and economic vulnerabilities (Shafizadeh et al., 2023). Data from the International Energy Agency (IEA) indicate that the world-wide energy consumption rose by 26% between 2000 and 2010, following a two increase from 1970 to 2000. By 2017, total global energy use reached approximately 13.5 billion tonnes of oil equivalent (^%^ EJ), with forecast predicting a further 28% increase by 2040, approaching nearly 739 quadrillion British Thermal units (Btu) (Elgarahy et al., 2021). Although fossil fuels continue to dominate the global energy mix, concerns regarding resource depletion and environmental sustainability emphasize the necessity for alternative renewable energy pathways (Li et al., 2021b). In response to challenges associated with fossil fuel dependence and ecological degradation, biofuels and bioenergy have gained recognition as promising renewable alternative. These energy carriers are derived from biomass and organic substrates, including plant matter, agricultural residues, and microbial fermentation processes (Elgarahy et al., 2023a; b). Biomass is widely regarded as a carbon-neutral resource capable of substituting petroleum-derived fuels, thereby contributing to greenhouse gas mitigation and climate change abatement (Li et al., 2023; Savi et al., 2017). In contrast to fossil fuels, biomass-based bioenergy operates within a closed carbon cycle, as carbon dioxide releases during combustion is reabsorbed through photosynthetic processes, enhancing its sustainable profile. Biomass composition varies depending on source and in generally categorized into lignocellulosic and non-lignocellulosic fractions. Lignocellulosic biomass (LCB), encompassing grasses, woody material, and agricultural residues, is particularly advantageous for biofuel production due to its abundant carbohydrate content and widespread availability (Rozzi et al., 2020). Projections suggest that by 2050, biomass could supply nearly two-third of the world’s direct renewable energy demand, underscoring its strategic importance in future energy systems (Ab Rasid et al., 2021). The conversion of LCB into biofuels present a viable pathway for substantial reductions in CO_2_ emission (Mishra et al., 2022). Current global estimates indicate an annual production of approximately 1.3 × 10^10^ metric tons of lignocellulosic biomass. China produces around 126 million metric tonnes of wheat straw, while the United States contributes 80% of agricultural residues and 25% of global sugarcane bagasse output. Europe accounts for approximately 64% of worldwide oat straw production (Velvizhi et al., 2022). Lignocellulosic biomass primarily comprises cellulose, hemicellulose, and lignin, with lignin conferring structural rigidity to plant matrices (Lorenzo et al., 2017). Biofuel production from biomass primarily involves sequential stages such as pretreatment, enzymatic saccharification, fermentation, and thermochemical conversion. Advances in biomass valorisation have improved process efficiency and economic feasibility through the incorporation of high-efficiency catalyst, optimized enzymatic hydrolysis, and innovative bioconversion strategies (Eloffy et al., 2022). These progressions reinforce the viability of biomass-derived energy in facilitating a sustainable, low-carbon transition. Biomass can be transformed into value-added biofuels through several principal pathways, including:

- Biochemical processing: processes such as anaerobic digestion and microbial fermentation enhance the production of bioethanol and biogas.
- Thermochemical pathways: technologies including pyrolysis, torrefaction, gasification, and combustion enable effective energy recovery from biomass.
- Hydrothermal upgrading: high-temperature, water-mediated processes convert biomass into bio-oil and biochar (Ma et al., 2023).

The International Energy Agency (IEA) has outlines strategic targets aimed at achieving net-zero emission by 2050, emphasizing the deployment of advanced biofuels such as hydrotreated vegetable oil, bio-synthetic natural gas, dimethyl ether, and biomass-to-liquid fuels (IEA, 2020). Ongoing innovations in bioprocess engineering, integrated with AI-based optimization tools, are enhancing the efficiency, scalability, and environmental performance of biofuel systems. Machine learning techniques are increasingly applied to refine fermentation parameters, optimize enzymatic activities, and improve feedstock conversion efficiency, thereby increasing yield and economic competitiveness (Lin and Lu, 2021). The transition toward a circular economy-prioritizing waste-to-energy conversion, resource recovery, and value-added waste transformation-is essential for meeting sustainability objectives (MacLellan et al., 2017). This paradigm minimizes waste generation while maximizing resource utilization, thereby reducing the environmental footprint of sectors such as palm oil production, which has been associated with deforestation, greenhouse gas (GHG) emissions, and biodiversity decline (Maeda et al., 2023). These initiatives align with the United Nations Sustainable Development Goals (SDGs), particularly SDG 12 (Responsible Consumption and Production) and SDG 13 (Climate Action), which promote sustainable industrial operations, emission reduction, and environmental innovation. The palm oil industry holds significant potential to catalyse sustainability transitions, with SDG 12 advocating responsible production practices and SDG 13 emphasizing emission mitigation strategies. Circular bioeconomy frameworks enable the conversion of industrial residue into bioenergy, organic fertilizers, and bio-based products, strengthening sustainability throughout the value chain. Embedding these principles with palm oil production ensures that by-products are valorised rather than discarded, thereby reducing land degradation and pollution. The global palm oil market, valued at approximately USD 70.44 billion in 2023, is projected to expand at a compound annual growth rate (CAGR) of 5.1%, reaching nearly USD 100.04 billion by 2030 (Mahoto et al., 2023; Myint et al., 2019). As the leading global producer and exporter, Indonesia exerts substantial influence over sustainability trajectories. Technological innovations-including bioenergy integration, carbon-neutral certification schemes, and regenerative agricultural practices-contribute to GHG mitigation and reinforcement commitments to sustainable development and energy security (Mancini et al., 2020).

Adopting circular bioeconomy principles within the palm oil sector offers pathways for meaningful GHG reductions through the conversion of organic residues into renewable energy and bio-based commodities, thereby decreasing fossil fuel dependence while fostering sustainable economic growth (Matheri, A.N et al., 2018). The Asia-Pacific region, supported by demographic growth and industrialization, is anticipated to accelerate circular bioeconomy adoption, while north America presents strong potential for scaling sustainable palm oil strategies (Jusoh et al., 2021). Despite these prospects, transitioning towards a circular and sustainable palm oil bioeconomy entails considerable challenges, necessitating innovative technologies and stringent waste governance. The sector has historically been linked to deforestation and carbon emissions, particularly in Indonesia and Malaysia, where land use changes has caused extensive forest depletion. The carbon footprint of crude palm oil production ranges from 637 to 1,131 kg CO_2_ eq per ton, underscoring the urgency of strengthened sustainability interventions (Mulabagal et al., 2022). Effective transition requires sustainable land-use management, investment in circular technologies, and robust environmental regulation. The circular bioeconomy constitutes a transformative model centred on waste minization and optimal resource utilization. In contrast to the linear “take-make-dispose” paradigm, circular systems retain materials and products in continuous use, extracting maximal value prior to recovery and regeneration. By designing out waste and closing resource loops, this framework enhances sustainability, economic stability, and environmental protection (Miikkulainen and Forrest, 2021).

- Key principles of circular bioeconomy: Preservation of Natural Capital: sustainable resource stewardship minimizes waste, regulates finite material use, and ensures renewable extraction rates remain within ecological regeneration limits.
- Resource optimization: materials and products circulate within the economy at their highest utility through reuse, recycling, and repurposing.
- System Effectiveness: adverse externalities are mitigated through sustainable material and system design, improving industrial efficiency and reducing environmental burdens (Mirkouei, A., 2019).

The circular bioeconomy is critically important as it reduces reliance on fine resources, curbs environmental degradation, and strengthens economic performance. Tambovceva and Titko (2020) emphasize that circular strategies preserve the value and integrity of materials within closed-loop systems powered by renewable energy. Estimates suggest that by 2030, circular models could yield up to USD 2.2 trillion in annual net economic benefits, substantially lowering costs in sectors such as food, transportation, and housing (Hysa et al., 2020). Moreover, circular bioeconomy frameworks enhance resource productivity, generate employment, and stimulate innovation, fostering resilient and sustainable economic systems. Integration of these principals into industrial operations enables effective waste-to-resource conversions, transforming agricultural and industrial residues into bioenergy, bioplastic, and other bio-based commodities (Mowbray et al., 2021). Over the past decade, their role in decarbonizing high-emission industries such as palm oil production has gained increasing attention. Early investigations demonstrated the efficacy of wate-to-energy systems and resource recovery in reducing environmental impacts (Kerdlap et al., 2019; Fernando et al., 2022). Subsequent technological progress has introduced impactful innovations such as carbon capture and utilization (CCU) and biogas recovery systems, enhancing operational feasibility and decarbonization efficiency (Gowd et al., 2023; Makepa and Chihobo, 2024).

**FIGURE 1:**
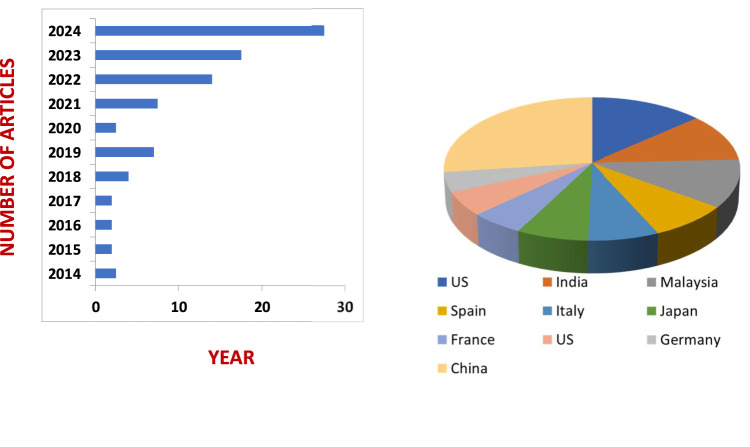
Bar chart on the left shows the number of articles published by year from 2014 to 2024, with 2024 having the highest count, nearly thirty articles, and earlier years having fewer. Pie chart on the right displays proportions of articles by country, with sections for US, Spain, France, China, India, Italy, Malaysia, Japan, Germany, each represented by a different color segment.

Daneshvar et al. (2022) reported that carbon capture and utilization (CCU) technologies could reduce CO_2_ emission by upto to 65%, offering scalable mitigation pathways for sustainable palm oil production. Advances in biorefinery integration and net-generation-waste-to-energy platforms have further strengthened resource recovery and emission reduction performance in alignment with global climate objective (Ahmed et al., 2023). Nevertheless, broader implementation remained constrained by economic and regulatory barriers. Market analyses highlight the importance pf effective policy frameworks and financial incentives to accelerate sustainable transitions (Nega et al., 2024). Furthermore, despite progress in decarbonization-particularly in small or technologies, scalability across diverse operational contexts-particularly in small or technologically limited palm oil mills-remain insufficiently understood (Mutlu and Yucel, 2018). To advance widespread adoption of circular bioeconomy innovations, further research must address technical, economic, and infrastructural limitations. Priority areas for future investigation include enhancing carbon capture and utilization (CCU) performance in industrial-scale applications. Designing economically viable biorefinery and bioenergy systems to support scalable deployment. Reinforcing regulatory structures and incentive mechanism to facilitate accelerated implementation (Muzyka, R et al., 2023). As industries increasingly prioritise sustainability, embedding circular bioeconomy principles become fundamental to establishing low-carbon, resource-efficient production frameworks. Through the deployment of advanced waste management practices, carbon-neutral technologies, and sustainable supply chain configurations, the palm oil sector-alongside broader industrial systems-can progress toward a climate resilient and economically sustainable trajectory (Naraki et al., 2022). The incorporation of machine learning (ML), methodologies into bioenergy systems has expanded considerably, facilitating improvements in biomass conversion efficiency, fermentation performance, and overall biofuel process regulation (Nastro et al., 2022). Despite these technological gains, significant barriers persist, necessitating interdisciplinary cooperation to fully harness ML’s capabilities within the bioenergy domain. A major constrain involves the limited methodological expertism among bioenergy researchers regarding appropriate ML implementation. Inadequate application can lead to complications such as data leakage, model overfitting, and unreliable predictive outcomes, thereby undermining the credibility of ML-driven insights (Zhang et al., 2020). Addressing this concern requires the development of standardized ML protocols and strengthened training model validation procedures. Furthermore, sophisticated ML architectures such as deep neural networks (DNNs) pose interpretability challenges, as their “black box” structure complicates assessment of the scientific validity of generated outputs (Zhang et al., 2019). Enhancing transparency in ML-based bioenergy research necessitates the integration of explainable artificial intelligence (XAI) methodologies (Wang et al., 2022). Equally important is the embedding of domain-specific knowledge into ML frameworks to ensure that bioenergy principles inform predictive modelling. Hybrid modelling strategies, wherein ML augments conventional bioenergy models, can deliver more theoretically consistent and robust solutions (Daneshvar et al., 2022). Additionally, the transferability and scalability of ML tools across diverse bioenergy segments-including biomass pretreatment, enzymatic hydrolysis optimization, and biohydrogen production-remain insufficiently examined, restricting broader industrial implementation (Ahmed et al., 2023). Considering the substantial datasets generated through industrial bioenergy production and monitoring systems, ML offers strong potential for pattern recognition, predictive regulations, and process optimization (Gowd et al., 2023). Future progress depends on structured collaboration between bioenergy specialists and ML practitioners to refine algorithms, improve interpretability, and develop scalable applications. Overcoming these limitations will enable ML to substantially enhance bioenergy performance, optimize waste-to-energy conversion, and support sustainable sectoral transformation (Nega et al., 2024). Figure 1 illustrates the number of publications produced over the past decade, categorized by country. This investigation adopts a holistic perspective by assessing not only the immediate environmental and economic impacts of bioenergy interventions but also their scalability and long-term sustainability (Nazloo et al., 2022). In contrast, to traditional studies emphasizing short-term mitigation measures, the present work aligns with global decarbonization agendas, ensuring that proposed strategies support enduring climate commitments (Nikita et al., 2022). A distinguishing contribution of this research lies in its integration of circular bioeconomy concepts with decarbonization frameworks, delivering a comprehensive model for sustainable palm oil waste management that reconciles environmental stewardship with economic feasibility. A central aim is to accelerate decarbonization within the palm oil waste sector by aligning waste collection and processing practices with climate objectives, thereby reducing greenhouse gas (GHG) emission and advancing sustainable energy systems. The study further evaluates environmentally responsible strategies that enhance the ecological performance of the palm oil industry while simultaneously stimulating bioeconomy growth. This transition holds potential to create green employment opportunities, strengthening economic resilience alongside sustainable industrial advancements (Nikita and Nikitas, 2020). Additionally, this work provides an extensive review of biomass conversion technologies, discussing recent technological developments and identifying future research priorities. As biofuel technologies evolve, their contribution to reducing fossil fuel dependency and fostering a circular bioeconomy remains pivotal for global energy security and climate stability. Earlier research on biowaste remediation and valorisation has predominantly emphasized composting of leachate treatment, with comparatively limited focus on integrated biowaste-to-bioenergy systems. Although bioenergy research has progressed, investigations remain fragmented, particularly regarding the application of artificial intelligence (AI) in biowaste management (Ogunjuyigbe et al., 2017). AI-enabled technologies process transformative potential for biowaste remediation by optimizing energy recovery from lignocellulosic and algal biomass residues, thereby improving process sustainability and operational efficiency (Olabi, A.G. et al., 2020). Nonetheless, a consolidated review of AI-driven strategies in biowaste valorisation is currently lacking. Accordingly, this study addresses this gap by delivering a comprehensive evaluation of AI-based approaches for biowaste-to-bioenergy conversion, with particular emphasis on lignocellulosic and algal biomass streams. Through the application of emerging AI methodologies, the research aims to improve biowaste valorisation efficiency and advance a technologically sophisticated, sustainable bioeconomy (Oluwoye, I et al., 2020).

## 2 Support vector machine (SVM) algorithms in bioenergy applications

**FIGURE 2:**
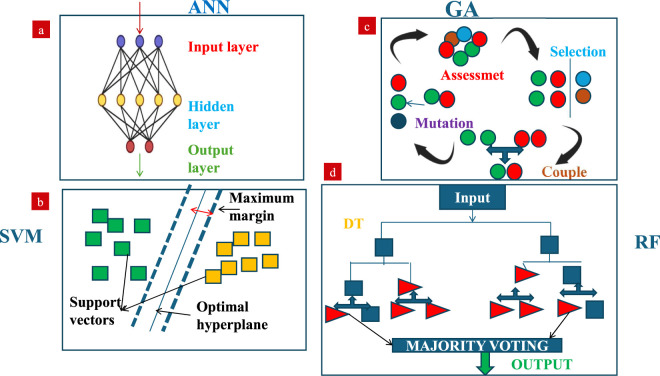
Four labeled diagrams represent machine learning algorithms: **(a)** artificial neural network with input, hidden, and output layers, **(b)** support vector machine with support vectors and optimal hyperplane, **(c)** genetic algorithm showing assessment, mutation, selection, and coupling, and **(d)** random forest with input, decision trees, majority voting, and output.

Support vector machine (SVM) algorithms are widely employed for multidimensional binary classification task (Figure 2a–c) and have proven effective in diverse regression and classification applications (Mancini et al., 2019; Umenweke et al., 2022). Their suitability for biological and bioenergy contexts stems from their capacity to capture complex nonlinear interactions within datasets. In contrast to traditional polynomial regression (PR), which minimize the sum of squared residuals between predicted and observed values, SVM minimizes an upper bound on generalization error, thereby improving predictive reliability in complex systems (Khan et al., 2023). A core feature of SVM is the application of kernel functions within support vector regression to define relationships between input variables and outputs. For binary classification, SVM identifies an optimal hyperplane that separates data into two categories. This boundary is determined by critical data points-termed support vectors-that maximize the margin between classes, ensuring stable and accurate classification (Umenweke et al., 2022). Recent developments demonstrate notable progress of SVM applications within the bioenergy field. Mancini et al. (2019) compared SVM with principal component linear discriminant analysis and partial least square discriminant analysis for biofuel classification non-destructive predictive models for evaluating lignocellulosic biofuel pellet quality. By integrating partial least squares regression with least squares SVM optimized via a successive projections algorithm (SPA), highly accurate estimations of key process variables were achieved, as reflected by strong coefficient of determination (Owumi et al., 2023). Desmal et al. (2022) further combined SVM with genetic algorithms to optimize via a successive projections’ algorithm. Support Vector Machine (SVM) algorithms are widely employed for multidimensional binary classification tasks (Figure 2a–c) and have proven effective in diverse regression and classification applications (Mancini et al., 2019; Umenweke et al., 2022). Their suitability for biological and bioenergy contexts stems from their capacity to capture complex nonlinear interactions within datasets. In contrast to traditional polynomial regression (PR), which minimizes the sum of squared residuals between predicted and observed values, SVM minimizes an upper bound on generalization error, thereby improving predictive reliability in complex systems (Khan et al., 2023). A core feature of SVM is the application of kernel functions within support vector regression to define relationships between input variables and outputs. For binary classification, SVM identifies an optimal hyperplane that separates data into two categories. This boundary is determined by critical data points–termed support vectors–that maximize the margin between classes, ensuring stable and accurate classification (Umenweke et al., 2022). Recent developments demonstrate notable progress of SVM applications within the bioenergy field. Mancini et al. (2019) compared SVM with principal component linear discriminant analysis and partial least squares discriminant analysis for biofuel classification, reporting superior performance for SVM. Similarly, Feng et al. (2018) established non-destructive predictive models for evaluating lignocellulosic biofuel pellet quality. By integrating partial least square regression with least squares SVM optimize via a successive projections algorithm (SPA), highly accurate estimations of key process variables were achieved, as reflected by strong coefficients of determination (Desmal et al., 2022; Owumi et al., 2023) further combined SVM with generic algorithms to optimize ethanol fermentation, achieving an ethanol production rate of 39, 598 kg/h under optimized conditions. These findings illustrate SVM’s versatility in classification, quality relatively low susceptibility to overfitting, SVM faces limitations when applied to large-scale datasets due to significant memory requirements and computational complexity, particularly when employing kernel transformations (Khan et al., 2023). Nonetheless, SVM remains a valuable analytical instrument for modelling and optimizing intricate bioenergy processes (Ozveren, 2017).

## 3 Different AI/ML algorithms

### 3.1 Artificial neural networks (ANN)

Artificial Neural Networks (ANNs) constitute a class of ML models inspired by the cognitive structure of the human brain (Figure 2b). Commonly characterized as black-box systems, ANNs apply gradient descent-based back propagation to estimate target outputs. Structurally, they comprise interconnected nodes organized into sequential layers until a final prediction is produced (Schmidhuber, 2015). ANNs emulate neural processing through nonlinear, adaptive, and fault-tolerant computational mechanisms (Guo et al., 2022). Feed-forward neural networks represent a prevalent architecture in which information flows unidirectionally from input to output layers (Andrade Cruz et al., 2022). Single-layer networks directly connect input and output nodes, whereas multilayer configurations incorporate hidden layers to capture more sophisticated data patterns. Network architecture is intrinsically linked to training strategy and model performances. The number of hidden neurons significantly influences data representation capacity, training convergence, and structural optimization. However, simply increasing neuron quantity does not guarantee enhanced learning performance (Faris et al., 2019). Excessive neurons may induce overfitting, while insufficient neurons can result in underfitting and diminished predictive precision (Alrashed et al., 2018). Effective ANN deployment therefore requires careful optimization of hidden layers and neuron counts, complemented by appropriate regularization strategies to balance complexity and generalization (Papadopoulos et al., 2023).

### 3.2 ANN application in algal systems

ANNs have been extensively implemented in predictive modelling for algal systems, where performance depends on accurate input-output mapping (Sundui et al., 2021). The input layer neuron count corresponds directly o the number of operational parameters used for model training (Figure 2a). Typical inputs include water depth, irradiance intensity, temperature, hydraulic retention time (HRT), dissolved oxygen (DO), total suspended solids (TSS), nutrients levels, and chemical oxygen demand (COD), all of which significantly influence algal biomass growth and treatment efficiency (Park et al., 2022). Hidden layers process these variables via activation functions to capture nonlinear interdependencies (Parkhey, 2022). The output layer represents system performance metrics such as biomass productivity or pollutant removal efficiency (Paul et al., 2014). Supriyanto et al. (2018) demonstrated the effectiveness of a multilayer back-propagation ANN in predicting microalgal growth in an open raceway pond. Using eight input variables–including initial biomass concentration, HRT, solar radiation, and nutrient availability–the model achieved an *R* ^2^ exceeding 0.93 for dry biomass prediction. These findings confirm ANN’s capability to model complex algal systems with high accuracy. By integrating multiple interrelated cultivated and wastewater remediation, thereby contributing to improved bioenergy and bioproduct development (Paulauskiene et al., 2019; Wu et al., 2022).

### 3.3 ANNs applications in algal systems

ANN-based predictive models for algal systems efficiently capture nonlinear interactions among environmental variables (Sundui et al., 2021). Input neurons correspond to selected process parameters, including pH, carbon dioxide (CO_2_) concentration, light intensity, temperature, hydraulic retention time (HRT), and dissolved oxygen demand (DOD), all of which influence biomass productivity and treatment performance. Hidden layers process these inputs through activation mechanisms to learn complex dependencies, and the output layer translates computations into metrics such as growth rate or system efficiency (Xia et al., 2023). Supriyanto et al. (2018) developed a multilayer back-propagation ANN incorporating eight core parameters for predicting microalgal biomass in an open raceway pond. The resulting model. Structured with a single hidden layer, achieved *R* ^2^ values above 0.93 demonstrating strong predictive capability. Such applications highlight ANN’s role in optimizing algal growth conditions and wastewater treatment processes, thereby strengthening sustainable bioenergy production and environmental management (Xing et al., 2021).

### 3.4 Different AI/ML algorithms

Support Vector Machines (SVM), introduced by Cortes and Vapnik in 1995, are supervised learning models extensively applied to regression and classification. The algorithm projects data into high-dimensional feature spaces through nonlinear mapping functions, where it determines an optimal separating hyperplane and nearest data points–support vectors–ensures robust decision boundaries (Figure 2b). Kernel functions enable SVM to manage nonlinear data structures, while adherence to the structural risk mitigation (SRM) principle commonly used in ANNs (Andrade Cruz et al., 2022). These properties make SVM particularly effective for precise predictive tasks. Applications include microalgal classification and wastewater treatment modelling. Hossain et al. (2022) demonstrated that SVM outperformed multilayer perception ANN (MLP-ANN) and response surface methodology (RSM) in predicting nitrogen and phosphorus removal efficiencies (Yek et al., 2021a; 2021b). However, SVM performance may decline with large or incomplete datasets, requiring careful preprocessing and kernel selection to maintain efficiency (Yew et al., 2020). Genetic Algorithm (GA): Genetic Algorithm are evolutionary optimization techniques based on neutral selection principles, operating through selection, crossover, and mutation mechanisms (Guo et al., 2021). Selection identifies high-fitness individuals, crossover recombines genetic information, and mutation introduces variability to maintain diversity (Figure 2c). These iterative processes continue until near optimal solutions are achieved. GA’s ability to circumvent local optima makes it suitable for complex optimization challenges. Camacho-Rodríguez et al. (2015) applied GA to optimize nutrient composition for cultivating *Nannochloropsis gaditana,* significantly reducing nutrient demand. Rodríguez-Miranda et al. (2021) modelled temperature effects in large scale microalgal cultures using GA, while Nayak et al. (2018) combined GA with ANN to enhance *Schenedesmus* sp. Biomass productivity by 57% during domestic wastewater treatment. Despite its flexibility, GA is computationally demanding and vulnerable to premature convergence. Careful parameter tuning and hybrid modelling approaches can mitigate these limitations (Yi, B et al., 2023; Yu D et al., 2022).

**FIGURE 3:**
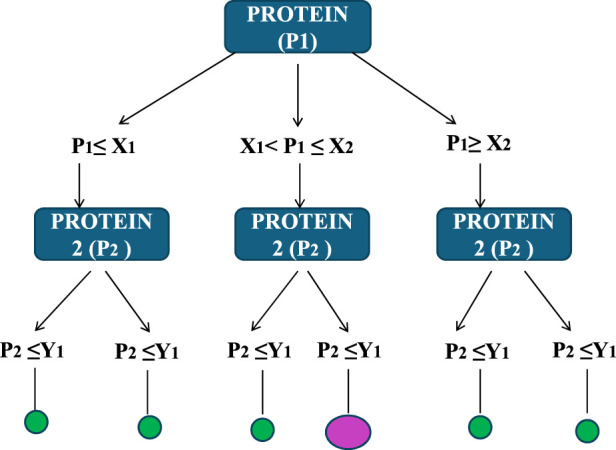
Flowchart illustrates decision pathways based on protein P1 levels divided into three conditions: P1 less than or equal to X1, P1 between X1 and X2, and P1 greater than or equal to X2. Each condition leads to assessment of protein P2, which is further split by whether P2 is less than or equal to Y1 or greater than Y1, resulting in colored circles at the end, including green and one purple.

Decision Tree (DT) and Random (RF) Algorithms: Decision Tree (DT) and Random Forest (RF) are supervised algorithms applied in both regression and classification contexts. DT structures data hierarchically, performing recursive binary splits to reduce internal variance and enhance pattern recognition (Figure 2d). Although DT models are interpretable and computationally efficient, they are prone to overfitting in complex scenarios (Zając, G et al., 2018). Random Forest mitigates this issue by constructing multiple uncorrelated trees through bagging and random feature selection, aggregating predictions via voting or averaging. This ensemble methodology improves generalization, robustness, and predictive accuracy, particularly for non-linear and high-dimensional datasets (Bagherzadeh et al., 2021). Applications include optimization of cultivation parameters for Trebouxiophyceae and Chlorophycae classes (Singh and Mishra, 2022), classification of viable versus non-viable *Chlorella vulgaris* populations (Reimann et al., 2020), and enhancement of bio-oil yield during hydrothermal liquefaction of microalgae (Figure 3) (Zhang et al., 2021). Collectively, DT and RF provide complementary strengths-interpretability and robustness making them effective tools for large-scale bioenergy modelling, wastewater treatment optimization and bioproduct extraction (Zhang, C et al., 2023; Zhang, L et al., 2023).

### 3.5 Critical evaluation of AI/ML methods in bioenergy systems

While a wide range of artificial intelligence (AI) and machine learning (ML) techniques have been applied in bioenergy systems, their effectiveness varies significantly depending on data availability, system complexity, and the specific application domain. A critical evaluation of commonly used approaches Artificial Networks (ANN), Support Vector Machines (SVM), and Genetic Algorithm (GA) is therefore essential to guide method selection. Artificial Neural Networks (ANN) have emerged as one of the most dominant tools in bioenergy applications, primarily due to their ability to model highly nonlinear relationships between process variables and system outputs. Bioenergy processes such as anaerobic digestion, biomass gasification, and bioethanol fermentation involve complex interactions between feedstock composition, microbial activity, temperature, and reaction kinetics. In such contexts, ANN models are particularly effective because they can learn patterns directly from data without requiring explicit mechanistic formulations. This capability aligns with broader findings in energy materials research, where machine learning significantly accelerates prediction and discovery by handling high-dimensional and nonlinear datasets. Nevertheless, despite their predictive strength, ANN models are not without limitations. They typically require large, high-quality datasets for training, which may not always be available in emerging bioenergy systems or pilot-scale operations. Additionally, ANN models are often criticized for their lack of interpretability, making it difficult to link predictions to underlying biochemical or thermodynamic mechanisms. This limitation restricts their ability to provide actionable insights for process understanding, even when prediction accuracy is high. Furthermore, issues such as overfitting and computational complexity can arise, particularly when model architecture is not carefully optimized (Liu et al., 2020). In contrast, Support Vector Machines (SVM) offer a more structured and mathematically robust approach, particularly in situations where data availability is limited. SVM models are well known for their strong generalization capabilities and their effectiveness in handling high-dimensional feature spaces. In bioenergy systems, SVM has been successfully applied in tasks such as predicting biofuel yields, classifying feedstock properties, and detecting anomalies in reactor operations. Compared to ANN, SVM models are less prone to overfitting and can achieve reliable performance with smaller datasets. This characteristic is particularly advantageous in experimental or early-stage bioenergy systems where data collection is constrained. However, SVM models also present challenges. Their performance is highly sensitive to the selection of kernel functions and hyperparameters, requiring careful tuning and domain expertise. Additionally, SVM models can become computationally intensive when applied to large datasets, limiting their scalability for industrial-scale bioenergy systems. While SVM can capture nonlinear relationships through kernel transformations, its flexibility remains lower than that of ANN when dealing with extremely complex and highly coupled processes (Otálora et al., 2021).

Genetic Algorithms (GA), on the other hand, represent a fundamentally different category of AI methods, as they are primarily optimization techniques rather than predictive models. GA has been widely used in bioenergy applications for optimizing process parameters, reactor configurations, and supply chain logistics. Its strength lies in its ability to explore large and complex search spaces without requiring gradient information, making it particularly suitable for multi-objective optimization problems. For example, GA can simultaneously optimize biofuel yield, energy efficiency, and emission reduction, which are often conflicting objectives in bioenergy systems. This capability aligns with broader energy system optimization strategies, where hybrid approaches combining predictive models and optimization algorithms have demonstrated substantial performance improvements. In energy system studies, integrating surrogate models with optimization frameworks has been shown to reduce computational time by up to 84% while maintaining solution accuracy. Despite these advantages, GA also has notable limitations. The optimization process can be computationally expensive and time-consuming, particularly for large-scale systems with multiple decision variables. Additionally, GA does not provide direct predictive capabilities and must be combined with models such as ANN or SVM to evaluate system performance. As a result, GA is most effective when used as part of a hybrid framework rather than as a standalone tool. The comparative analysis clearly indicates that no single AI/ML method can be considered universally optimal for all bioenergy applications. Instead, the suitability of each method depends on the specific characteristics of the problem. ANN models are best suited for data-rich environments with highly nonlinear relationships, where prediction accuracy is the primary objective. SVM models are more appropriate for smaller datasets requiring robust generalization and stability. GA is most effective for optimization problems involving multiple objectives and complex decision spaces. Increasingly, hybrid approaches that combine these methods are being adopted to leverage their complementary strengths. For instance, ANN-GA frameworks enable accurate prediction combined with efficient optimization, while SVM-GA models can improve robustness in parameter tuning. Such hybrid strategies are consistent with broader trends in AI-enabled energy systems, where combining predictive modeling with optimization significantly enhances performance and scalability. Another important consideration highlighted in the revised manuscript is the relationship between AI predictions and physical system behavior. While AI models can achieve high predictive accuracy, their practical value in bioenergy systems depends on their ability to reflect underlying physical and biochemical mechanisms. For example, parameters such as temperature, substrate concentration, and microbial kinetics are not merely inputs to a model but are directly linked to reaction pathways and system efficiency. Therefore, future research should focus on integrating AI models with physics-based approaches to improve interpretability and reliability. This perspective is consistent with recent advancements in energy systems and materials science, where combining data-driven models with mechanistic understanding is considered essential for achieving scalable and sustainable solutions. Furthermore, the broader challenges associated with AI adoption in bioenergy systems, including data availability, model interpretability, and scalability. As noted in energy-efficient manufacturing and energy system studies, the effectiveness of AI depends heavily on access to high-quality data and the ability to translate model outputs into actionable insights. Addressing these challenges requires the development of standardized datasets, explainable AI techniques, and hybrid modeling frameworks that combine data-driven and physics-based approaches. In summary, the manuscript has been significantly strengthened by incorporating a critical and comparative evaluation of ANN, SVM, and GA methods. The revised section not only highlights their individual strengths and limitations but also provides clear guidance on their suitability for different bioenergy applications.

## 4 Microalgal screening and classification

The identification and selection of suitable strains constitute a fundamental perquisition for achieving economically viable and sustainable production systems. Machine learning (ML) methodologies have demonstrated considerable utility in species recognition and classification tasks. Otálora et al. (2021) integrated an Artificial Neural Network (ANN) framework with FlowCAM-based image analysis to distinguish *Chlorella vulgaris* and *Scenedesmus almerirnsis* within mixed cultured. Similarly, Liu et al. (2020) implemented a genetic algorithm-optimized backpropagation ANN to monitor *Chalmydomonas reinhardtii* concentration using fluorescence spectral signatures. Research further emphasis the critical influence of micronutrients and vitamins on microalgal proliferation. López-Rosales et al. (2013) applied feed-forward backpropagation neural networks (FBN) to stimulate interactions among 26 culture medium constitutes, demonstrating that micronutrients and vitamins exerted greater influence than macronutrients on *Protoceratium recticulatum* growth. In addition, Garcia-Camacho et al. (2016) confirmed that micronutrient availability significantly shaped growth kinetics in *Karlodinium veneficum* cultures.

### 4.1 Light intensity optimization

Light intensity represents a key determinant of photosynthetic efficiency and microalgal productivity. AI/ML-based models have been extensively deployed to regulate and optimize illumination conditions. Hu et al. (2008) developed an ANN-model Predictive Control (ANNMPC) systems capable of dynamically adjusting irradiance levels for *Spirulina platensis* cultivated in continuous photobioreactors. Mowbray, M et al. (2021) modelled the combined effects of light intensity and nitrate concentration to enhance lutein biosynthesis in microalgae, reporting yield increase of 40%–50%. Automation incorporation image processing algorithms substantially reduces labor requirement and operational time in microalgal cultivation systems. Microscopy platforms integrated with AI/ML models facilitate real-time evaluation of parameters such as cell morphology, size distribution, and density within photobioreactors. Giraldo-Zuluaga et al. (2018) employed ANN and Support Vector Machine (SVM) approaches to accurately identify *Scenedesmus coenobin* through advanced image processing techniques. Collectively, ML algorithms provide transformative tools for optimizing microalgal cultivation and wastewater remediation. Through reductive modelling of operational parameters, and productive enhancement, AI/ML technologies are accelerating the development of efficient and sustainable microalgal platforms. Continued investigation into these methodologies is expected to further unlock the industrial and environmental potential of microalgae.

### 4.2 Mechanistic interpretation of AI models in bioenergy systems

**FIGURE 4:**
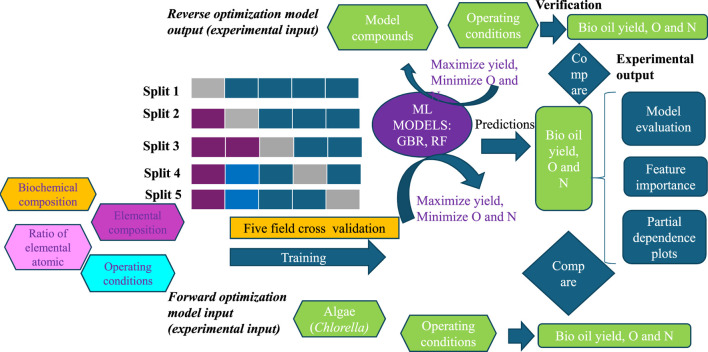
Flowchart illustrating a machine learning workflow for predicting bio-oil yield, oxygen, and nitrogen content from algae. The diagram includes process inputs such as biochemical composition, elemental composition, and operating conditions, depicted with colored hexagons. Data splits for cross-validation are shown using colored bars labeled Split 1 through Split 5. Central elements include ML models (GBR, RF) with arrows indicating training and prediction steps to maximize yield and minimize oxygen and nitrogen. Outputs are verified and evaluated with additional steps for model evaluation, feature importance, and partial dependence plots.

A key limitation in many AI-assisted bioenergy studies is the insufficient connection between model predictions and the underlying physical or chemical mechanisms governing system behavior. While machine learning models can achieve high predictive accuracy, their scientific and engineering relevance depends on how effectively the relationships between input parameters, predicted outputs, and process-level phenomena are interpreted. In bioenergy systems, input variables typically represent measurable physical or chemical conditions, such as feedstock composition (e.g., cellulose, hemicellulose, lignin content), operating temperature, pH, residence time, catalyst loading, and microbial population dynamics. These inputs are directly associated with fundamental processes including reaction kinetics, mass transfer, heat transfer, and biochemical conversion pathways. The outputs of AI models such as biofuel yield, conversion efficiency, reaction rate, energy output, or system stability are therefore not abstract predictions but quantitative reflections of these underlying mechanisms. In anaerobic digestion, input parameters such as substrate concentration, temperature, and pH influence microbial activity and metabolic pathways. AI models such as Artificial Neural Networks (ANN) learn the relationship between these inputs and outputs such as methane yield or biogas production rate. Mechanistically, temperature affects enzymatic reaction rates, pH influences microbial community stability, and substrate concentration determines the availability of biodegradable organic matter. Thus, when an ANN predicts increased methane production under specific temperature and pH conditions, it is implicitly capturing the enhanced activity of methanogenic bacteria and improved substrate conversion efficiency. This illustrates how AI outputs can be interpreted as proxies for biochemical reaction dynamics rather than purely statistical correlations (Figure 4). Similarly, in thermochemical conversion processes such as biomass gasification or pyrolysis, input variables including temperature, heating rate, and feedstock composition determine reaction pathways such as devolatilization, cracking, and char formation. AI models trained on these variables can predict outputs such as syngas composition, tar formation, or energy efficiency. From a mechanistic perspective, higher temperatures promote endothermic reactions and increase gas yield, while feedstock composition influences the relative production of CO, H_2_, and hydrocarbons. Therefore, AI predictions of syngas composition can be directly linked to reaction thermodynamics and kinetics. This type of interpretation transforms AI models into tools that complement, rather than replace, conventional process understanding Hu et al. (2008).

The importance of linking AI outputs to physical mechanisms is also evident in energy system optimization studies, where surrogate models are used to approximate complex engineering simulations. For instance, ANN-based surrogate models have been developed to replace computationally intensive energy system models, enabling faster optimization of system design and operation. These models take inputs such as energy demand profiles, renewable resource availability, and system configuration parameters, and predict outputs such as cost, efficiency, or grid interaction levels. Notably, hybrid optimization approaches combining surrogate models with engineering models have demonstrated substantial improvements in computational efficiency, reducing simulation time by up to 84% while maintaining solution accuracy. In such cases, the AI model does not simply provide predictions; it captures the relationship between system-level inputs and performance metrics governed by energy balance equations, resource variability, and operational constraints. In the context of bioenergy supply chains and integrated systems, AI models also incorporate variables such as transportation distance, feedstock availability, process efficiency, and energy demand. These variables are linked to physical processes including energy conversion, resource logistics, and system-level energy flows. AI predictions of system efficiency or cost therefore reflect the combined effects of thermodynamic performance, process integration, and operational optimization. As noted in broader energy system studies, AI plays a critical role in improving energy efficiency, forecast demand, and optimizing resource allocation by learning from complex, multi-variable datasets (Hu et al. 2008).

Another important aspect is the role of AI in materials and catalyst optimization for bioenergy applications. Machine learning models can use input descriptors such as surface area, pore size distribution, chemical composition, and electronic properties to predict catalytic activity or conversion efficiency. These descriptors are directly related to reaction mechanisms such as adsorption, diffusion, and catalytic transformation. For instance, higher surface area and optimal pore structure enhance reactant accessibility and mass transfer, leading to improved reaction rates. Thus, AI predictions in this domain can be interpreted in terms of physicochemical interactions at the molecular level. This perspective is consistent with developments in energy materials research, where machine learning has been used to accelerate the discovery and optimization of materials by linking structural descriptors to functional performance. Despite these advances, it is important to recognize that many AI models remain “black-box” systems, which can obscure the connection between inputs and outputs. This lack of transparency can limit their usefulness in scientific research, where understanding causal relationships is essential. To address this issue, the revised manuscript emphasizes the importance of explainable AI techniques, such as sensitivity analysis, feature importance ranking, and SHAP (Shapley Additive Explanations) methods. These tools allow researchers to identify which input variables have the greatest influence on model predictions, thereby providing insights into the dominant physical or chemical processes. Furthermore, the integration of AI with physics-based models is highlighted as a promising direction for improving interpretability and reliability. Hybrid approaches that combine data-driven models with first-principles equations can ensure that predictions remain physically consistent while benefiting from the flexibility of machine learning. Such approaches are particularly valuable in bioenergy systems, where experimental data may be limited and process conditions are highly variable. These systems involve multiple interacting subsystems, including feedstock processing, conversion technologies, energy distribution, and end-use applications. In such cases, AI models capture system-level behavior rather than individual process mechanisms. Nevertheless, by carefully selecting input variables and interpreting model outputs in the context of known physical principles, it is possible to extract meaningful insights that support system optimization and design.

## 5 Bioenergy production and biowaste-to-bioenergy supply chain and feedstock selection

Bioenergy derived from biomass includes oil-based, liquid, and gaseous fuels, with biodiesel and bioethanol representing the most widely adopted forms. These biofuels are primarily utilized in transportation blends as well as for heating and electricity generation. Several nations-including China, India, Malaysia-have initiated biodiesel production programs; however, the United States remains the global leader, contributing approximately 41% of total biofuel output. Other major producers include Brazil, Indonesia, China, and France (Zhao et al., 2016). The biowaste-to-bioenergy conversion framework operates through an interconnected supply chain. The initial phased involves feedstock sourcing, securing biomass resources such as agricultural residues, forestry by-products, and organic industrial waste to ensure consistent availability (Zhao et al., 2016). The processing stage converts these materials into bioenergy through pathways such as anaerobic digestion, pyrolysis, and fermentation, emphasizing production efficiency and waste minimization (Zhu et al., 2016). The final stage encompasses end users, including industries and household employing bioenergy for electricity, heating, or alternative fuel applications. Ensuring cost-effectiveness and environmental sustainability remains central to broad market adoption.

**FIGURE 5:**
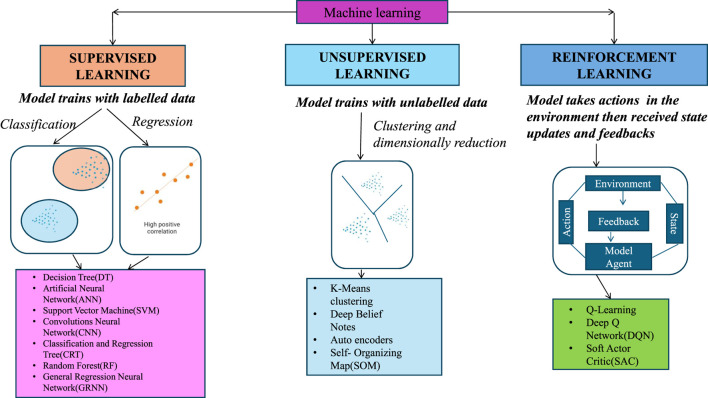
Flowchart illustrating three main types of machine learning: supervised learning with labeled data, unsupervised learning with unlabeled data, and reinforcement learning with environment feedback. Each type includes examples of key algorithms.

Feedstock selection is a pivotal determinant within the bioenergy supply chain, directly influencing both sourcing strategies and processing efficiency. Biomass feedstocks are categorized into four generations based on sustainability considerations and implications for food security. First-generations biofuels are derived from edible crops such as corn, wheat, and rice. Although readily available and easy processed, these feedstocks raise ethical concerns due to competition with food supplies (Zhao et al., 2016). Second-generation biofuels utilize non-food biomass, including dedicated energy crops, agriculture residues, and industrial by-products, thereby alleviating food-versus fuel conflicts and enhancing resource efficiency (Zhu et al., 2016). Third-generation bioenergy relies on algal biomass cultivated in freshwater or wastewater environments, offering high productivity and reduced reliance on arable land (Zhu et al., 2016). Fourth generation of bioenergy involves genetically engineered microorganisms optimized for fuel production, improving conversion efficiency and sustainability (Zhao et al., 2016). Among these options, lignocellulosic and algal feedstock demonstrate strong sustainability potential. While first generation sources face increasing scrutiny due to food security implications, second- and third-generation alternatives provide enhanced environmental and economic advantages. For example, wastewater-based algal cultivation has been shown to reduce algal oil production cost from $3.90 to $0.54 per litre while achieving yields exceeding 80%. Likewise, lignocellulosic conversion efficiencies have reached up to 95% (Zhu et al., 2016). Nevertheless, feedstock selection remains constrained by cultivation practices, geographic limitations, and processing challenges. Addressing these factors is essential to optimizing biowaste-to bioenergy systems and ensuring long-term sustainability (Zhao et al., 2016). The incorporation of AI technologies across bioenergy systems stages has advanced considerably, particularly in process control, yield forecasting, real-time monitoring, and operational optimization (Figure 5). Biomass conversion underpins these systems and can be classified into five principal pathways: physical, biological, chemical, biochemical, and thermochemical processes.

### 5.1 Physical conversion

Physical treatments employ mechanical forces without introducing chemical or biological agents. Typically implemented during preliminary stages, these processes include grinding, sieving, pelletizing, compaction, and densification to enhance uniformity and energy density. Particle Size Reduction: reducing particle size below 1,000 µm has been shown to improve bio-oil syngas yields during pyrolysis and steam reforming. Densification: increasing energy density lowers storage and transportation costs. AI systems are frequently applied to monitor parameters such as dimensions, geometry, and moisture content, ensuring optimized storage and downstream handling.

### 5.2 Biological conversion

Biological pathways utilize living organisms to convert organic substrate into biofuels such as biodiesel, bioethanol, biogas, and hydrogen. Microbial fermentation remains a dominant approach, employing starch- and sugar-rich feedstocks for bio-liquid fuel production. Microorganisms in Fermentation: prominent species include *Saccharomyces cerevisiae* and *Pediococcus pentosaceus.* Sewage Sludge Utilization: Aerobic and anaerobic treatments convert sludge into biogas while recovering biofuels from lipid-rich feedstocks, including algal biomass and animal-derived residues.

### 5.3 Chemical conversions

Chemical conversion encompasses extraction, catalytic reactions, and transesterification for producing biofuels from lipid-rich feedstock, including algal biomass and animal-derived residues.

Catalytic Processes: Catalyst such as calcium oxide (CaO) and sodium hydroxide (NaOH) have achieved biodiesel yields exceeding 90%, with optimized transesterification reaching up to 99.8%. Catalyst accelerated reaction kinetics and improved conversion efficiency. AI optimization: AI-based computational models identify optimal catalyst types, solvent ratios, reaction durations, and operational parameters, enhancing yield, minimizing waste and reducing costs.

### 5.4 Biochemical conversion

Biochemical processes employ microorganisms and enzymes to decompose organic constituents within biomass. Anaerobic Digestion: converts biomass into biogas containing 40%–65% methane alongside carbon dioxide and trace gases, offering renewable energy while reducing organic waste (Nazloo et al., 2022). Aerobic Processes: Degrade organic matter in the presence of oxygen, generating carbon dioxide, water, and nutrient-rich sludge suitable for agricultural reuse. AI in Bioprocessing: AI tools predict fermentation dynamics, design enzymatic pathways, and simulate complex bioprocesses, improving efficiency and reducing operational costs (Muzyka, R et al., 2023).

### 5.5 Thermochemical conversion

Thermochemical routes apply heat to alter biomass properties and generate energy-dense products, including torrefaction, pyrolysis, gasification, and liquefication. Torrefaction: Conducted at 200 °C–300 °C under oxygen-limited conditions, torrefaction enhances biochar porosity, increasing calorific value, and improves hydrophobicity and storage stability (Hu et al. 2008). Pyrolysis and Gasification: Pyrolysis decomposes biomass in oxygen-free environments into bio-oil, syngas, and biochar. Gasification involves partial oxidation to produce syngas composed primarily of hydrogen and carbon monoxide, which can be converted into fuels or electricity. Additive treatments further improve product quality (Del Rio-Chanona et al., 2019). AI Optimization: AI-driven predictive models optimize temperature, residence time, and additive concentration, improving energy yields, product characteristics, and economic viability.

### 5.6 Emerging applications and AI integration

Across all conversional pathways, AI serves as a central role in performance optimization, production forecasting, and cost reduction. AI based frameworks assists in cultivation design, real-time parameter monitoring, and techno-economic evaluation of biowaste to bioenergy systems (Mathur M et al., 2022; Xiong, Z et al., 2022). These developments demonstrate AI’s transformative capacity to scale bioenergy production sustainability.

#### 5.6.1 AI–energy materials interface in energy storage systems

The performance of energy storage systems, including batteries and supercapacitors, is intrinsically linked to the design and behavior of advanced energy materials. While AI techniques have been widely applied to predict system-level performance, their true potential lies in enabling a deeper understanding and optimization of material properties and their interaction with electrochemical processes. In this context, a more technically grounded perspective is required to connect AI methodologies with the fundamental mechanisms governing energy storage. At the material level, key input parameters in AI models typically include descriptors such as particle size, surface area, porosity, crystal structure, electronic band structure, chemical composition, and defect density. These parameters directly influence transport phenomena, including ion diffusion and electron conduction, as well as interfacial reactions that determine charge storage and energy conversion efficiency. For example, in lithium-ion batteries, electrode materials must facilitate efficient lithium-ion intercalation and deintercalation while maintaining structural stability over repeated cycles. AI models trained on structural and compositional descriptors can predict properties such as capacity, voltage profiles, and degradation rates. However, these predictions are fundamentally linked to underlying processes such as ion diffusion pathways, phase transformations, and solid electrolyte interphase (SEI) formation. Recent developments in machine learning for energy materials demonstrate how data-driven approaches can significantly accelerate material discovery and optimization. For instance, machine learning models have been used to screen large material spaces by correlating physicochemical descriptors with performance metrics. In one representative example, Gaussian process-based models utilized permeability data from approximately 700 polymer samples to predict the behavior of more than 11,000 untested materials, thereby expanding the search space far beyond traditional experimental approaches. Such approaches illustrate how AI can capture structure property relationships that are otherwise difficult to explore using conventional methods. Importantly, these predictions are not arbitrary; they reflect fundamental interactions such as molecular packing, transport resistance, and thermodynamic stability. In the case of supercapacitors, energy storage is governed by mechanisms such as electric double-layer formation and pseudocapacitive reactions. Material properties such as specific surface area, pore size distribution, and electrical conductivity play a critical role in determining capacitance and charge discharge behavior. AI models that incorporate these descriptors can predict capacitance and cycling stability. Mechanistically, a higher surface area increases the number of active sites for charge accumulation, while optimized pore structures facilitate ion transport and reduce diffusion limitations. Therefore, AI predictions of capacitance can be directly linked to electrostatic interactions and ion-accessible surface area within the electrode material.

The integration of advanced manufacturing techniques, such as additive manufacturing and emerging 4D printing technologies, further highlights the importance of material-level considerations in energy storage systems. These technologies enable precise control over material architecture, including porosity, geometry, and spatial distribution of active components. As discussed in recent studies, additive manufacturing allows for the fabrication of complex electrode structures that improve mass transport and reduce material waste, while 4D printing introduces the possibility of dynamic, stimuli responsive materials capable of adapting to operating conditions. However, despite these advantages, challenges such as material compatibility, structural stability, and scalability remain significant. AI can play a critical role in addressing these challenges by optimizing material formulations and processing parameters to achieve desired structural and functional properties. Another important aspect of the AI materials interface is the relationship between material degradation and long-term performance. In battery systems, degradation mechanisms such as electrode cracking, SEI growth, and electrolyte decomposition lead to capacity fading and reduced cycle life. AI models trained on operational and material data can predict degradation trends and identify key factors influencing failure. These predictions can be interpreted in terms of physical processes such as mechanical stress accumulation, chemical instability, and diffusion limitations. By linking model outputs to these mechanisms, AI can provide actionable insights for improving material durability and system reliability (Mathur et al., 2022).

Furthermore, the application of AI in energy storage systems must consider the broader system-level implications of material performance. For example, optimizing material for high energy density may lead to tradeoffs in safety or cycle life. AI-based optimization frameworks, particularly those incorporating multi objective algorithms, can evaluate these trade-offs by simultaneously considering multiple performance metrics. In energy system optimization studies, hybrid approaches combining surrogate models and optimization algorithms have demonstrated the ability to efficiently explore complex design spaces while maintaining high accuracy. Such approaches can be extended to material design, enabling the identification of optimal combinations of properties for specific applications. Despite these advances, challenges remain in fully integrating AI with energy materials research. One of the primary limitations is the availability and quality of data, particularly for emerging materials and novel chemistries. Additionally, many AI models lack interpretability, making it difficult to establish clear causal relationships between material descriptors and performance outcomes. To address these challenges, the revised manuscript highlights the importance of explainable AI techniques and the integration of data driven models with physics-based approaches. This hybrid strategy ensures that predictions remain consistent with fundamental principles while benefiting from the flexibility and scalability of machine learning.

## 6 Microalgae derived materials for energy storage applications

Microalgae derived materials have emerged as a promising class of sustainable and high-performance candidates for electrochemical energy storage systems, including batteries and supercapacitors. Unlike conventional synthetic precursors, microalgae biomass inherently contains a rich composition of carbon, nitrogen, oxygen, and trace elements, which can be transformed into functional materials with desirable electrochemical properties through controlled thermal and chemical processing. The integration of such bio-derived materials into energy storage devices offers a dual advantage of sustainability and performance enhancement. One of the most widely explored applications of microalgae derived materials is in the synthesis of porous carbon electrodes. During pyrolysis, the organic components of microalgae decompose to form carbon-rich structures with hierarchical porosity. This porosity is critical for electrochemical performance, as it determines the accessibility of electrolyte ions to active sites. Micropores contribute to high surface area and charge storage capacity, while mesopores and macropores facilitate ion transport and reduce diffusion resistance. Consequently, microalgae derived carbons often exhibit enhanced specific capacitance and improved rate capability when used in supercapacitors. Mechanistically, the high surface area increases the formation of electric double layers, while optimized pore networks enable rapid ion adsorption and desorption during charge discharge cycles.

In addition to structural advantages, microalgae-derived carbons frequently exhibit heteroatom doping, particularly with nitrogen, sulfur, and phosphorus. These heteroatoms originate from the protein and lipid content of the biomass and are retained during carbonization. Nitrogen doping, for example, introduces defects and active sites within the carbon lattice, which can enhance electrical conductivity and promote pseudocapacitive behavior. From an electrochemical perspective, these doped sites facilitate faradaic reactions, contributing to additional charge storage beyond purely electrostatic mechanisms. As a result, microalgae-derived electrodes can achieve higher capacitance compared to undoped carbon materials. This phenomenon reflects the direct relationship between material composition and electrochemical functionality, highlighting the importance of tailoring precursor composition for targeted performance. Microalgae derived materials have also been investigated for battery electrode applications, particularly in lithium-ion and sodium-ion systems. In these systems, the performance of electrode materials depends on their ability to accommodate ion insertion and extraction without significant structural degradation. The porous and flexible nature of bio-derived carbon structures allows them to buffer volume changes during cycling, thereby improving structural stability and cycle life. Furthermore, the presence of defects and heteroatoms can enhance ion adsorption and diffusion kinetics, leading to improved capacity and rate performance. Mechanistically, these effects are linked to the availability of active sites for ion storage and the reduction of energy barriers for ion transport within the electrode matrix.

Beyond carbon based materials, microalgae can also serve as precursors for composite and hybrid materials, where bio-derived carbon is combined with metal oxides or conductive polymers to enhance electrochemical performance. For example, integrating microalgae-derived carbon with transition metal oxides can improve both electrical conductivity and redox activity. In such composites, the carbon matrix provides a conductive network and structural support, while the metal oxide contributes to faradaic charge storage. This synergistic interaction results in improved energy density and cycling stability. From a mechanistic standpoint, the composite structure facilitates efficient electron transport and enhances the utilization of active materials. The relevance of microalgae-derived materials is further amplified when considered in the context of advanced manufacturing and material design strategies. Emerging fabrication techniques, including additive manufacturing and architected electrode design, enable precise control over material structure and morphology. As highlighted in recent studies, advanced manufacturing approaches can significantly influence mass transport, mechanical stability, and overall device performance. When combined with bio-derived materials, these approaches open new opportunities for designing electrodes with optimized porosity, conductivity, and structural integrity. Artificial intelligence can further enhance this process by identifying optimal processing conditions and material compositions that maximize electrochemical performance.

Another critical aspect of microalgae-derived materials is their role in sustainable energy systems. The use of renewable biomass reduces reliance on fossil-based precursors and contributes to carbon-neutral or carbon negative material production pathways. Moreover, the ability to tailor microalgae cultivation conditions allows for controlled modification of biomass composition, which can influence the properties of the resulting materials. For example, nutrient availability, growth conditions, and species selection can affect lipid and protein content, thereby impacting carbon yield and heteroatom doping levels. This tunability provides an additional layer of control in material design, which can be leveraged to optimize electrochemical performance. Despite these advantages, challenges remain in translating microalgae-derived materials from laboratory-scale demonstrations to practical energy storage systems. Variability in biomass composition, scalability of processing methods, and reproducibility of material properties are key issues that need to be addressed. Additionally, the relationship between precursor characteristics, processing conditions, and final material performance is complex and requires systematic investigation. In this context, data-driven approaches and machine learning techniques can play a crucial role in establishing structure property performance relationships and guiding material optimization. As demonstrated in broader energy materials research, machine learning can significantly accelerate the discovery and design of functional materials by linking input descriptors to performance outcomes. In summary, the manuscript provides a more detailed and technically grounded discussion of microalgae-derived materials for energy storage applications. By explicitly linking material properties such as porosity, heteroatom doping, and structural stability to electrochemical mechanisms including double-layer formation, pseudo capacitance, and ion diffusion, the manuscript now offers a clearer understanding of how these materials contribute to device performance.

Machine learning (ML) enabled techno-economic analysis (TEA) frameworks for microalgal biofuel production can be significantly strengthened by systematically integrating knowledge across multiple interconnected research domains, including algal biorefinery engineering, catalytic conversion processes, wastewater remediation, advanced functional materials, environmental sensing technologies, and sustainability-driven risk assessment. Microalgae have emerged as a highly promising renewable feedstock due to their rapid biomass accumulation, superior carbon sequestration efficiency, and ability to grow in non-arable and wastewater-based environments, thereby minimizing competition with food resources and freshwater demand. These intrinsic advantages make microalgae particularly suitable for the development of integrated and circular bioenergy systems, enabling simultaneous resource recovery and environmental remediation (Chi et al., 2021; Ethiraj and Samuel, 2024; Ganesan et al., 2020; Pusuluru et al., 2026a; Samuel et al., 2024). Furthermore, recent advancements in algal biorefinery optimization, including polysaccharide utilization strategies and AI-driven process enhancement, have demonstrated the potential to improve conversion efficiencies and economic feasibility of microalgal biofuel pathways (Chang et al., 2025; Pusuluru et al., 2026a). Simultaneously, the development of advanced materials—particularly carbon-based nanostructures, graphene derivatives, metal–organic frameworks (MOFs), and hybrid catalytic systems has significantly enhanced both catalytic activity and pollutant removal performance in environmental and energy systems. Graphene-based nanocomposites, for example, exhibit high surface area, electrical conductivity, and tunable surface chemistry, enabling improved adsorption and catalytic efficiency in aqueous systems (Samuel et al., 2018; Samuel et al., 2021; Samuel et al., 2022; Singh et al., 2024c; Nagendran et al., 2024). Similarly, biochar-derived catalysts and engineered porous materials have been extensively explored for biofuel production from algal biomass, offering improved reaction kinetics and energy conversion pathways (Anto et al., 2021; Chi et al., 2021). The integration of such materials into microalgal processing systems contributes to enhanced feedstock quality, improved contaminant removal, and optimized catalytic conversion, thereby strengthening overall system sustainability.

In wastewater-integrated algal cultivation systems, the presence of heavy metals, organic pollutants, and emerging contaminants introduces additional complexity that must be addressed through robust treatment strategies. Extensive studies on adsorption, bioremediation, and advanced oxidation processes provide critical datasets that can be incorporated into ML models to improve predictive accuracy and TEA robustness under realistic operational scenarios (Abigail et al., 2016; Samuel and Chidambaram, 2015; Samuel et al., 2020; Samuel et al., 2021; Datta et al., 2020; Datta et al., 2021; Samuel et al., 2023). Additionally, the application of enzyme immobilization and microbial-based catalytic systems further enhances pollutant degradation and biomass conversion efficiency, providing additional variables for ML assisted optimization (Rajnish et al., 2021; Selvarajan et al., 2025). These developments collectively enable the integration of biochemical, physicochemical, and process-level data into TEA frameworks, resulting in more accurate and scalable system evaluations. The growing concern regarding persistent and emerging contaminants, particularly per- and polyfluoroalkyl substances (PFAS), underscores the importance of incorporating environmental risk and regulatory compliance into ML-assisted techno-economic frameworks. The transformation, persistence, and potential toxicity of such contaminants during thermal, catalytic, and electrochemical treatment processes must be carefully evaluated to ensure both environmental safety and regulatory alignment (Natishah et al., 2025; Samuel et al., 2024; Singh et al., 2025). Moreover, groundwater contamination dynamics, trace element mobility, and variability in water quality further emphasize the need to expand TEA system boundaries beyond conventional economic metrics to include longterm environmental sustainability considerations (Natishah et al., 2025; Punna et al., 2026; Singh et al., 2025).

Advancements in sensing technologies and analytical platforms have further enhanced the integration of ML within TEA frameworks by enabling real-time monitoring and adaptive process control. Electrochemical sensors, nanomaterial-based detection systems, and surface-enhanced spectroscopic techniques have demonstrated high sensitivity and selectivity for detecting environmental contaminants and process intermediates (Balaji et al., 2021; Maheshwaran et al., 2022a; Maheshwaran et al., 2022b; Maheshwaran et al., 2022c; Sivasubramanian et al., 2024). The incorporation of such real-time data streams into ML models allows for dynamic optimization of operating conditions, improved process stability, and enhanced predictive capabilities. In addition, advances in nanocomposite-based sensing materials, including heterojunction systems and hybrid catalytic platforms, further contribute to improved detection performance and system responsiveness (Balaji et al., 2022; Maheshwaran et al., 2022a). From a broader sustainability perspective, the integration of circular material systems into microalgal biorefineries represents a critical advancement in resource recovery and waste valorization. The transformation of plastic waste into functional carbon nanomaterials, the development of bio-derived graphene, and the application of smart polymers for water treatment collectively support circular economy principles and reduce environmental impact (Pusuluru et al., 2026e; Nagendran et al., 2024; Pusuluru et al., 2026b). Furthermore, the incorporation of advanced catalytic systems, including nano-catalysts for CO_2_ reduction and enzymatic catalysts for bioenergy generation, enhances overall system efficiency and contributes to carbon-neutral or carbon-negative energy production pathways (Chang et al., 2024; Senthilkumar et al., 2024). Additional innovations in ammonia synthesis and hydrogen economy integration further demonstrate the potential of coupling bioenergy systems with emerging clean energy technologies (Sekhar et al., 2024; Singh et al., 2024c). The integration of advanced materials and catalytic systems into bioenergy processes also extends to corrosion-resistant coatings, functional nanocomposites, and hybrid electrode materials, which improve system durability, efficiency, and operational lifespan (Steffi et al., 2022a; Steffi et al., 2022b). Additionally, recent developments in electrochemical sensing platforms and pollutant detection systems provide critical insights into reaction mechanisms and system performance, enabling more precise control and optimization (Maheshwaran et al., 2022c; Singh et al., 2024c). These material level innovations, when combined with ML-driven optimization, enable the development of highly efficient, adaptive, and scalable bioenergy systems (Guo et al., 2020; Hossain et al., 2022; Jacob et al., 2021; Maheshwaran et al., 2022d; Peter et al., 2023; Riaz et al., 2024; Samuel et al., 2019; Samuel et al., 2022a; Samuel et al., 2022b; Singh et al., 2024a; Singh et al., 2024b; Zhang et al., 2023; Zhang et al., 2010). Overall, the integration of ML with TEA in microalgal biofuel production represents a transformative approach that extends beyond conventional economic analysis toward a comprehensive, data-driven, and sustainability-oriented framework. By incorporating multidisciplinary datasets including material properties, environmental parameters, process dynamics, and real-time monitoring data ML-assisted TEA enables improved prediction accuracy, reduced uncertainty, and enhanced decision-making. This holistic approach is essential for accelerating the commercialization of microalgal biofuel technologies while ensuring environmental compatibility, regulatory compliance, and long-term economic viability.

## 7 Challenges and future directions

Despite significant progress, several obstacles limit widespread AI deployment in bioenergy systems. High computational demands for training ML models can strain available infrastructure. Variability in biomass feedstock quality introduces uncertainty, affecting predictive reliability and process optimization. Scalability also remains challenging, as integrating AI into large-scale facilities requires substantial capital investment and technical expertise. Addressing these issues necessitates coordinated research initiatives, supportive policy frameworks, investments in digital infrastructure. Integrating AI with life cycle assessment (LCA) methodologies offers a comprehensive approach to evaluating environmental and economic impacts. In summary, AI integration within bioenergy systems offers transformative potential for process enhancement sustainability improvement, and mitigation of environmental challenges. By leveraging AI-enabled innovations, biowaste-to-bioenergy systems can substantially advance circular bioeconomy objectives and contribute to global energy and climate targets.

## Data Availability

The original contributions presented in the study are included in the article/supplementary material, further inquiries can be directed to the corresponding authors.
