# Supplementary material for: Artificial intelligence driven microalgae based green fabrication and bioenergy systems for sustainable energy materials and biowaste valorization
Source: Front Chem. 2026 Jun 24;14:1858141. doi: 10.3389/fchem.2026.1858141 (PMC13342021; doi:10.3389/fchem.2026.1858141)

## Slide 1
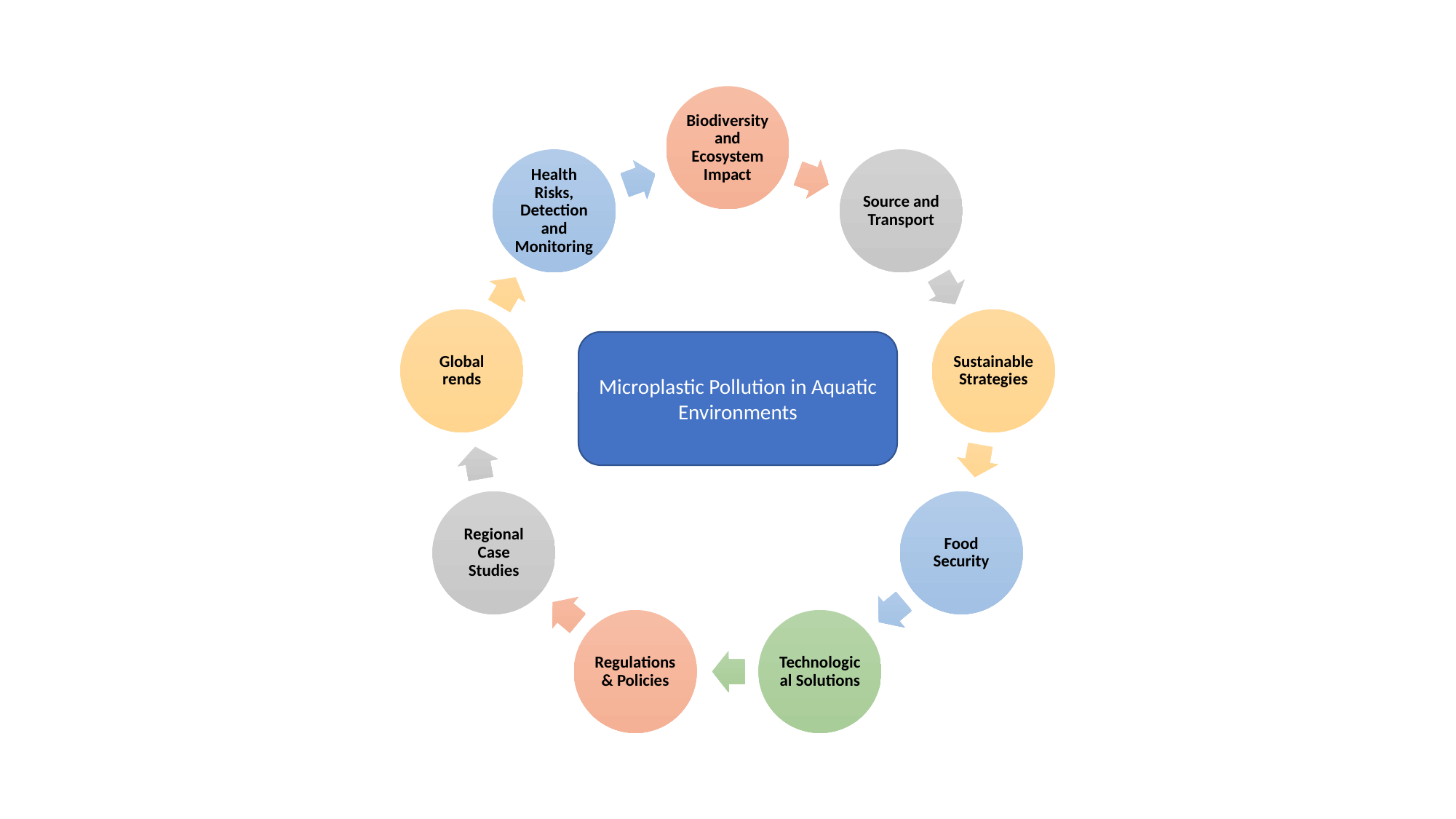

Microplastic Pollution in Aquatic Environments

## Slide 2
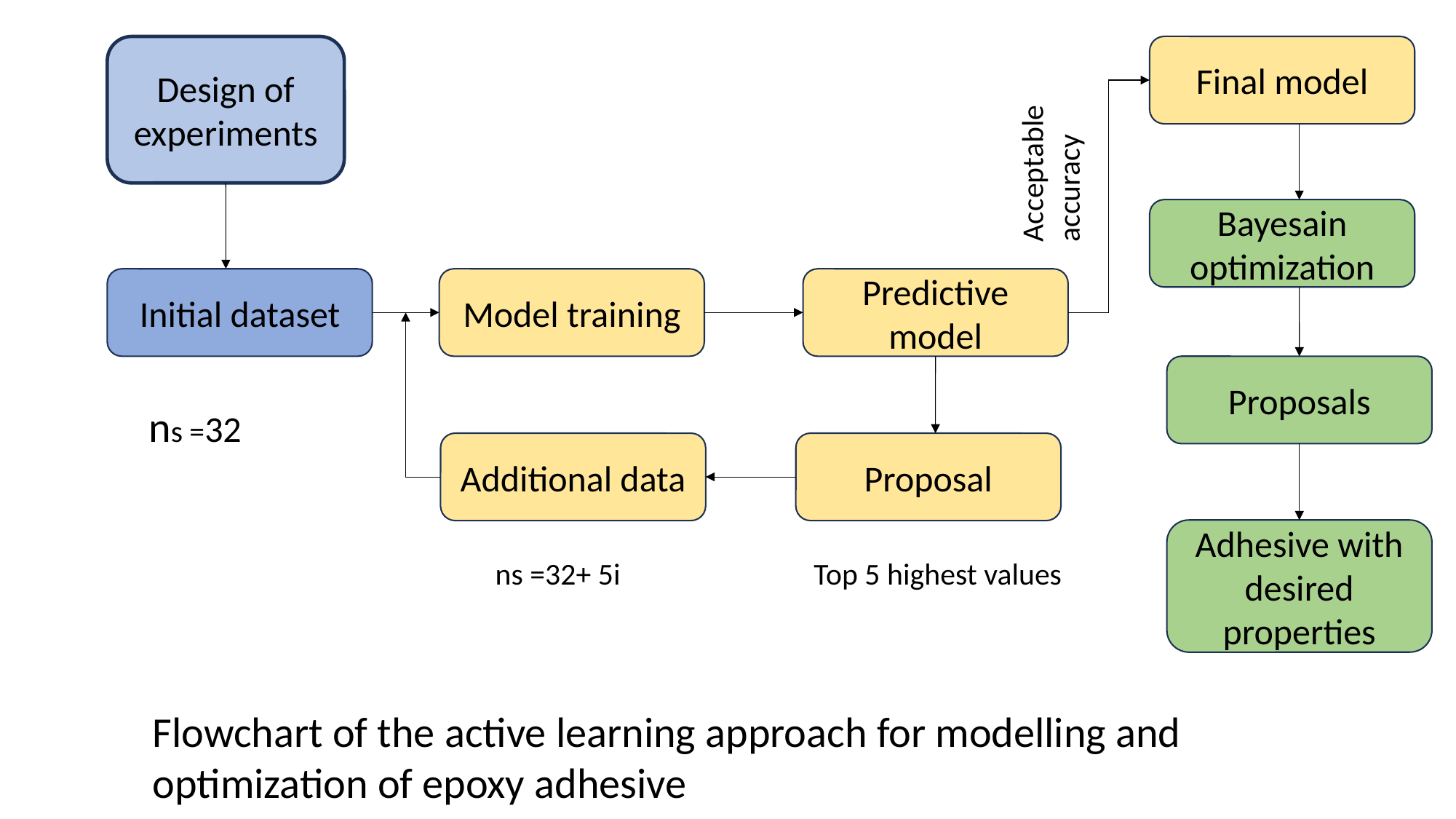

Design of experiments
Final model
Acceptable accuracy
Bayesain optimization
Initial dataset
Model training
Predictive model
Proposals
ns =32
Additional data
Proposal
Adhesive with desired properties
ns =32+ 5i
Top 5 highest values
Flowchart of the active learning approach for modelling and optimization of epoxy adhesive

## Slide 3
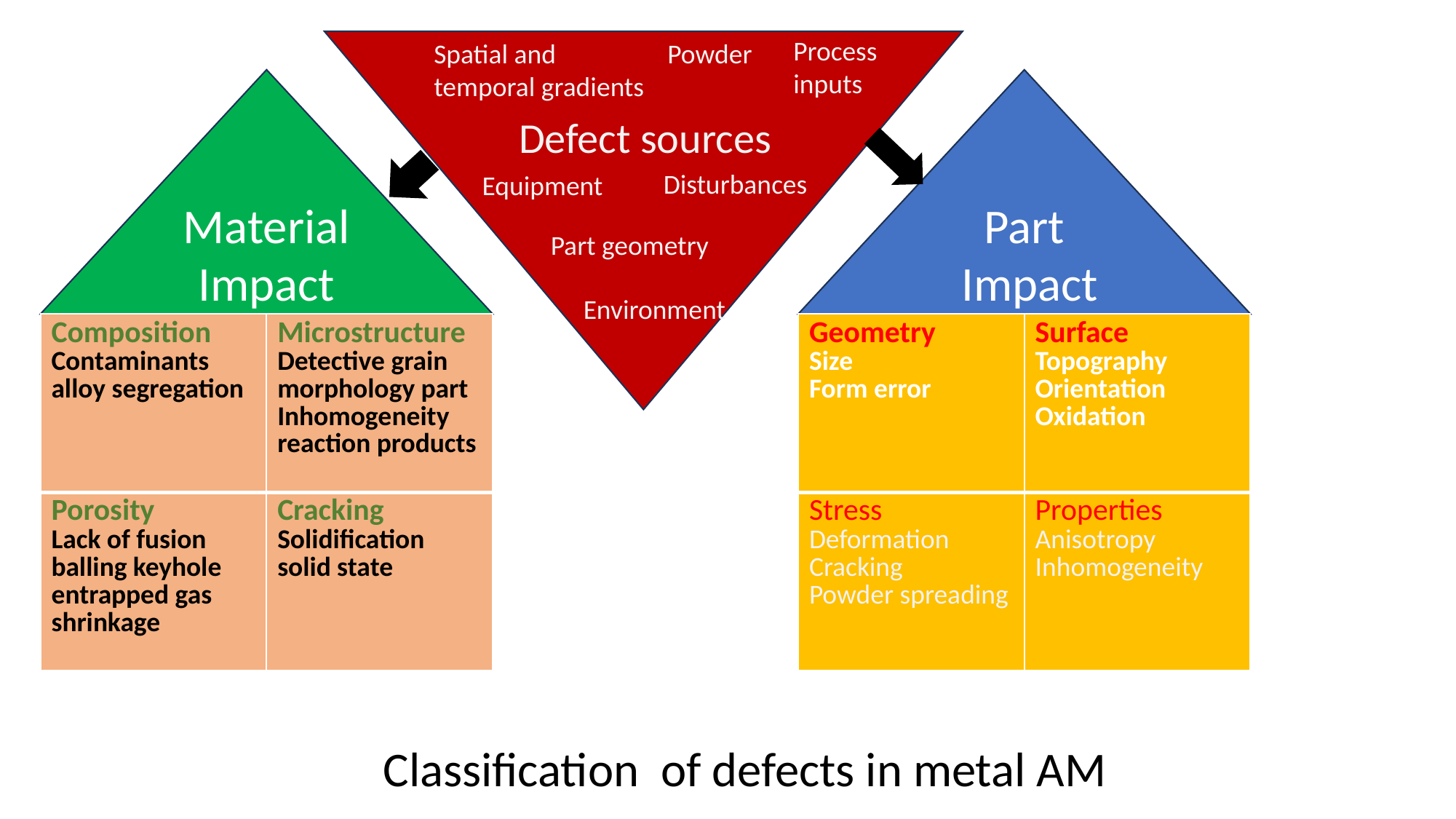

Process inputs
Spatial and temporal gradients
Powder
Material Impact
Part
 Impact
Defect sources
Disturbances
Equipment
Part geometry
Environment
| Composition Contaminants alloy segregation | Microstructure Detective grain morphology part Inhomogeneity reaction products |
| --- | --- |
| Porosity Lack of fusion balling keyhole entrapped gas shrinkage | Cracking Solidification solid state |
| Geometry Size Form error | Surface Topography Orientation Oxidation |
| --- | --- |
| Stress Deformation Cracking Powder spreading | Properties Anisotropy Inhomogeneity |
Classification of defects in metal AM

## Slide 4
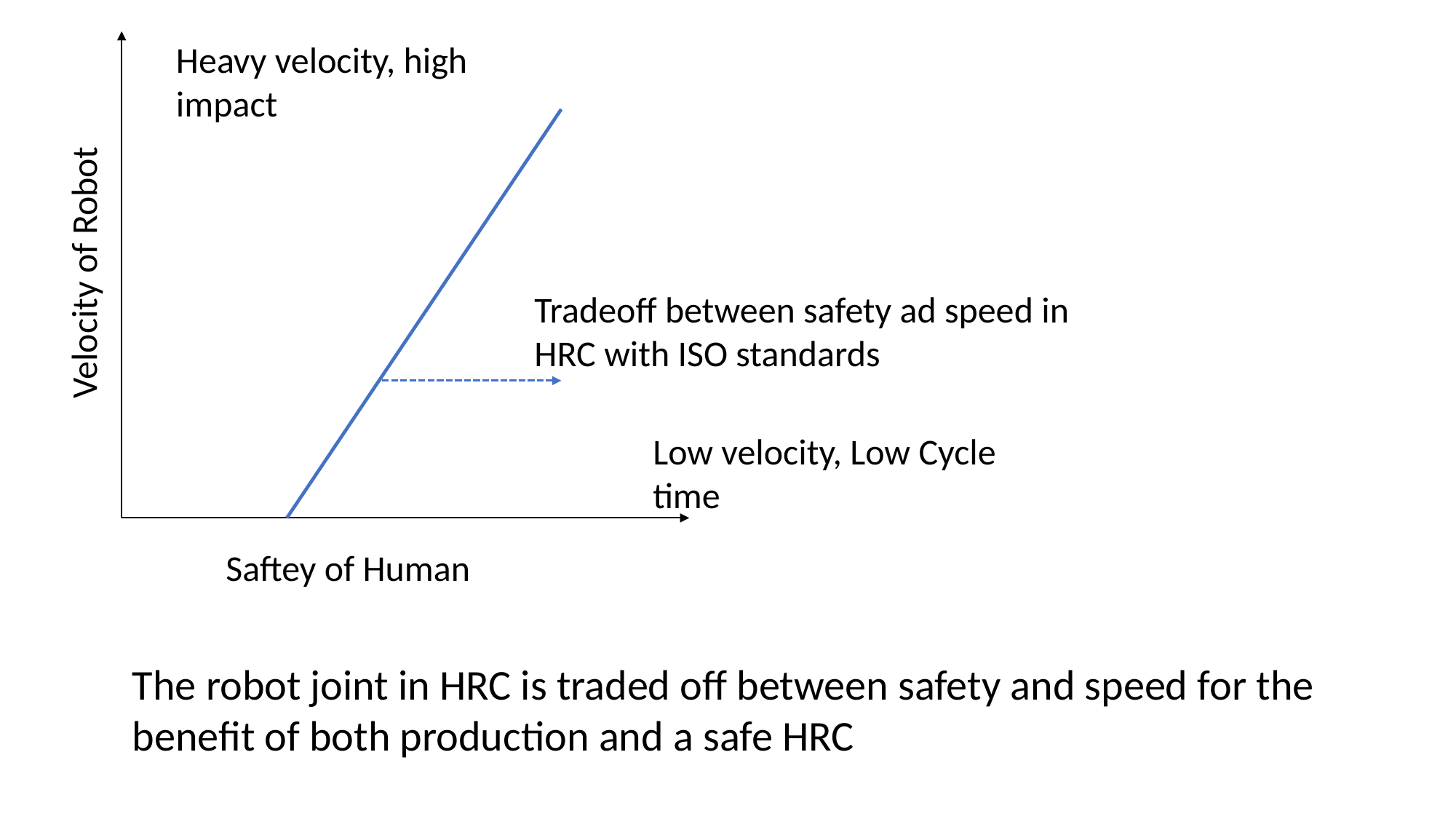

Heavy velocity, high impact
Velocity of Robot
Tradeoff between safety ad speed in HRC with ISO standards
Low velocity, Low Cycle time
Saftey of Human
The robot joint in HRC is traded off between safety and speed for the benefit of both production and a safe HRC

## Slide 5
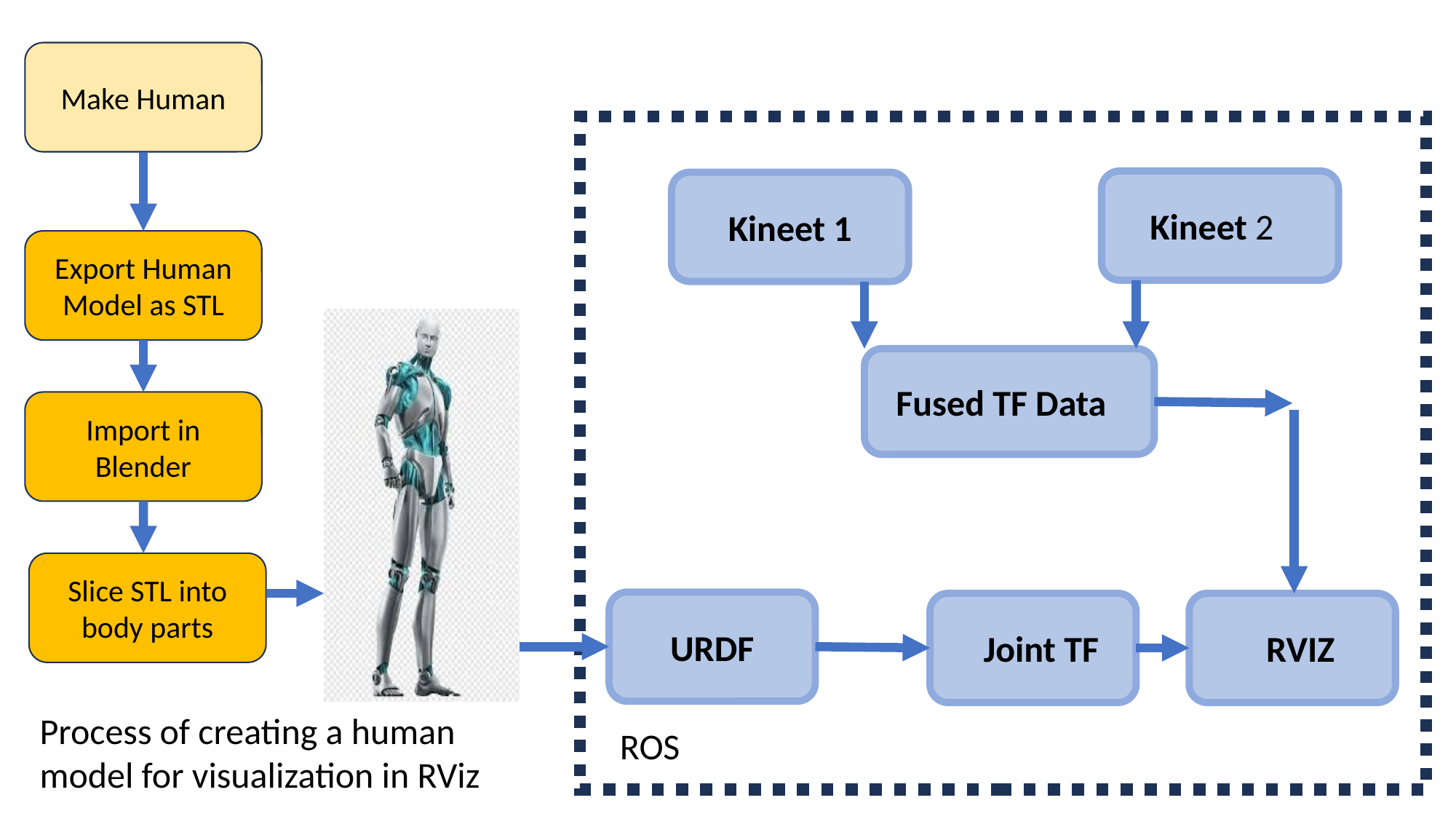

Make Human
Kineet 2
Kineet 1
Export Human Model as STL
Fused TF Data
Import in Blender
Slice STL into body parts
 URDF
 Joint TF
 RVIZ
Process of creating a human model for visualization in RViz
ROS

## Slide 6
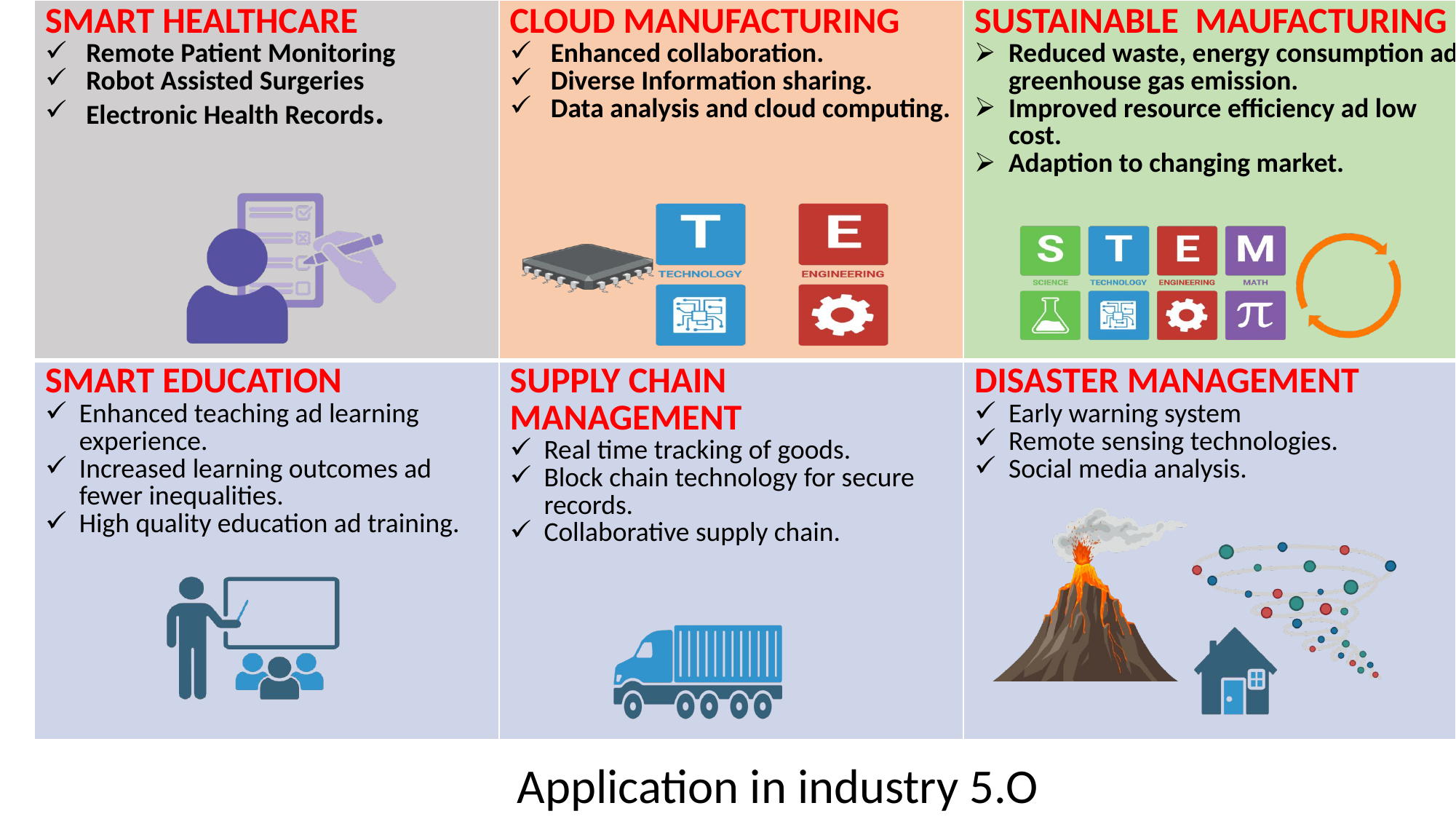

| SMART HEALTHCARE Remote Patient Monitoring Robot Assisted Surgeries Electronic Health Records. | CLOUD MANUFACTURING Enhanced collaboration. Diverse Information sharing. Data analysis and cloud computing. | SUSTAINABLE MAUFACTURING Reduced waste, energy consumption ad greenhouse gas emission. Improved resource efficiency ad low cost. Adaption to changing market. |
| --- | --- | --- |
| SMART EDUCATION Enhanced teaching ad learning experience. Increased learning outcomes ad fewer inequalities. High quality education ad training. | SUPPLY CHAIN MANAGEMENT Real time tracking of goods. Block chain technology for secure records. Collaborative supply chain. | DISASTER MANAGEMENT Early warning system Remote sensing technologies. Social media analysis. |
 Application in industry 5.O

## Slide 7
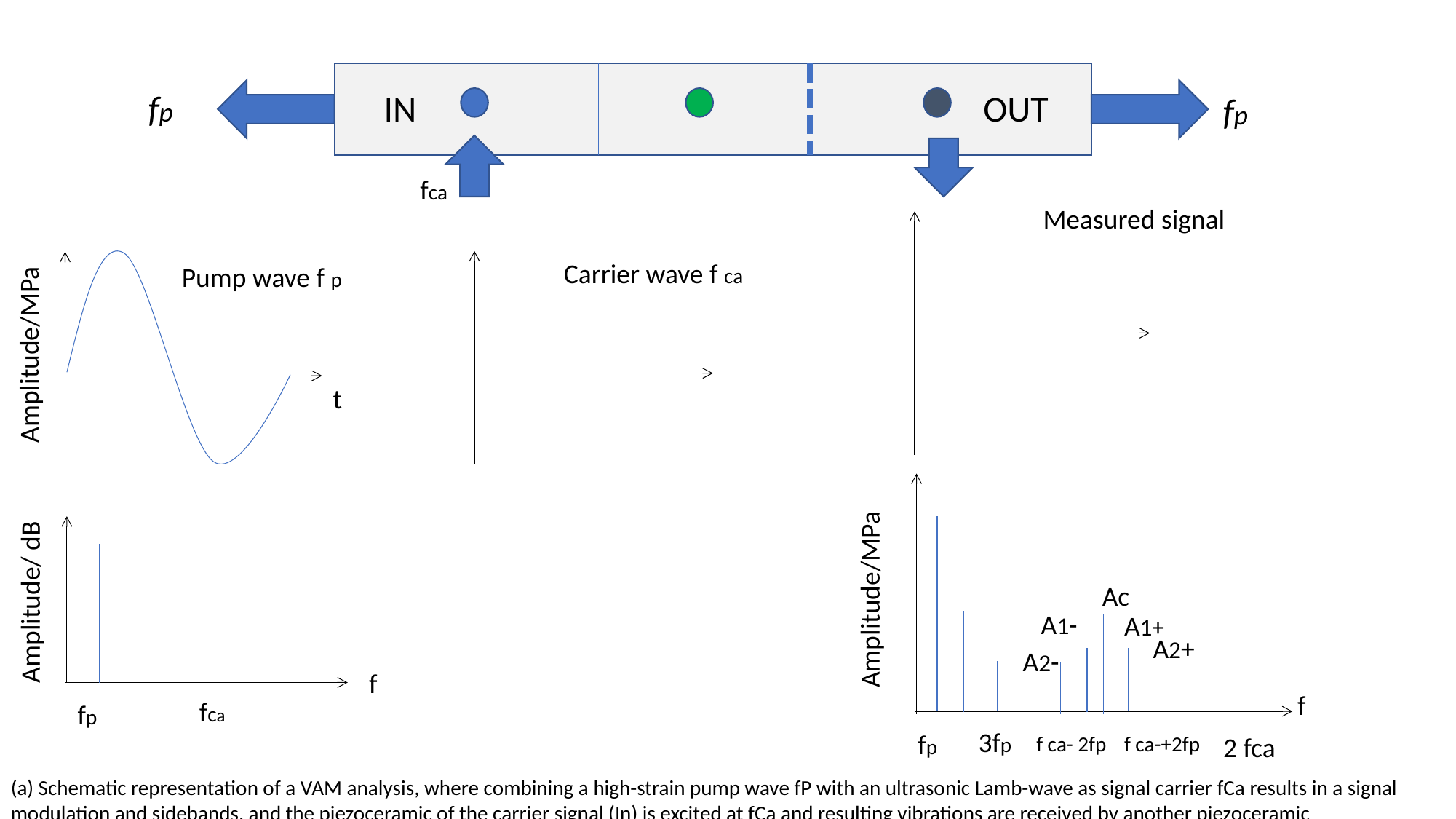

fp
IN
OUT
fp
fca
Measured signal
Carrier wave f ca
Pump wave f p
Amplitude/MPa
t
Ac
Amplitude/MPa
Amplitude/ dB
A1-
A1+
A2+
A2-
f
f
fca
fp
3fp
fp
f ca- 2fp
f ca-+2fp
2 fca
(a) Schematic representation of a VAM analysis, where combining a high-strain pump wave fP with an ultrasonic Lamb-wave as signal carrier fCa results in a signal modulation and sidebands, and the piezoceramic of the carrier signal (In) is excited at fCa and resulting vibrations are received by another piezoceramic
actuator (Out)

## Slide 8
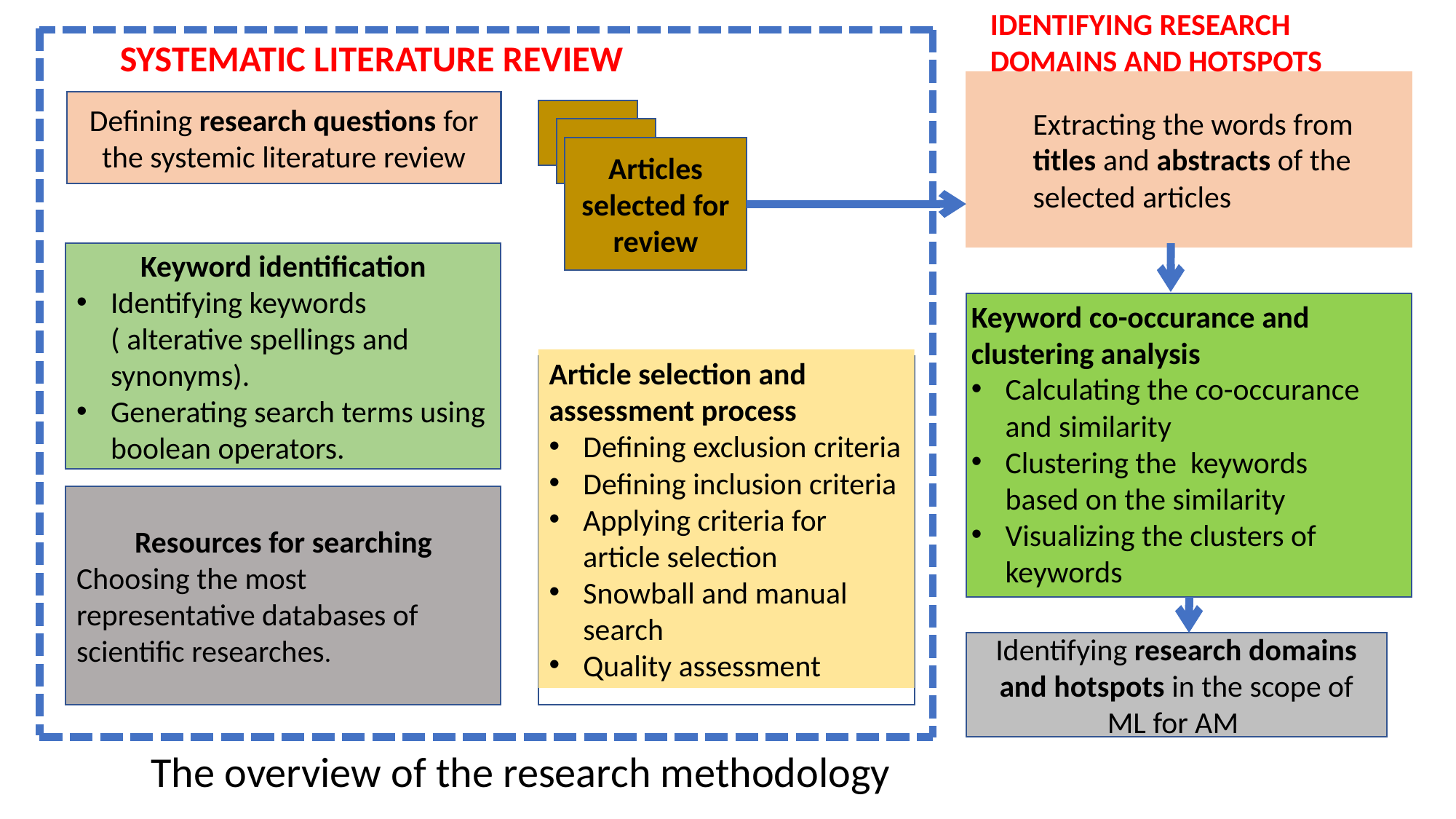

IDENTIFYING RESEARCH DOMAINS AND HOTSPOTS
SYSTEMATIC LITERATURE REVIEW
Defining research questions for the systemic literature review
Extracting the words from titles and abstracts of the selected articles
Articles selected for review
Keyword identification
Identifying keywords ( alterative spellings and synonyms).
Generating search terms using boolean operators.
Keyword co-occurance and clustering analysis
Calculating the co-occurance and similarity
Clustering the keywords based on the similarity
Visualizing the clusters of keywords
Article selection and assessment process
Defining exclusion criteria
Defining inclusion criteria
Applying criteria for article selection
Snowball and manual search
Quality assessment
Resources for searching
Choosing the most representative databases of scientific researches.
Identifying research domains and hotspots in the scope of ML for AM
The overview of the research methodology

## Slide 9
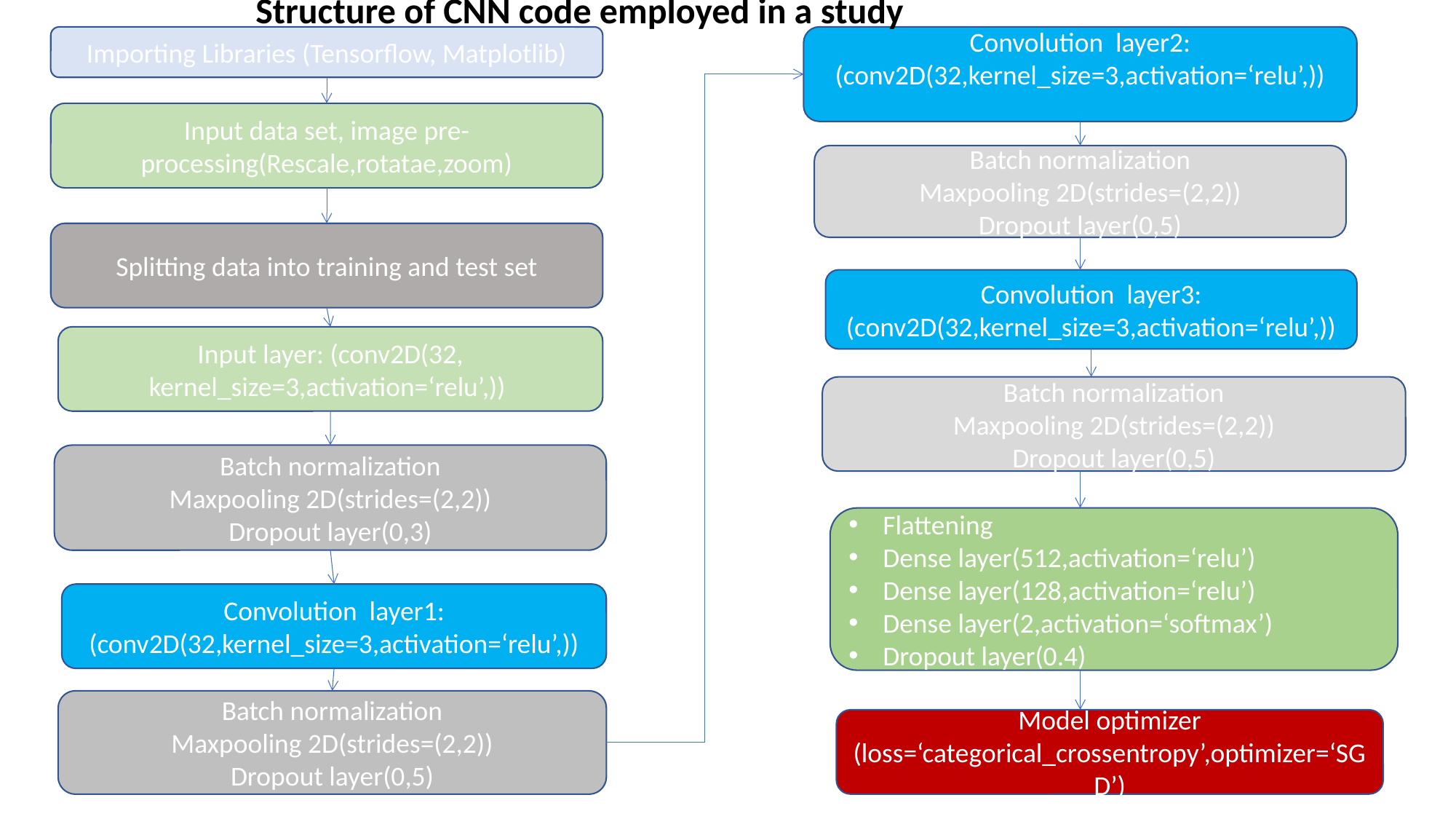

Structure of CNN code employed in a study
Importing Libraries (Tensorflow, Matplotlib)
Convolution layer2: (conv2D(32,kernel_size=3,activation=‘relu’,))
Input data set, image pre-processing(Rescale,rotatae,zoom)
Batch normalization
Maxpooling 2D(strides=(2,2))
Dropout layer(0,5)
Splitting data into training and test set
Convolution layer3: (conv2D(32,kernel_size=3,activation=‘relu’,))
Input layer: (conv2D(32, kernel_size=3,activation=‘relu’,))
Batch normalization
Maxpooling 2D(strides=(2,2))
Dropout layer(0,5)
Batch normalization
Maxpooling 2D(strides=(2,2))
Dropout layer(0,3)
Flattening
Dense layer(512,activation=‘relu’)
Dense layer(128,activation=‘relu’)
Dense layer(2,activation=‘softmax’)
Dropout layer(0.4)
Convolution layer1: (conv2D(32,kernel_size=3,activation=‘relu’,))
Batch normalization
Maxpooling 2D(strides=(2,2))
Dropout layer(0,5)
Model optimizer
(loss=‘categorical_crossentropy’,optimizer=‘SGD’)

## Slide 10
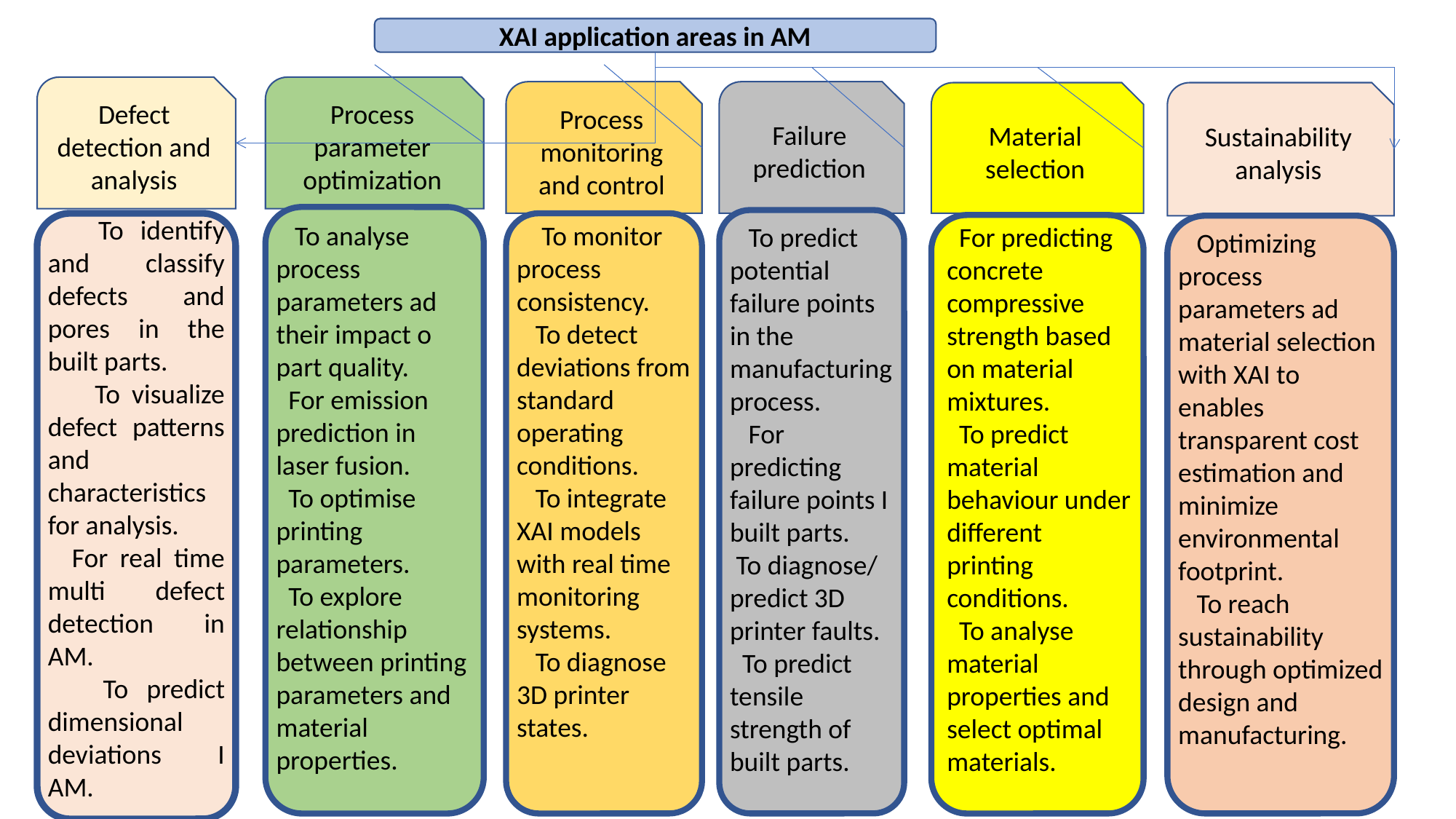

XAI application areas in AM
Defect detection and analysis
Process parameter optimization
Process monitoring and control
Failure prediction
Material selection
Sustainability analysis
 To identify and classify defects and pores in the built parts.
 To visualize defect patterns and characteristics for analysis.
 For real time multi defect detection in AM.
 To predict dimensional deviations I AM.
 To analyse process parameters ad their impact o part quality.
 For emission prediction in laser fusion.
 To optimise printing parameters.
 To explore relationship between printing parameters and material properties.
 To monitor process consistency.
 To detect deviations from standard operating conditions.
 To integrate XAI models with real time monitoring systems.
 To diagnose 3D printer states.
 To predict potential failure points in the manufacturing process.
 For predicting failure points I built parts.
 To diagnose/ predict 3D printer faults.
 To predict tensile strength of built parts.
 For predicting concrete compressive strength based on material mixtures.
 To predict material behaviour under different printing conditions.
 To analyse material properties and select optimal materials.
 Optimizing process parameters ad material selection with XAI to enables transparent cost estimation and minimize environmental footprint.
 To reach sustainability through optimized design and manufacturing.

## Slide 11
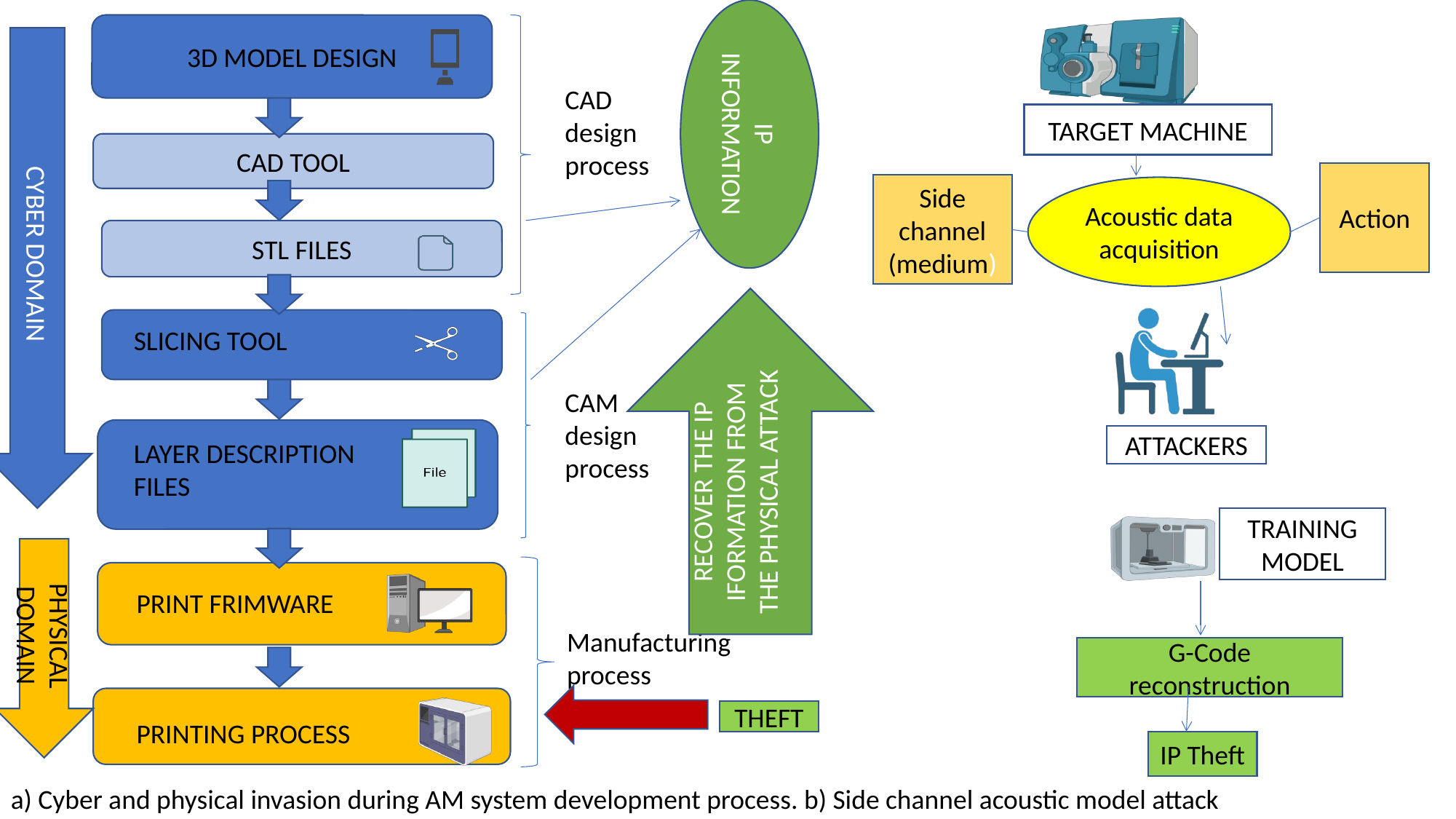

3D MODEL DESIGN
IP INFORMATION
CAD design process
TARGET MACHINE
CAD TOOL
Action
Side channel (medium)
Acoustic data acquisition
CYBER DOMAIN
STL FILES
SLICING TOOL
RECOVER THE IP IFORMATION FROM THE PHYSICAL ATTACK
CAM design process
ATTACKERS
LAYER DESCRIPTION FILES
TRAINING MODEL
PRINT FRIMWARE
PHYSICAL DOMAIN
Manufacturing
process
G-Code reconstruction
THEFT
PRINTING PROCESS
IP Theft
a) Cyber and physical invasion during AM system development process. b) Side channel acoustic model attack

## Slide 12
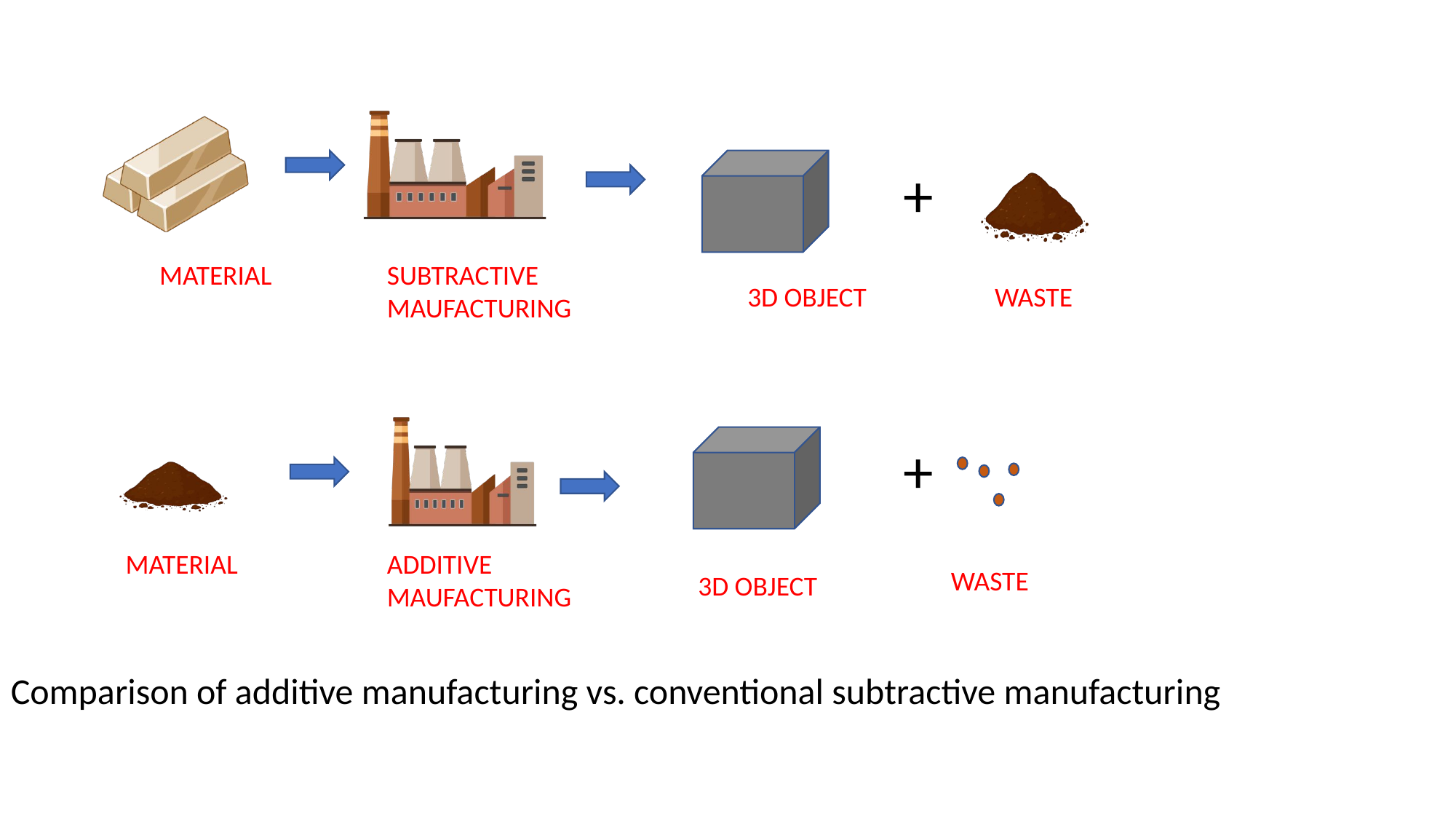

+
MATERIAL
SUBTRACTIVE MAUFACTURING
3D OBJECT
WASTE
+
MATERIAL
ADDITIVE MAUFACTURING
WASTE
3D OBJECT
Comparison of additive manufacturing vs. conventional subtractive manufacturing

## Slide 13
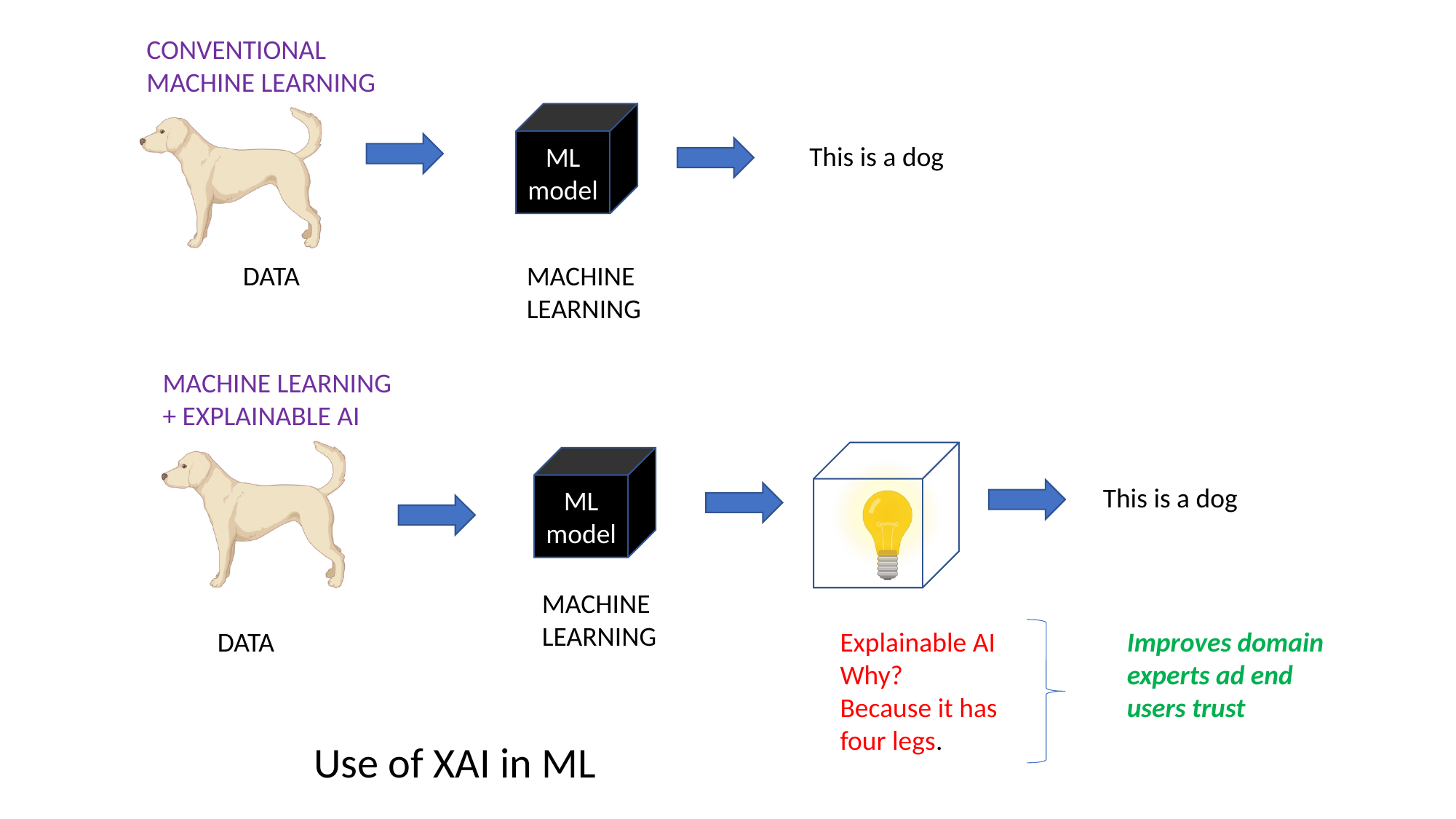

CONVENTIONAL MACHINE LEARNING
ML model
This is a dog
DATA
MACHINE LEARNING
MACHINE LEARNING
+ EXPLAINABLE AI
ML model
This is a dog
MACHINE LEARNING
DATA
Explainable AI
Why?
Because it has four legs.
Improves domain experts ad end users trust
Use of XAI in ML

## Slide 14
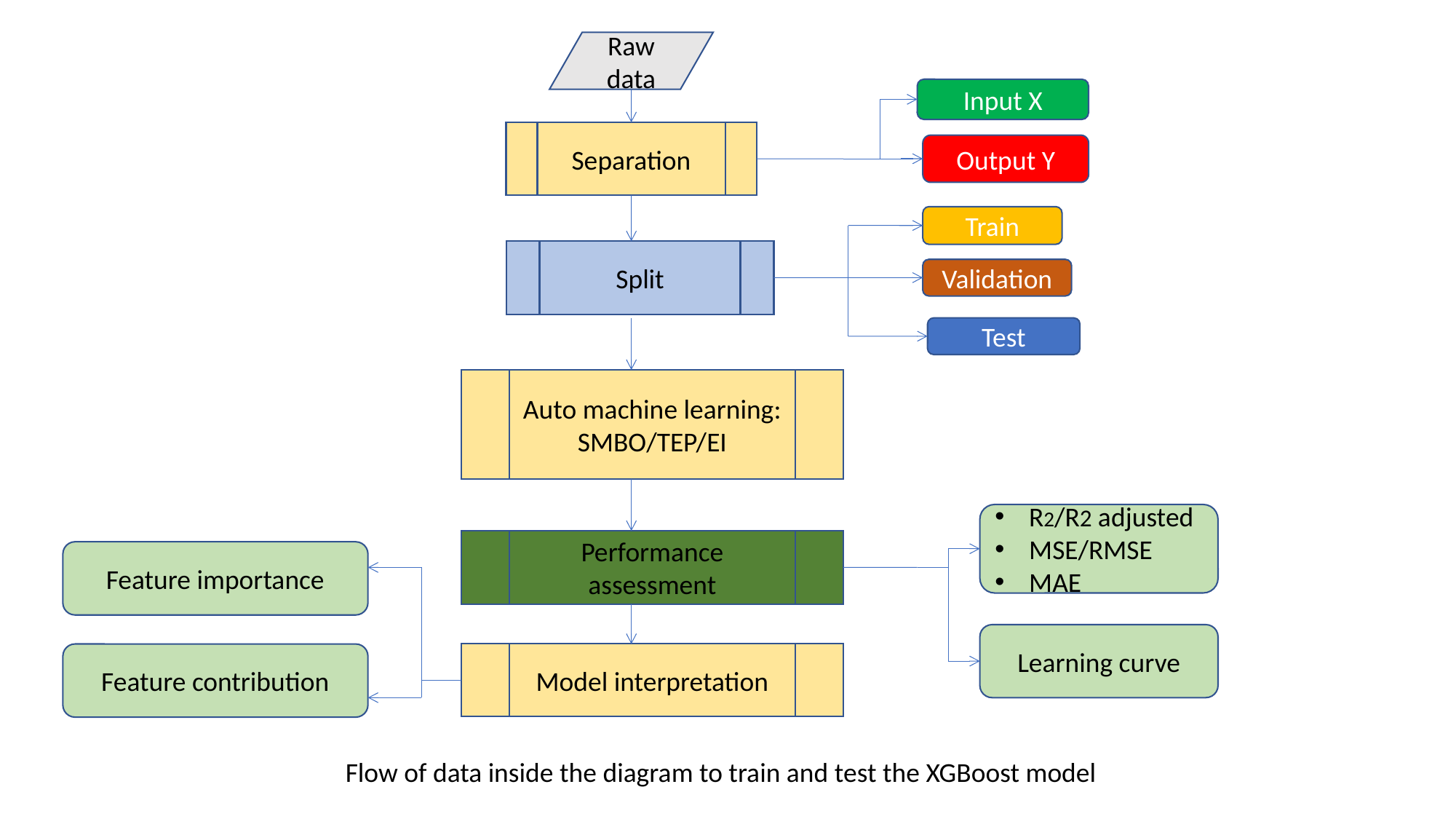

Raw data
Input X
Separation
Output Y
Train
Split
Validation
Test
Auto machine learning: SMBO/TEP/EI
R2/R2 adjusted
MSE/RMSE
MAE
Performance assessment
Feature importance
Learning curve
Model interpretation
Feature contribution
Flow of data inside the diagram to train and test the XGBoost model

## Slide 15
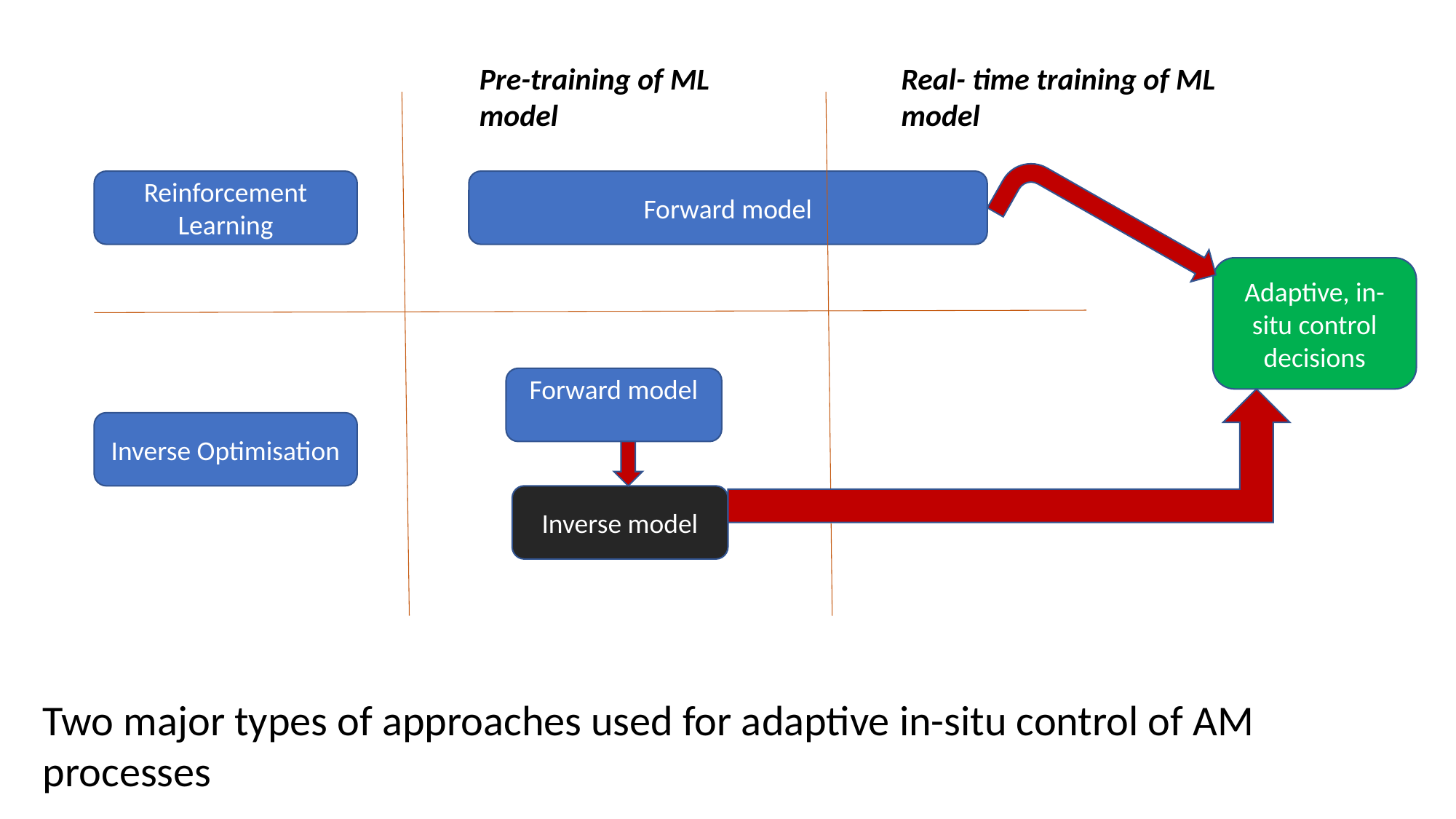

Pre-training of ML model
Real- time training of ML model
Reinforcement Learning
Forward model
Adaptive, in-situ control decisions
Forward model
Inverse Optimisation
Inverse model
Two major types of approaches used for adaptive in-situ control of AM processes

## Slide 16
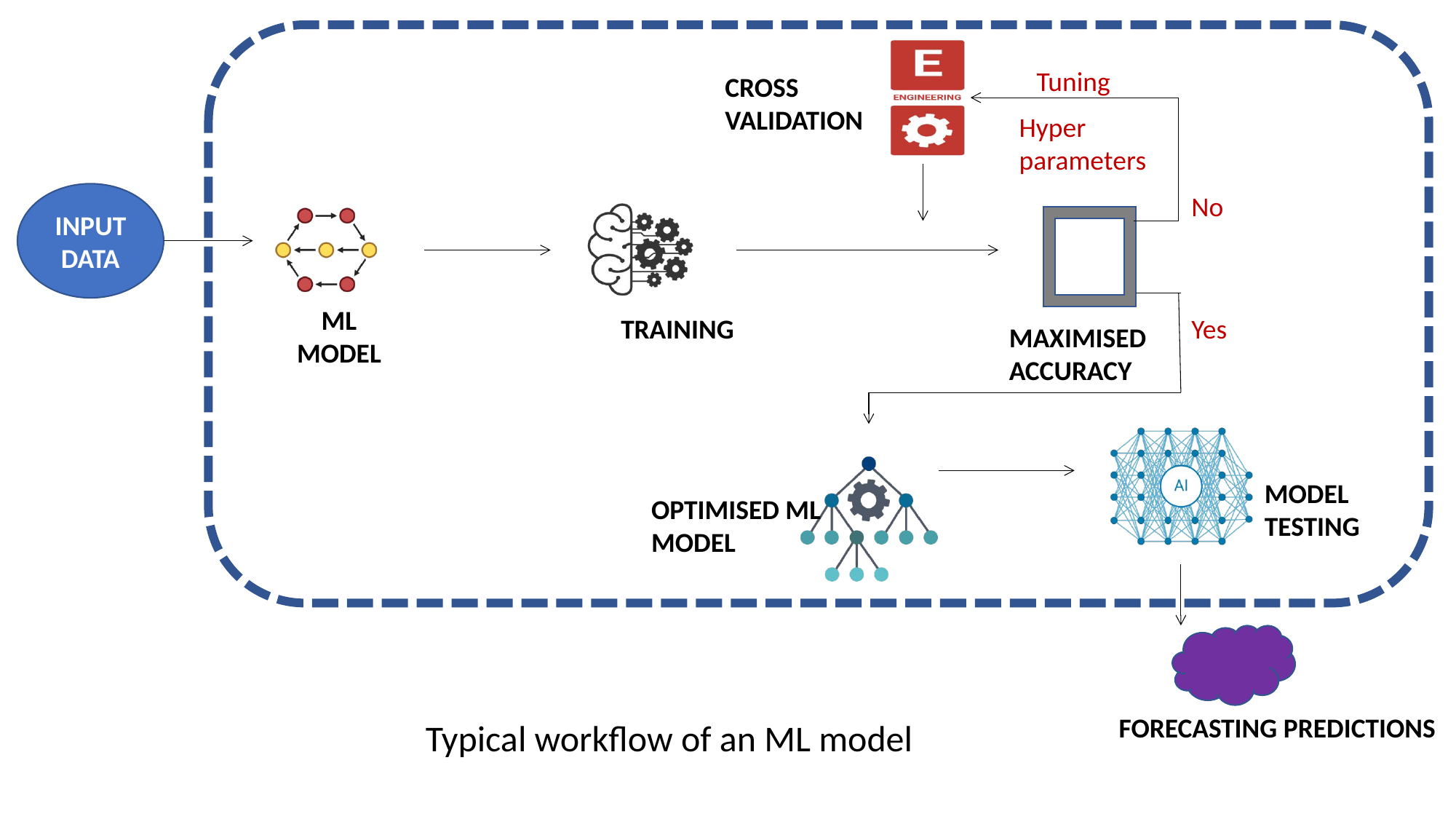

Tuning
CROSS VALIDATION
Hyper parameters
INPUT DATA
No
ML MODEL
TRAINING
Yes
MAXIMISED ACCURACY
MODEL TESTING
OPTIMISED ML MODEL
FORECASTING PREDICTIONS
Typical workflow of an ML model

## Slide 17
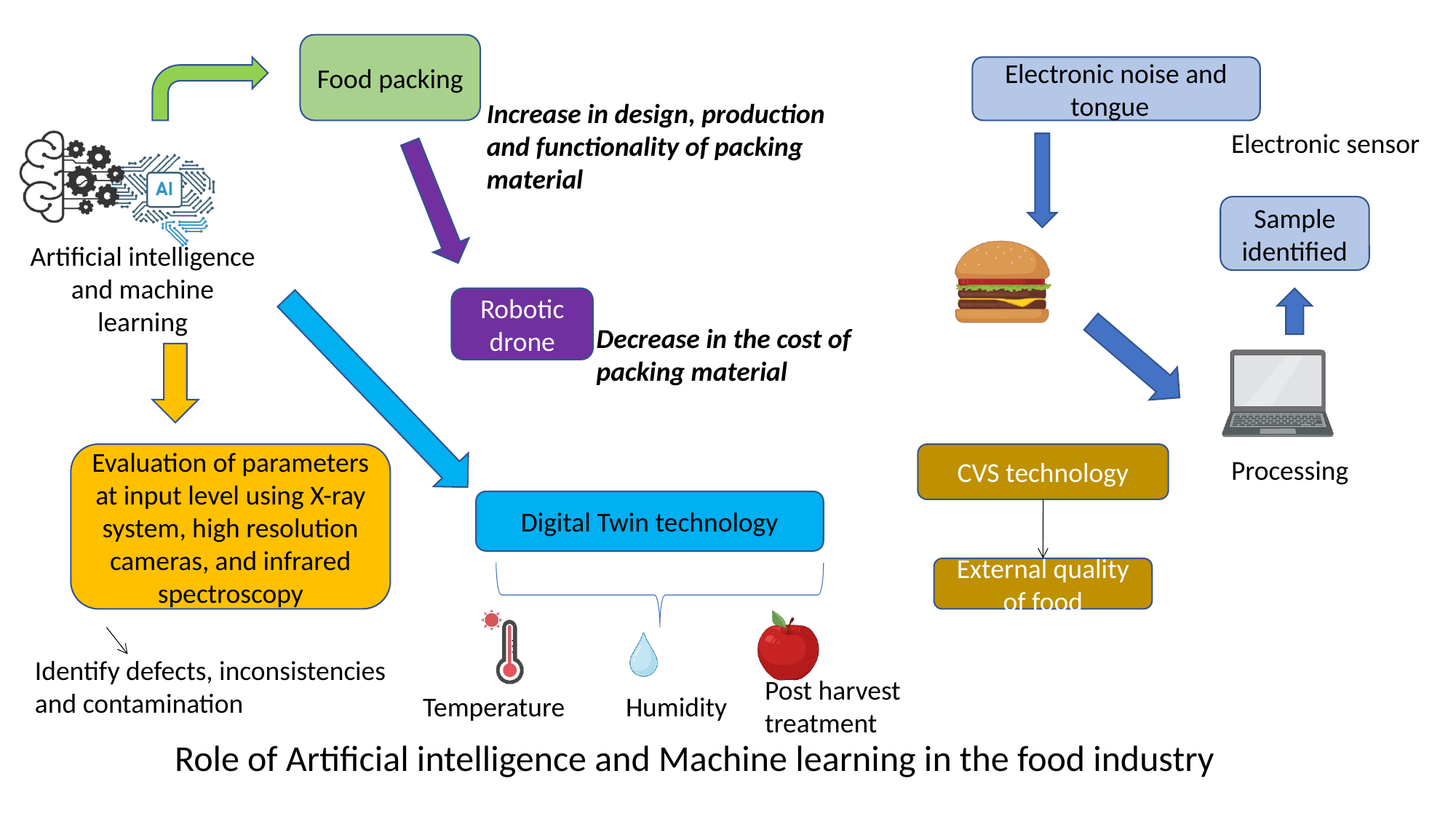

Food packing
Electronic noise and tongue
Increase in design, production and functionality of packing material
Electronic sensor
Sample identified
Artificial intelligence and machine learning
Robotic drone
Decrease in the cost of packing material
Evaluation of parameters at input level using X-ray system, high resolution cameras, and infrared spectroscopy
CVS technology
Processing
Digital Twin technology
External quality of food
Identify defects, inconsistencies and contamination
Post harvest treatment
Temperature
Humidity
Role of Artificial intelligence and Machine learning in the food industry

## Slide 18
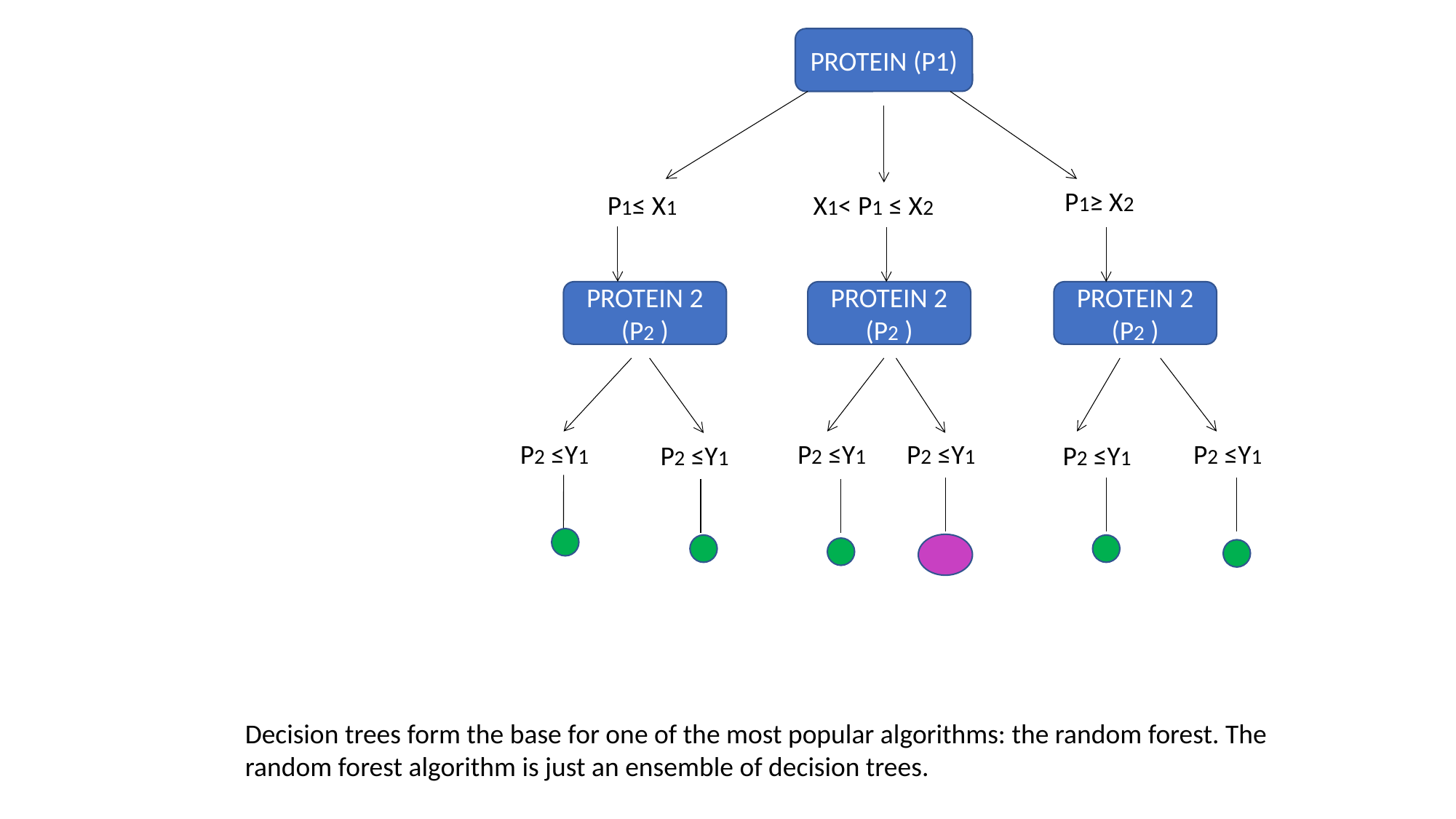

PROTEIN (P1)
P1≥ X2
P1≤ X1
X1< P1 ≤ X2
PROTEIN 2 (P2 )
PROTEIN 2 (P2 )
PROTEIN 2 (P2 )
P2 ≤Y1
P2 ≤Y1
P2 ≤Y1
P2 ≤Y1
P2 ≤Y1
P2 ≤Y1
Decision trees form the base for one of the most popular algorithms: the random forest. The random forest algorithm is just an ensemble of decision trees.

## Slide 19
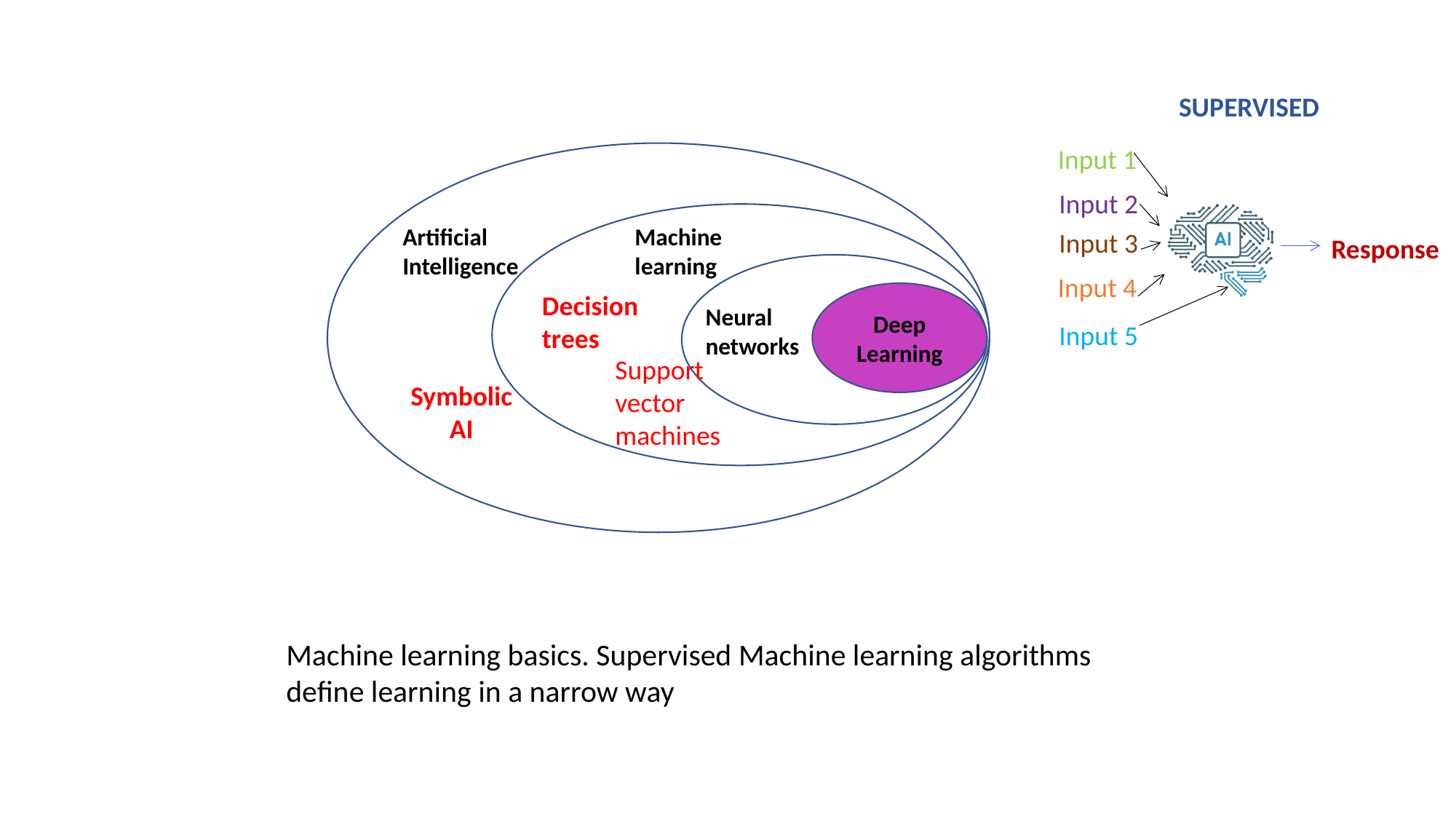

SUPERVISED
Input 1
Input 2
Artificial Intelligence
Machine learning
Input 3
Response
Input 4
Decision trees
Deep Learning
Neural networks
Input 5
Support vector machines
Symbolic AI
Machine learning basics. Supervised Machine learning algorithms
define learning in a narrow way

## Slide 20
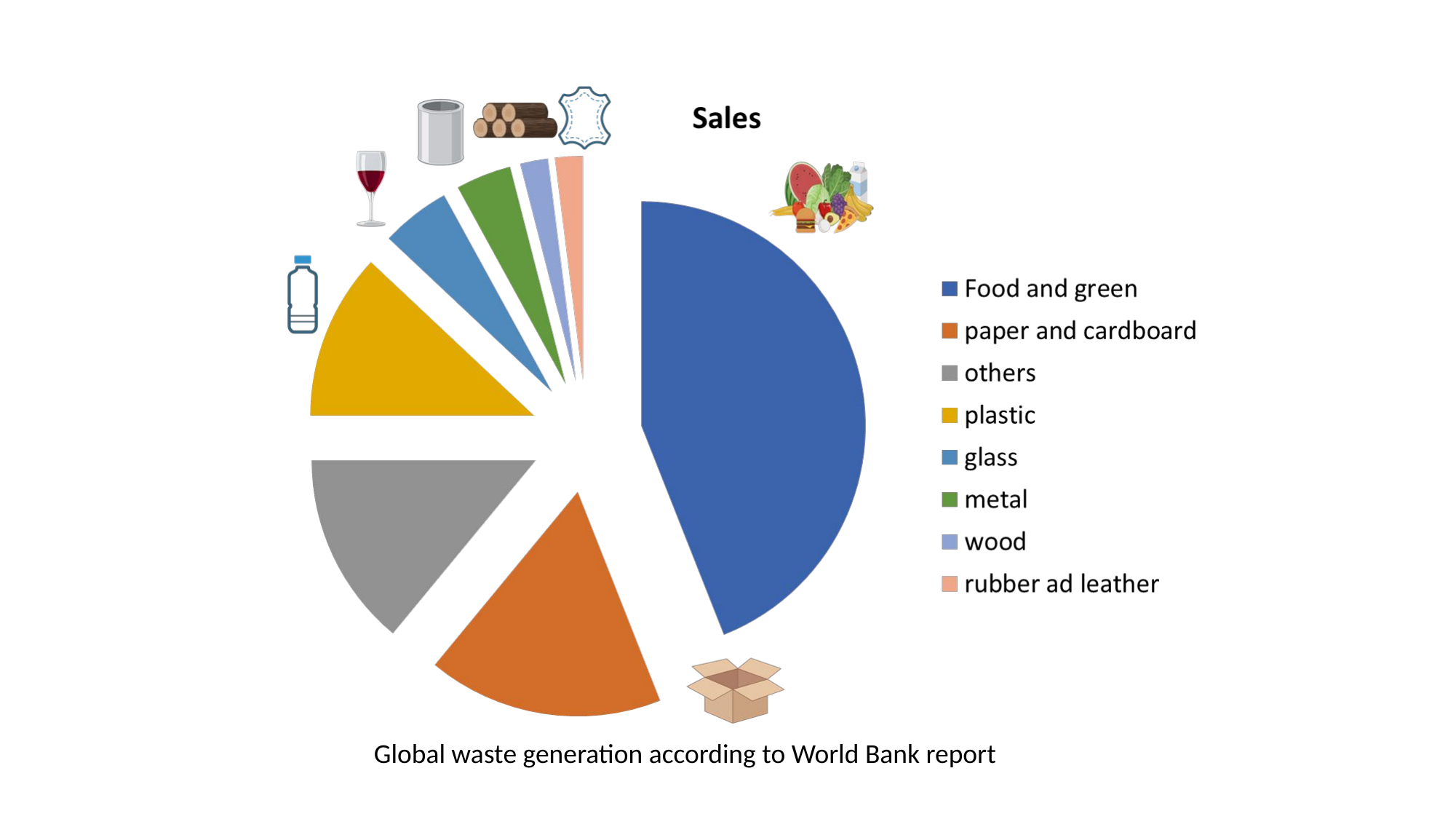

Global waste generation according to World Bank report

## Slide 21
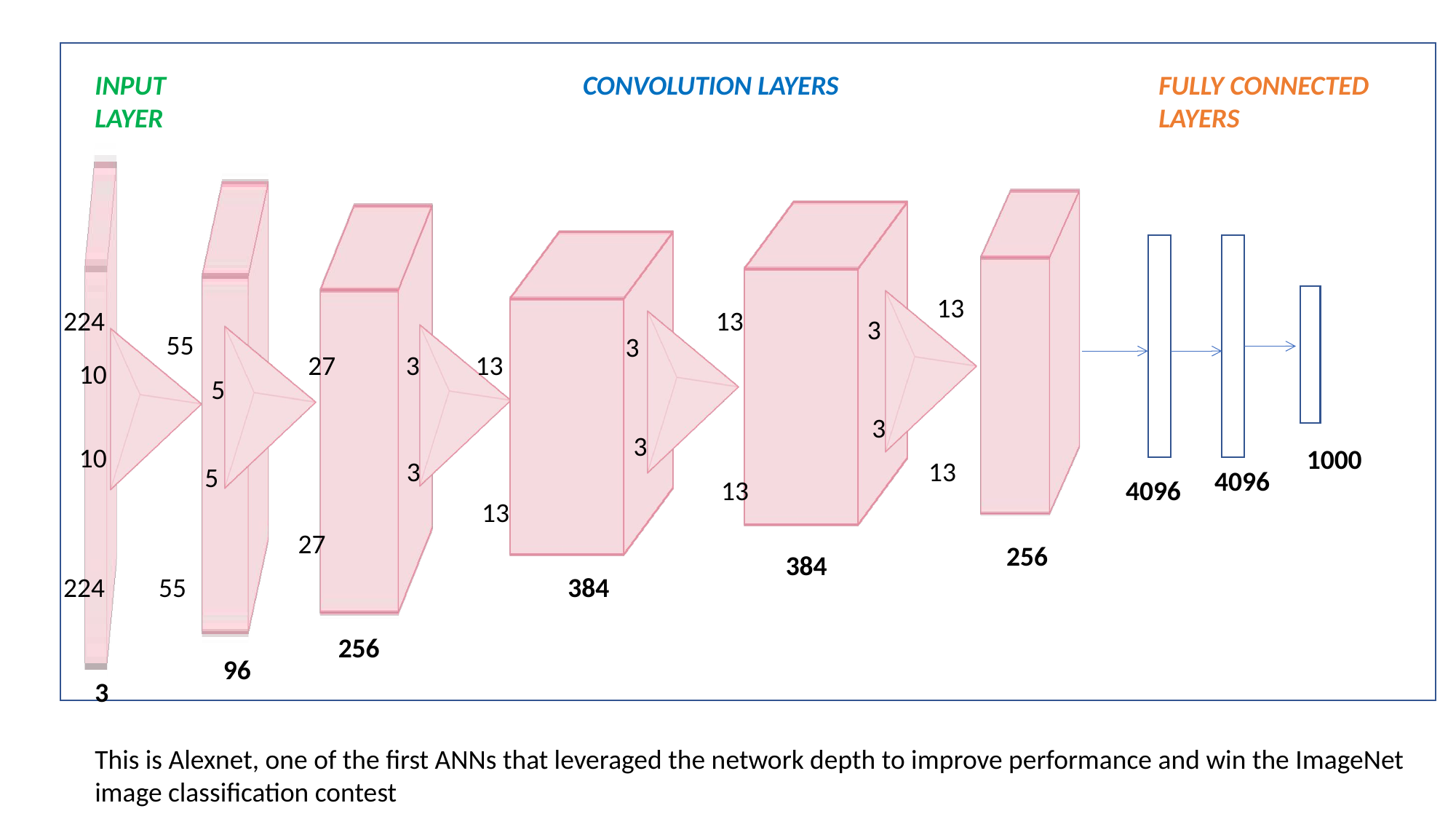

INPUT LAYER
CONVOLUTION LAYERS
FULLY CONNECTED LAYERS
13
224
13
3
55
3
27
3
13
10
5
3
3
10
1000
3
13
5
4096
13
4096
13
27
256
384
224
55
384
256
96
3
This is Alexnet, one of the first ANNs that leveraged the network depth to improve performance and win the ImageNet image classification contest

## Slide 22
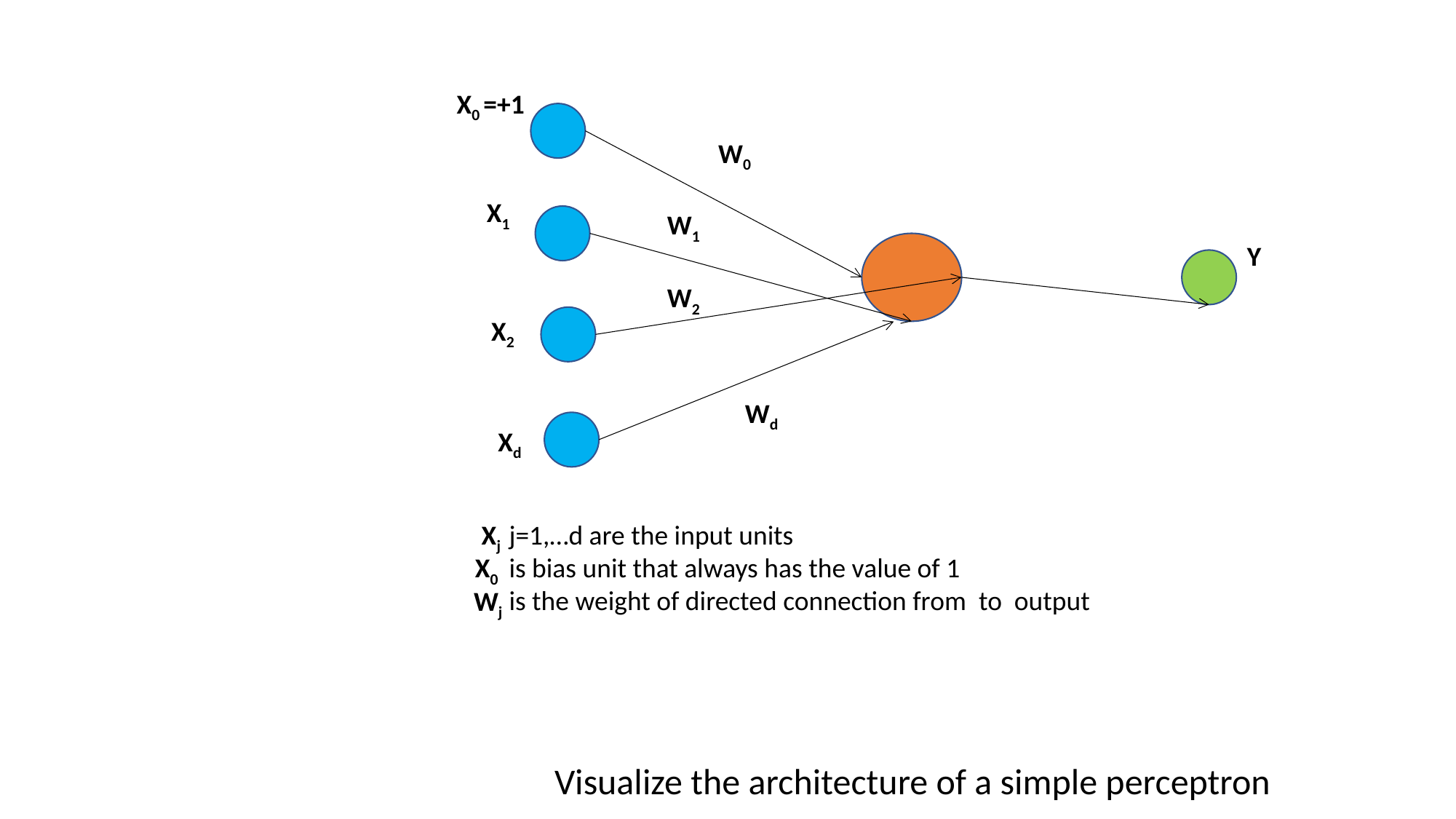

X0 =+1
W0
X1
W1
Y
W2
X2
Wd
Xd
Xj
j=1,…d are the input units
is bias unit that always has the value of 1
is the weight of directed connection from to output
X0
Wj
Visualize the architecture of a simple perceptron

## Slide 23
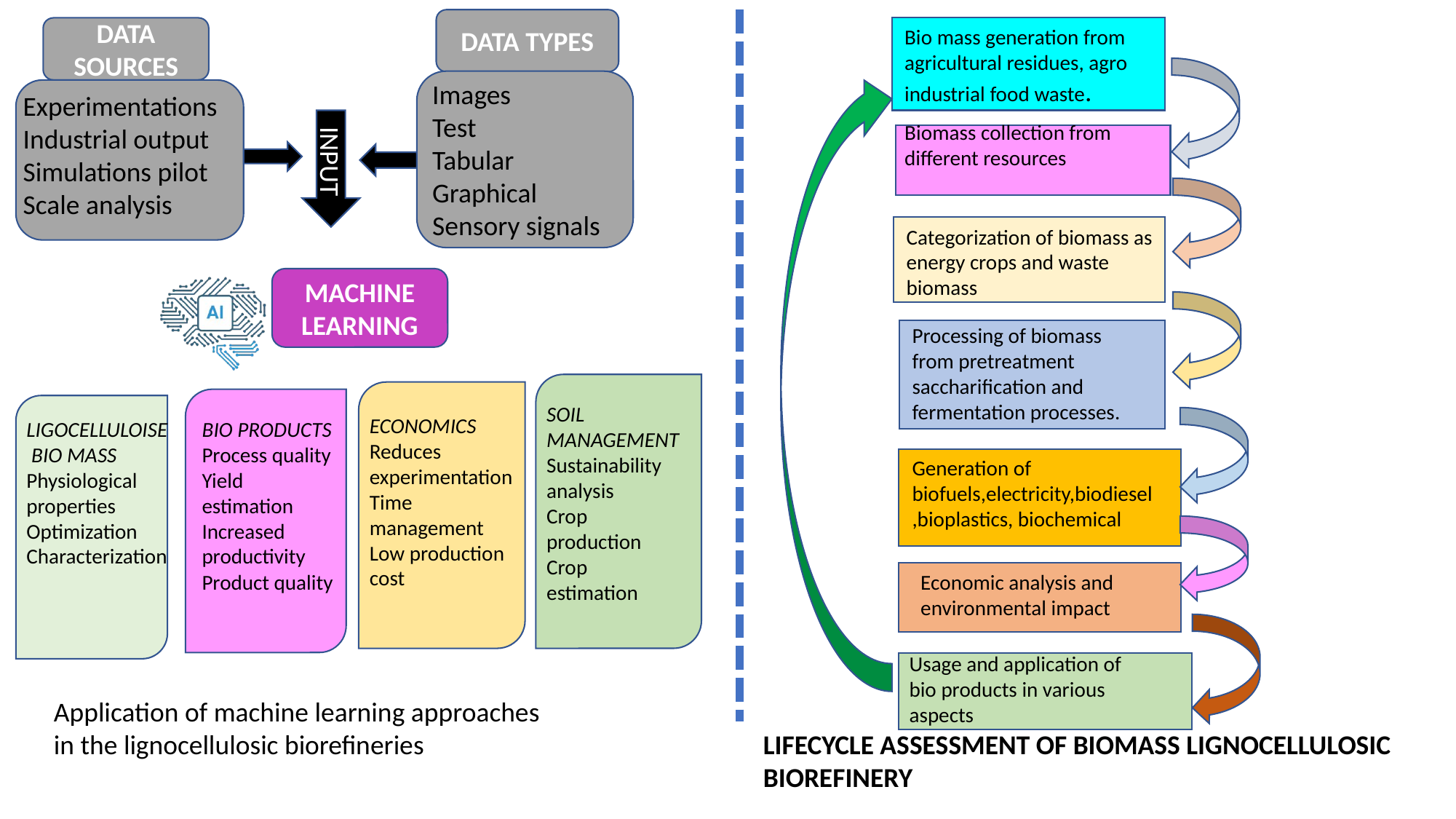

DATA TYPES
DATA SOURCES
Bio mass generation from agricultural residues, agro industrial food waste.
Images
Test
Tabular
Graphical
Sensory signals
Experimentations
Industrial output Simulations pilot Scale analysis
Biomass collection from different resources
INPUT
Categorization of biomass as energy crops and waste biomass
MACHINE LEARNING
Processing of biomass from pretreatment saccharification and fermentation processes.
SOIL MANAGEMENT
Sustainability analysis
Crop production
Crop estimation
ECONOMICS
Reduces experimentation
Time management
Low production cost
LIGOCELLULOISE
 BIO MASS
Physiological properties
Optimization
Characterization
BIO PRODUCTS
Process quality
Yield estimation
Increased productivity
Product quality
Generation of biofuels,electricity,biodiesel,bioplastics, biochemical
Economic analysis and environmental impact
Usage and application of bio products in various aspects
Application of machine learning approaches in the lignocellulosic biorefineries
LIFECYCLE ASSESSMENT OF BIOMASS LIGNOCELLULOSIC BIOREFINERY

## Slide 24
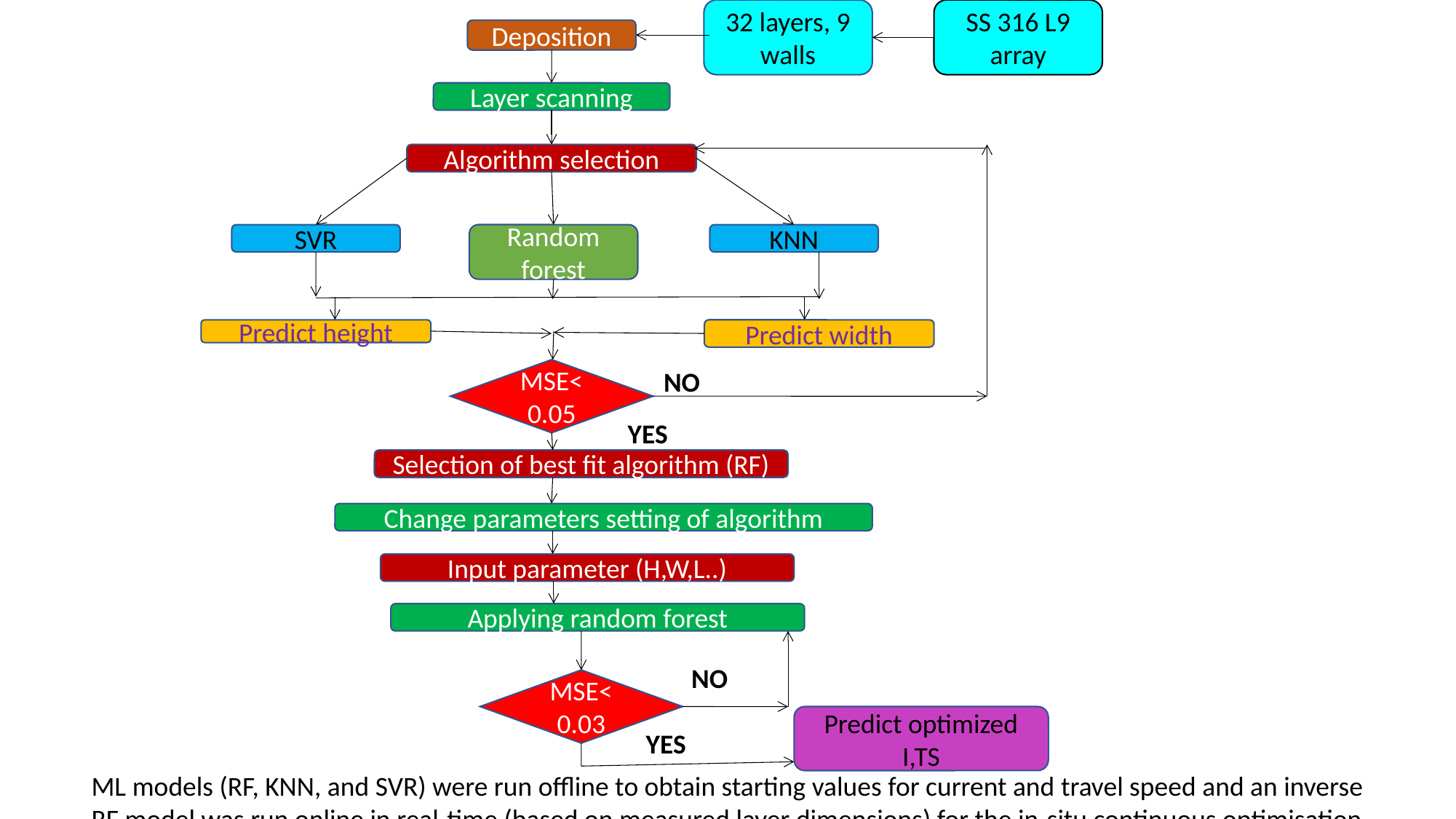

32 layers, 9 walls
SS 316 L9 array
Deposition
Layer scanning
Algorithm selection
SVR
Random forest
KNN
Predict height
Predict width
MSE< 0.05
NO
YES
Selection of best fit algorithm (RF)
Change parameters setting of algorithm
Input parameter (H,W,L..)
Applying random forest
NO
MSE< 0.03
Predict optimized I,TS
YES
ML models (RF, KNN, and SVR) were run offline to obtain starting values for current and travel speed and an inverse RF model was run online in real-time (based on measured layer dimensions) for the in-situ continuous optimisation of current and travel speed

## Slide 25
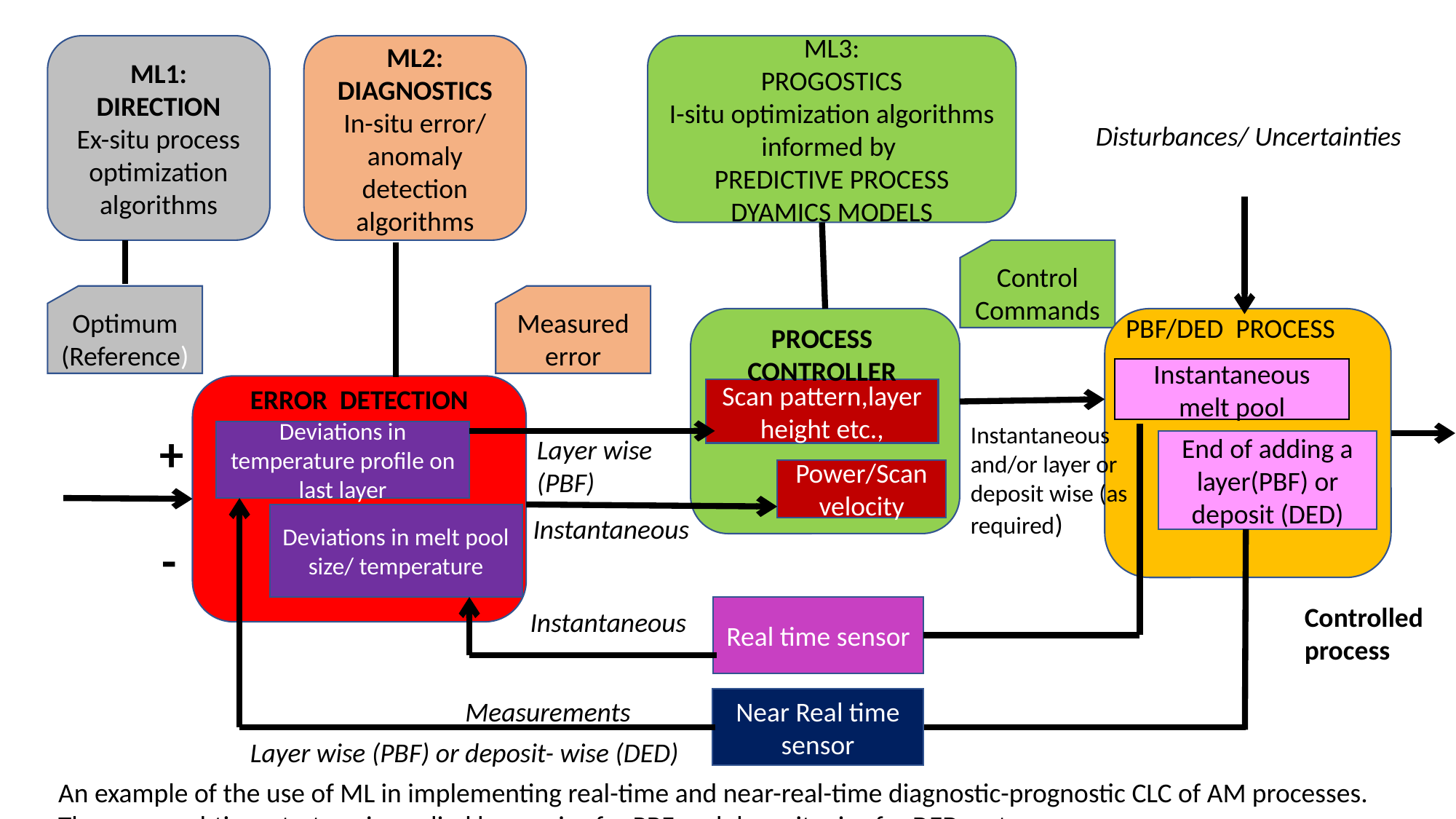

ML1:
DIRECTION
Ex-situ process optimization algorithms
ML2:
DIAGNOSTICS
In-situ error/ anomaly detection algorithms
ML3:
PROGOSTICS
I-situ optimization algorithms informed by
PREDICTIVE PROCESS
DYAMICS MODELS
Disturbances/ Uncertainties
Control
Commands
Optimum (Reference)
Measured error
PBF/DED PROCESS
PROCESS CONTROLLER
Instantaneous melt pool
ERROR DETECTION
Scan pattern,layer height etc.,
Instantaneous and/or layer or deposit wise (as required)
+
Deviations in temperature profile on last layer
Layer wise (PBF)
End of adding a layer(PBF) or deposit (DED)
Power/Scan velocity
Deviations in melt pool size/ temperature
Instantaneous
-
Controlled process
Real time sensor
Instantaneous
Measurements
Near Real time sensor
Layer wise (PBF) or deposit- wise (DED)
An example of the use of ML in implementing real-time and near-real-time diagnostic-prognostic CLC of AM processes. The near-real-time strategy is applied layer-wise for PBF and deposit-wise for DED systems

## Slide 26
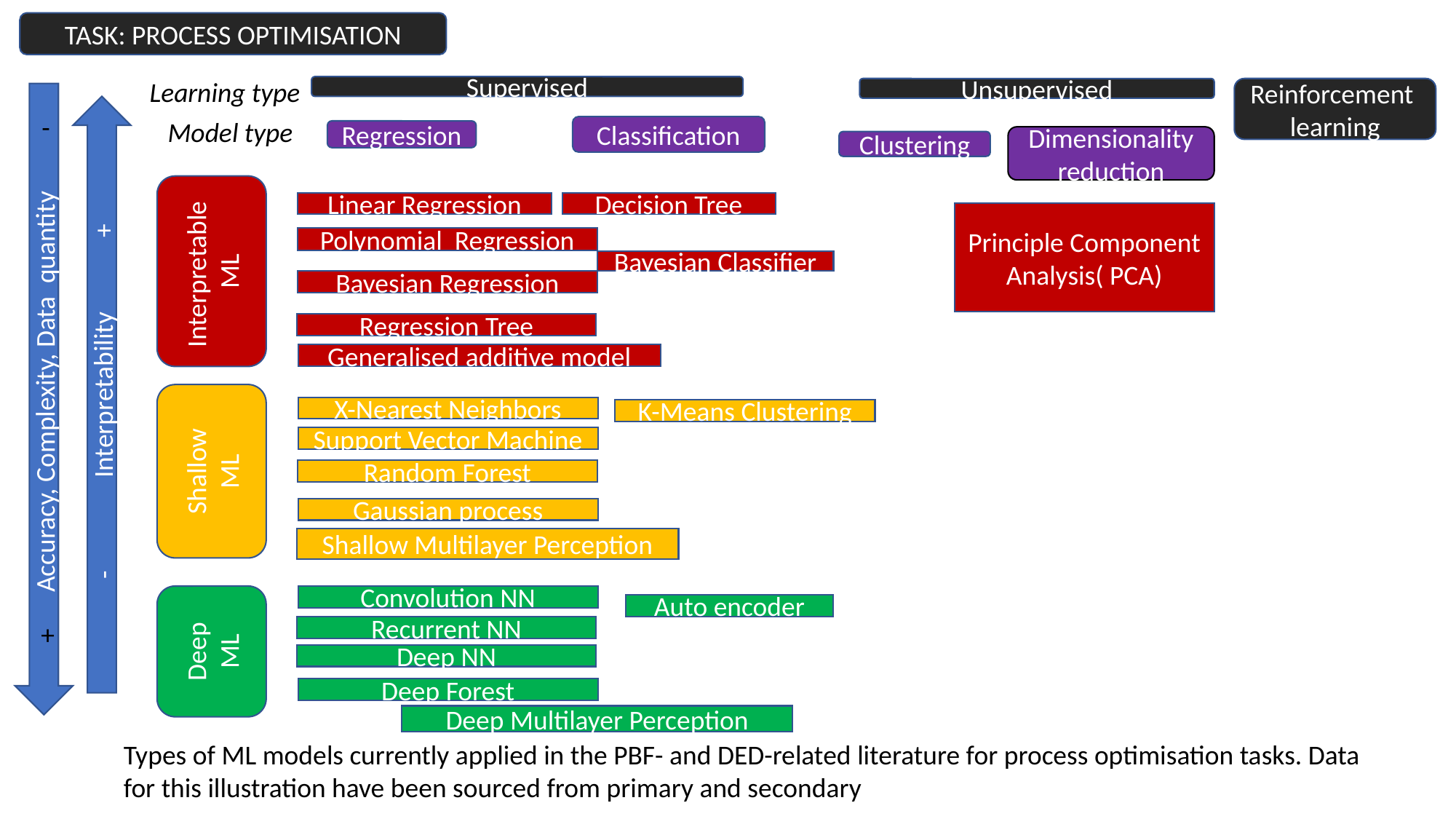

TASK: PROCESS OPTIMISATION
Learning type
Supervised
Unsupervised
Reinforcement learning
-
Model type
Classification
Regression
Dimensionality reduction
Clustering
Linear Regression
Decision Tree
Principle Component Analysis( PCA)
Interpretable ML
Polynomial Regression
Bayesian Classifier
Bayesian Regression
Regression Tree
Generalised additive model
- Interpretability +
Accuracy, Complexity, Data quantity
X-Nearest Neighbors
K-Means Clustering
Shallow
ML
Support Vector Machine
Random Forest
Gaussian process
Shallow Multilayer Perception
Convolution NN
Auto encoder
Deep ML
+
Recurrent NN
Deep NN
Deep Forest
Deep Multilayer Perception
Types of ML models currently applied in the PBF- and DED-related literature for process optimisation tasks. Data for this illustration have been sourced from primary and secondary

## Slide 27
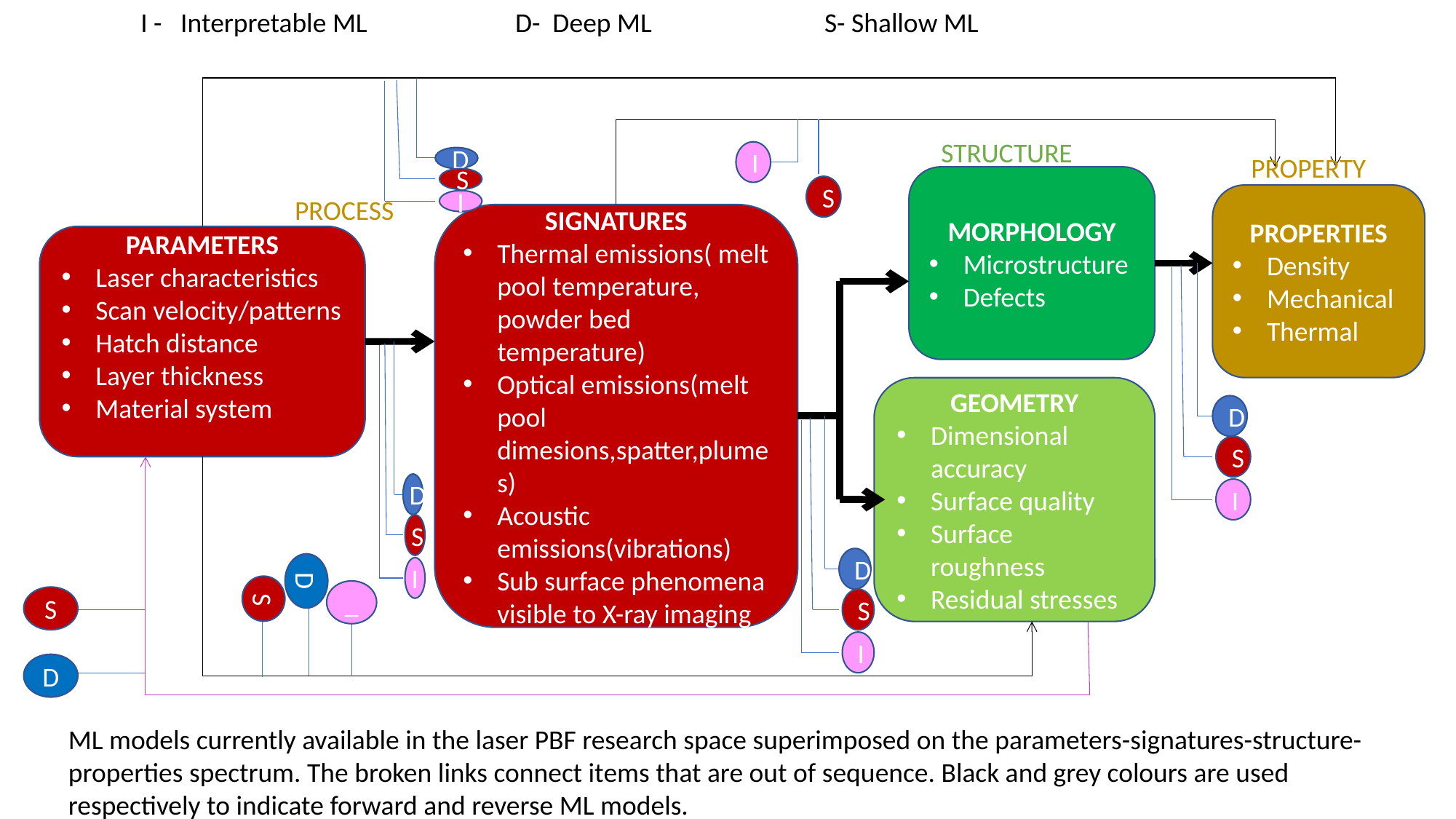

I - Interpretable ML D- Deep ML S- Shallow ML
D
S
I
STRUCTURE
I
PROPERTY
MORPHOLOGY
Microstructure
Defects
S
PROPERTIES
Density
Mechanical
Thermal
PROCESS
SIGNATURES
Thermal emissions( melt pool temperature, powder bed temperature)
Optical emissions(melt pool dimesions,spatter,plumes)
Acoustic emissions(vibrations)
Sub surface phenomena visible to X-ray imaging
PARAMETERS
Laser characteristics
Scan velocity/patterns
Hatch distance
Layer thickness
Material system
D
S
I
D
S
I
GEOMETRY
Dimensional accuracy
Surface quality
Surface roughness
Residual stresses
D
S
I
_
S
D
S
D
ML models currently available in the laser PBF research space superimposed on the parameters-signatures-structure-properties spectrum. The broken links connect items that are out of sequence. Black and grey colours are used respectively to indicate forward and reverse ML models.

## Slide 28
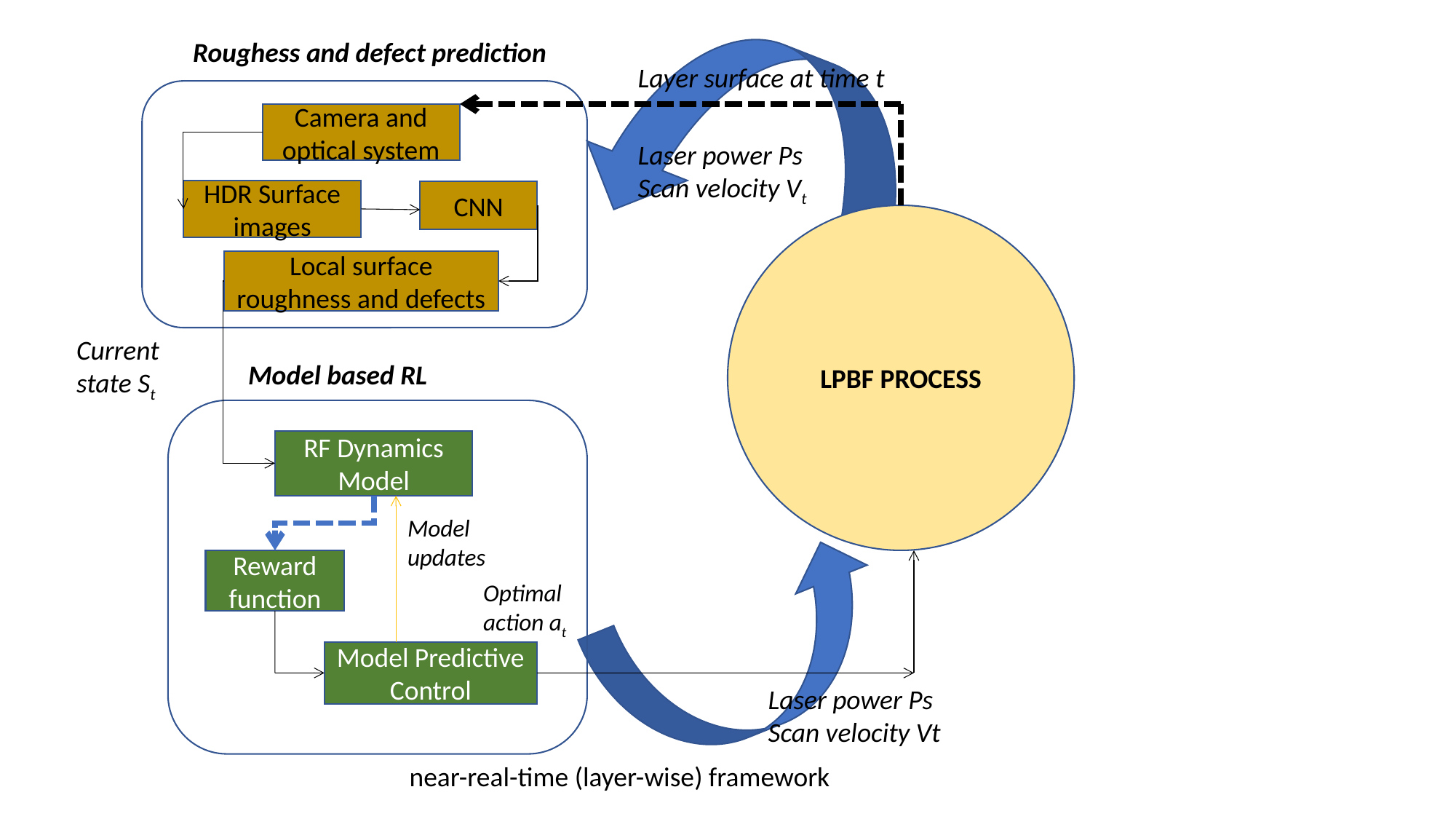

Roughess and defect prediction
Layer surface at time t
Camera and optical system
Laser power Ps
Scan velocity Vt
HDR Surface images
CNN
LPBF PROCESS
Local surface roughness and defects
Current state St
Model based RL
RF Dynamics Model
Model updates
Reward function
Optimal action at
Model Predictive Control
Laser power Ps
Scan velocity Vt
near-real-time (layer-wise) framework

## Slide 29
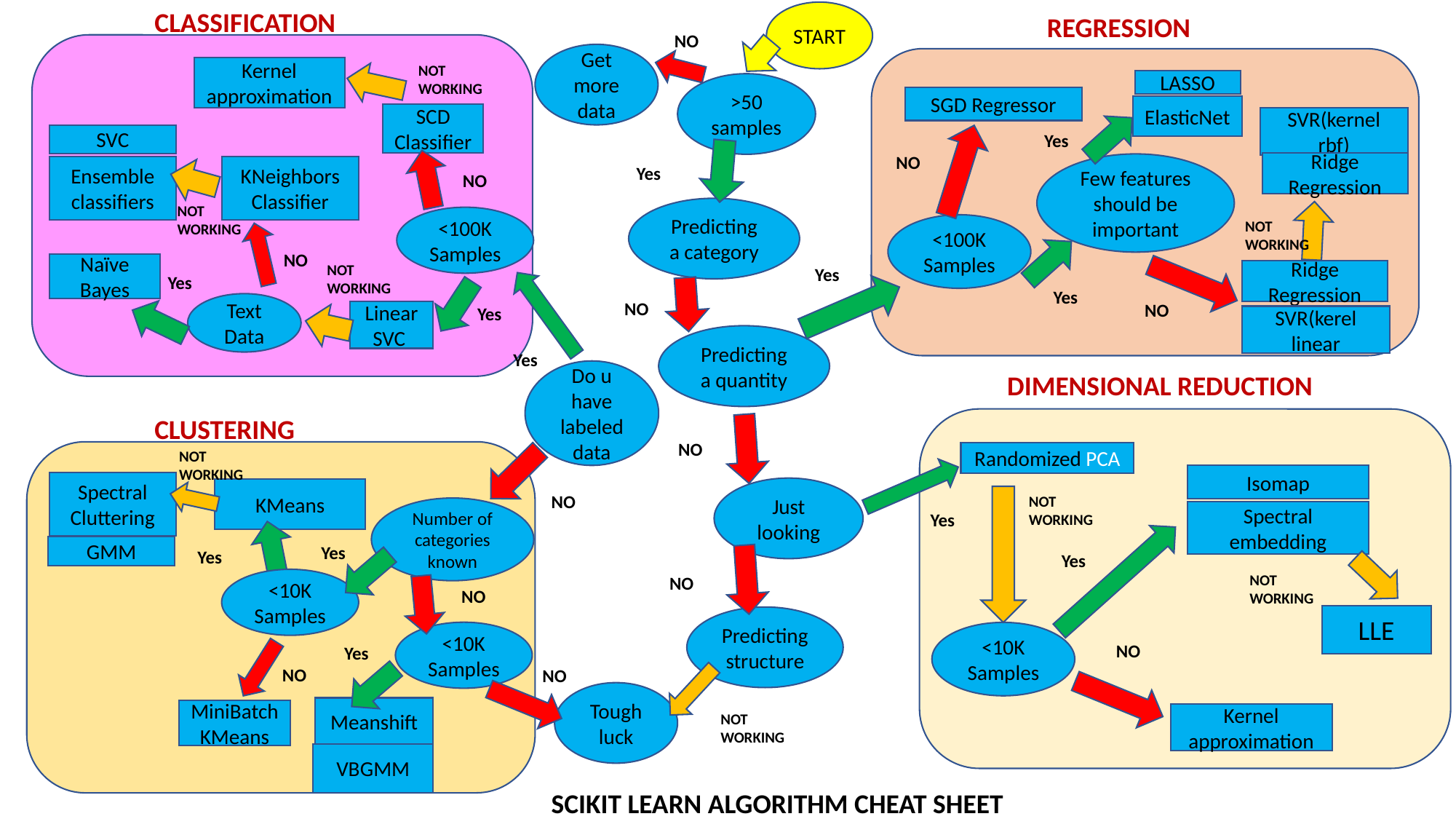

CLASSIFICATION
START
REGRESSION
NO
Get more data
NOT WORKING
Kernel approximation
LASSO
>50 samples
SGD Regressor
ElasticNet
SCD Classifier
SVR(kernel rbf)
Yes
SVC
NO
Ridge Regression
Few features should be important
Ensemble classifiers
KNeighbors Classifier
Yes
NO
NOT WORKING
Predicting a category
<100K
Samples
NOT WORKING
<100K Samples
NO
Naïve Bayes
NOT WORKING
Yes
Ridge Regression
Yes
Yes
NO
Text Data
NO
Yes
Linear SVC
SVR(kerel linear
Predicting a quantity
Yes
Do u have labeled data
DIMENSIONAL REDUCTION
CLUSTERING
NO
NOT WORKING
Randomized PCA
Isomap
Spectral Cluttering
Just looking
KMeans
NO
NOT WORKING
Number of categories known
Spectral embedding
Yes
Yes
GMM
Yes
Yes
NOT WORKING
NO
<10K
Samples
NO
LLE
Predicting structure
<10K
Samples
<10K Samples
NO
Yes
NO
NO
Tough luck
Meanshift
MiniBatch KMeans
NOT WORKING
Kernel approximation
VBGMM
SCIKIT LEARN ALGORITHM CHEAT SHEET

## Slide 30
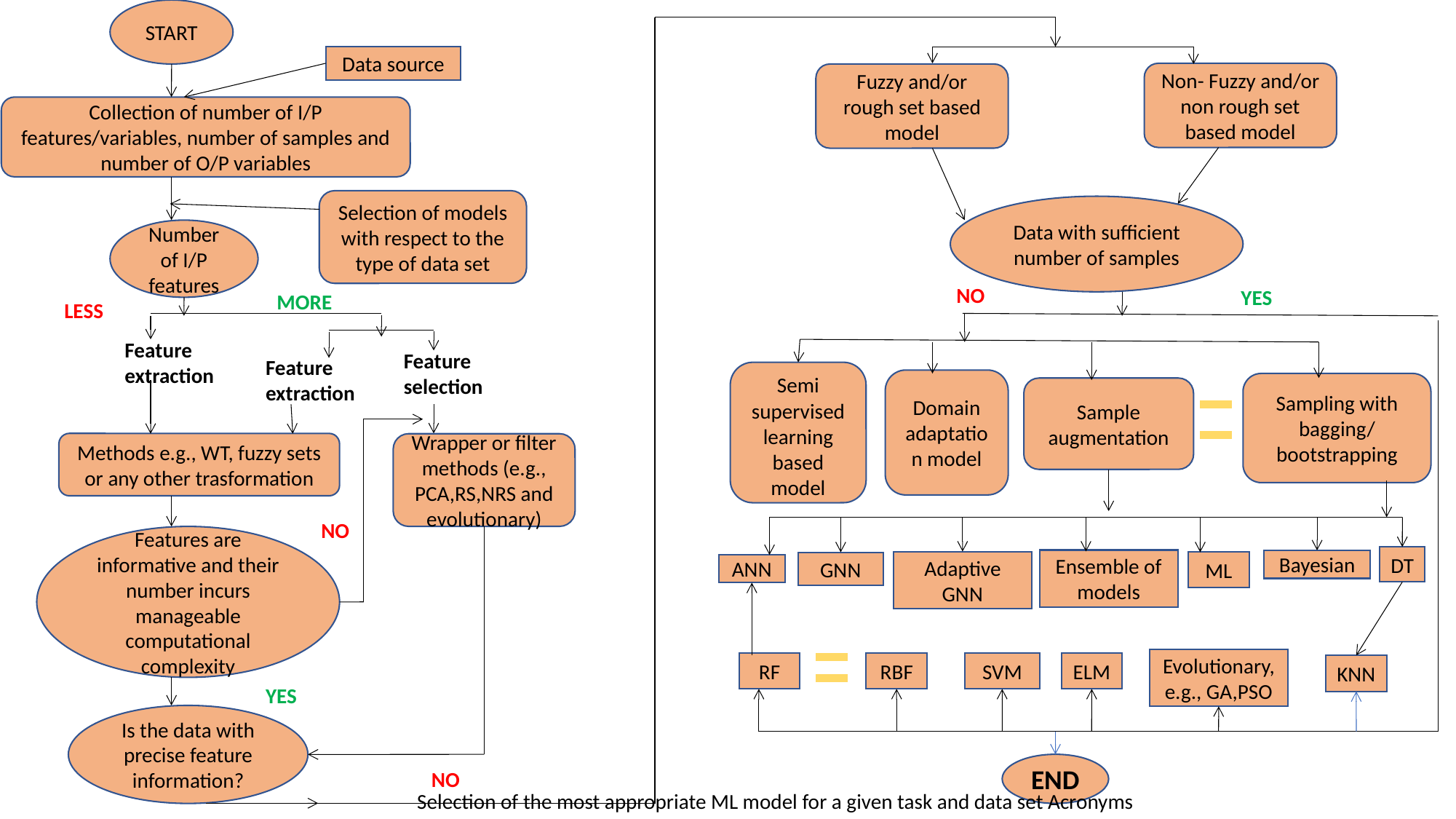

START
Data source
Non- Fuzzy and/or non rough set based model
Fuzzy and/or rough set based model
Collection of number of I/P features/variables, number of samples and number of O/P variables
Selection of models with respect to the type of data set
Data with sufficient number of samples
Number of I/P features
NO
YES
MORE
LESS
Feature extraction
Feature selection
Feature extraction
Semi supervised learning based model
Domain adaptation model
Sampling with bagging/ bootstrapping
Sample augmentation
Methods e.g., WT, fuzzy sets or any other trasformation
Wrapper or filter methods (e.g., PCA,RS,NRS and evolutionary)
NO
Features are informative and their number incurs manageable computational complexity
DT
Ensemble of models
Bayesian
Adaptive GNN
ML
GNN
ANN
Evolutionary, e.g., GA,PSO
RF
RBF
SVM
ELM
KNN
YES
Is the data with precise feature information?
END
NO
Selection of the most appropriate ML model for a given task and data set Acronyms used: I/P – Input; O/P – Output; WT – Wavelet Transform; PCA – Principal Component Analysis

## Slide 31
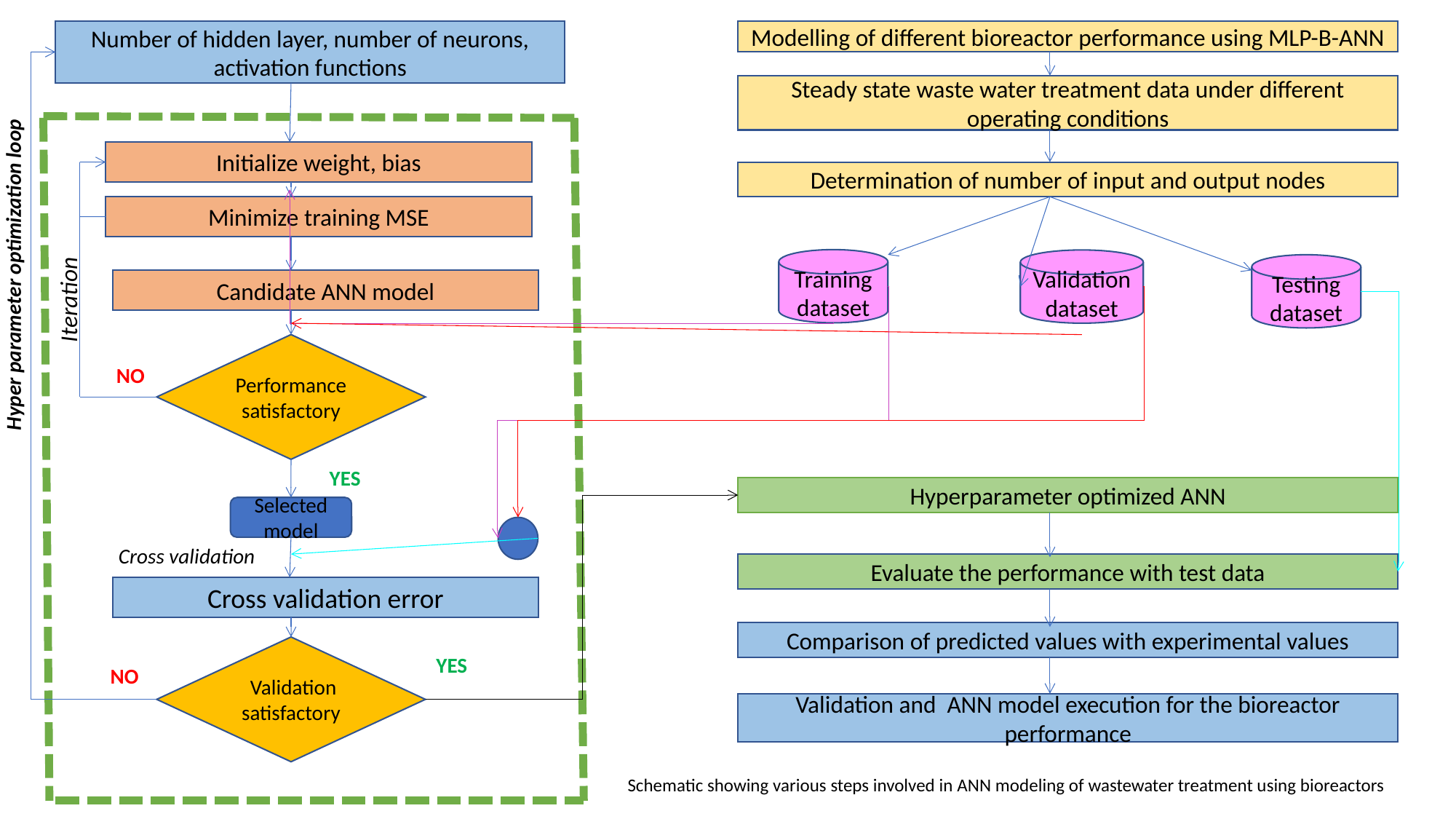

Number of hidden layer, number of neurons, activation functions
Modelling of different bioreactor performance using MLP-B-ANN
Steady state waste water treatment data under different operating conditions
Initialize weight, bias
Determination of number of input and output nodes
Minimize training MSE
Training dataset
Validation dataset
Iteration
Testing dataset
Hyper parameter optimization loop
Candidate ANN model
Performance satisfactory
NO
YES
Hyperparameter optimized ANN
Selected model
Cross validation
Evaluate the performance with test data
Cross validation error
Comparison of predicted values with experimental values
 Validation satisfactory
YES
NO
Validation and ANN model execution for the bioreactor performance
Schematic showing various steps involved in ANN modeling of wastewater treatment using bioreactors

## Slide 32
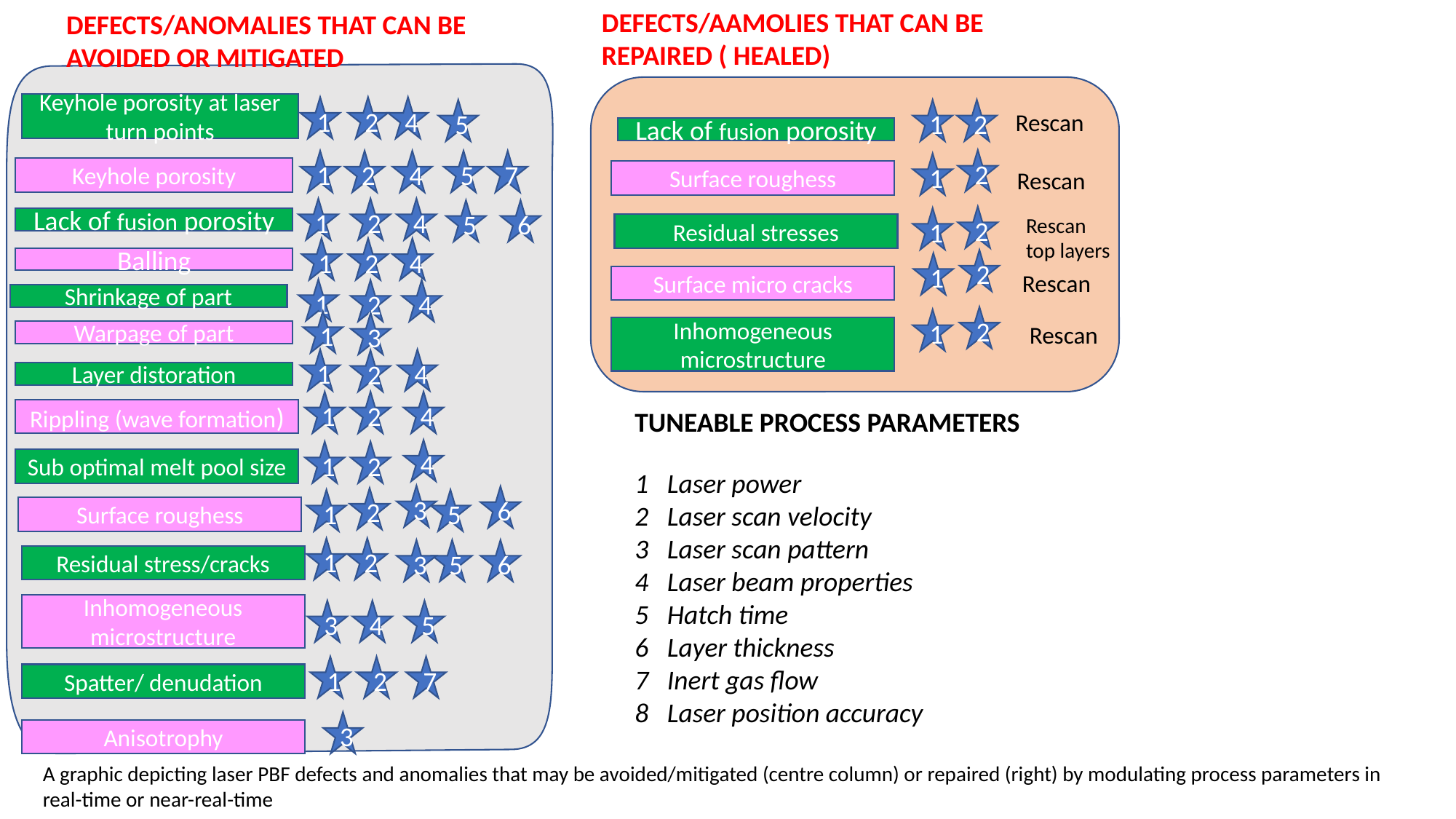

DEFECTS/AAMOLIES THAT CAN BE REPAIRED ( HEALED)
DEFECTS/ANOMALIES THAT CAN BE AVOIDED OR MITIGATED
Keyhole porosity at laser turn points
1
2
4
5
1
2
Rescan
Lack of fusion porosity
2
1
2
4
5
7
1
Keyhole porosity
Rescan
Surface roughess
1
2
4
5
6
2
Rescan top layers
Lack of fusion porosity
1
Residual stresses
1
2
4
Balling
2
1
Rescan
Surface micro cracks
1
2
4
Shrinkage of part
2
1
1
3
Rescan
Inhomogeneous microstructure
Warpage of part
1
4
2
Layer distoration
1
2
4
Rippling (wave formation)
TUNEABLE PROCESS PARAMETERS
4
1
2
Sub optimal melt pool size
1 Laser power
2 Laser scan velocity
3 Laser scan pattern
4 Laser beam properties
5 Hatch time
6 Layer thickness
7 Inert gas flow
8 Laser position accuracy
3
6
2
1
5
Surface roughess
1
2
3
5
6
Residual stress/cracks
Inhomogeneous microstructure
3
4
5
1
2
7
Spatter/ denudation
3
Anisotrophy
A graphic depicting laser PBF defects and anomalies that may be avoided/mitigated (centre column) or repaired (right) by modulating process parameters in real-time or near-real-time

## Slide 33
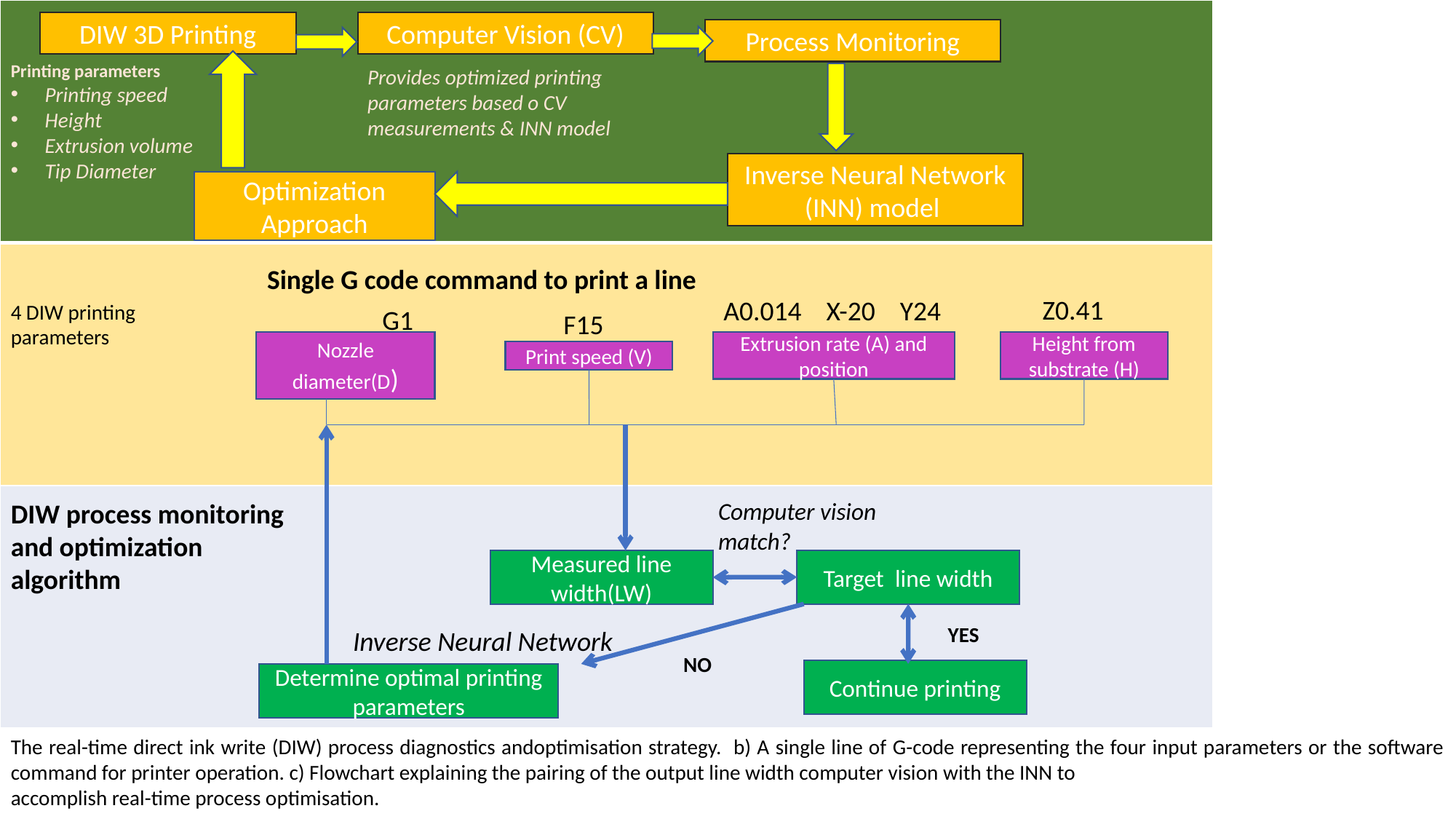

| |
| --- |
| |
| |
DIW 3D Printing
Computer Vision (CV)
Process Monitoring
Printing parameters
Printing speed
Height
Extrusion volume
Tip Diameter
Provides optimized printing parameters based o CV measurements & INN model
Inverse Neural Network (INN) model
Optimization Approach
Single G code command to print a line
Z0.41
A0.014 X-20 Y24
4 DIW printing parameters
G1
F15
Nozzle diameter(D)
Extrusion rate (A) and position
Height from substrate (H)
Print speed (V)
DIW process monitoring and optimization algorithm
Computer vision match?
Measured line width(LW)
Target line width
YES
Inverse Neural Network
NO
Continue printing
Determine optimal printing parameters
The real-time direct ink write (DIW) process diagnostics andoptimisation strategy. b) A single line of G-code representing the four input parameters or the software command for printer operation. c) Flowchart explaining the pairing of the output line width computer vision with the INN to
accomplish real-time process optimisation.

## Slide 34
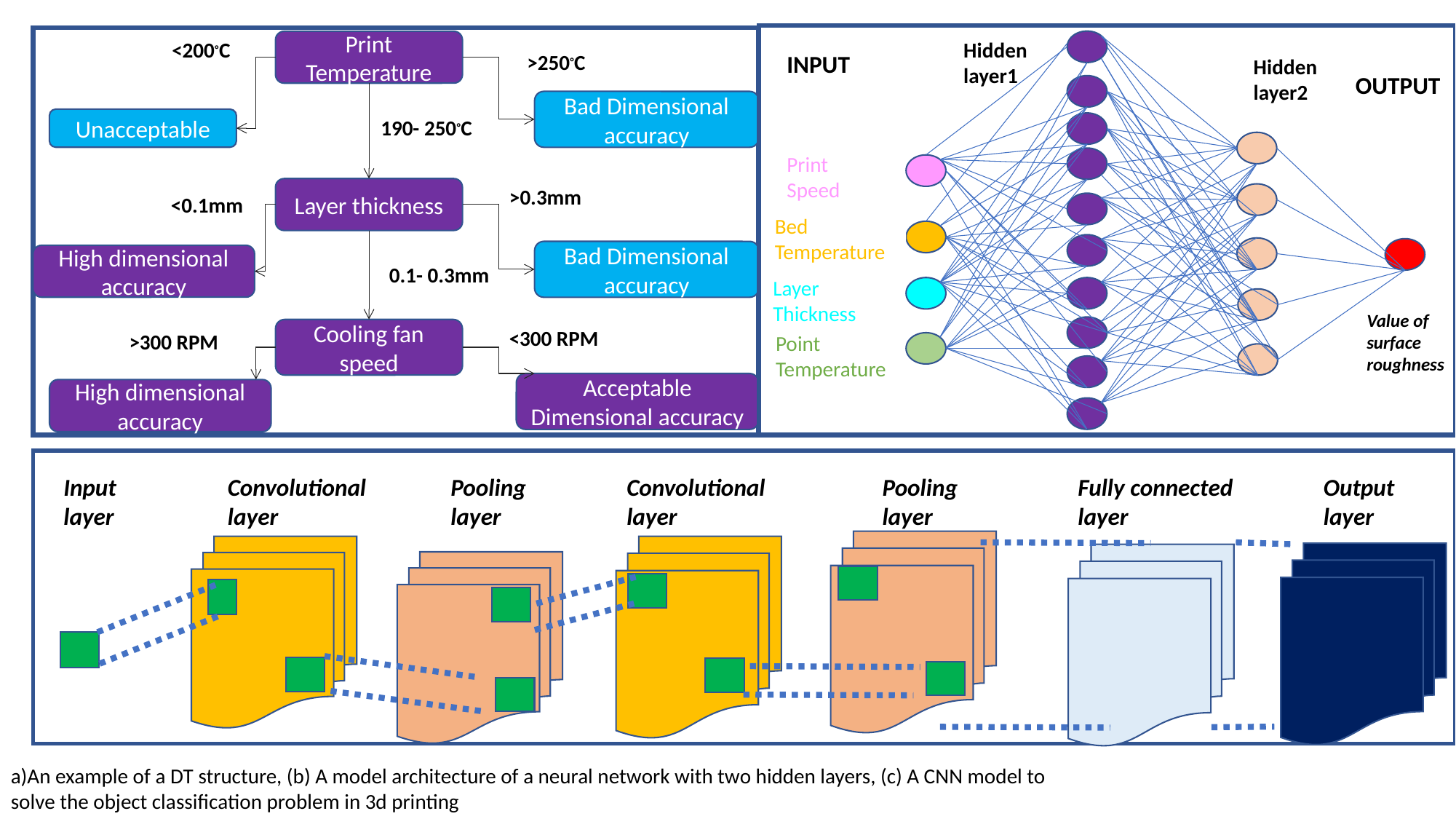

<200◦C
Print Temperature
Hidden layer1
>250◦C
INPUT
Hidden layer2
OUTPUT
Bad Dimensional accuracy
190- 250◦C
Unacceptable
Print Speed
Layer thickness
>0.3mm
<0.1mm
Bed Temperature
Bad Dimensional accuracy
High dimensional accuracy
0.1- 0.3mm
Layer Thickness
Value of surface roughness
Cooling fan speed
<300 RPM
>300 RPM
Point Temperature
Acceptable Dimensional accuracy
High dimensional accuracy
Input layer
Pooling layer
Convolutional layer
Pooling layer
Output layer
Convolutional layer
Fully connected layer
a)An example of a DT structure, (b) A model architecture of a neural network with two hidden layers, (c) A CNN model to solve the object classification problem in 3d printing

## Slide 35
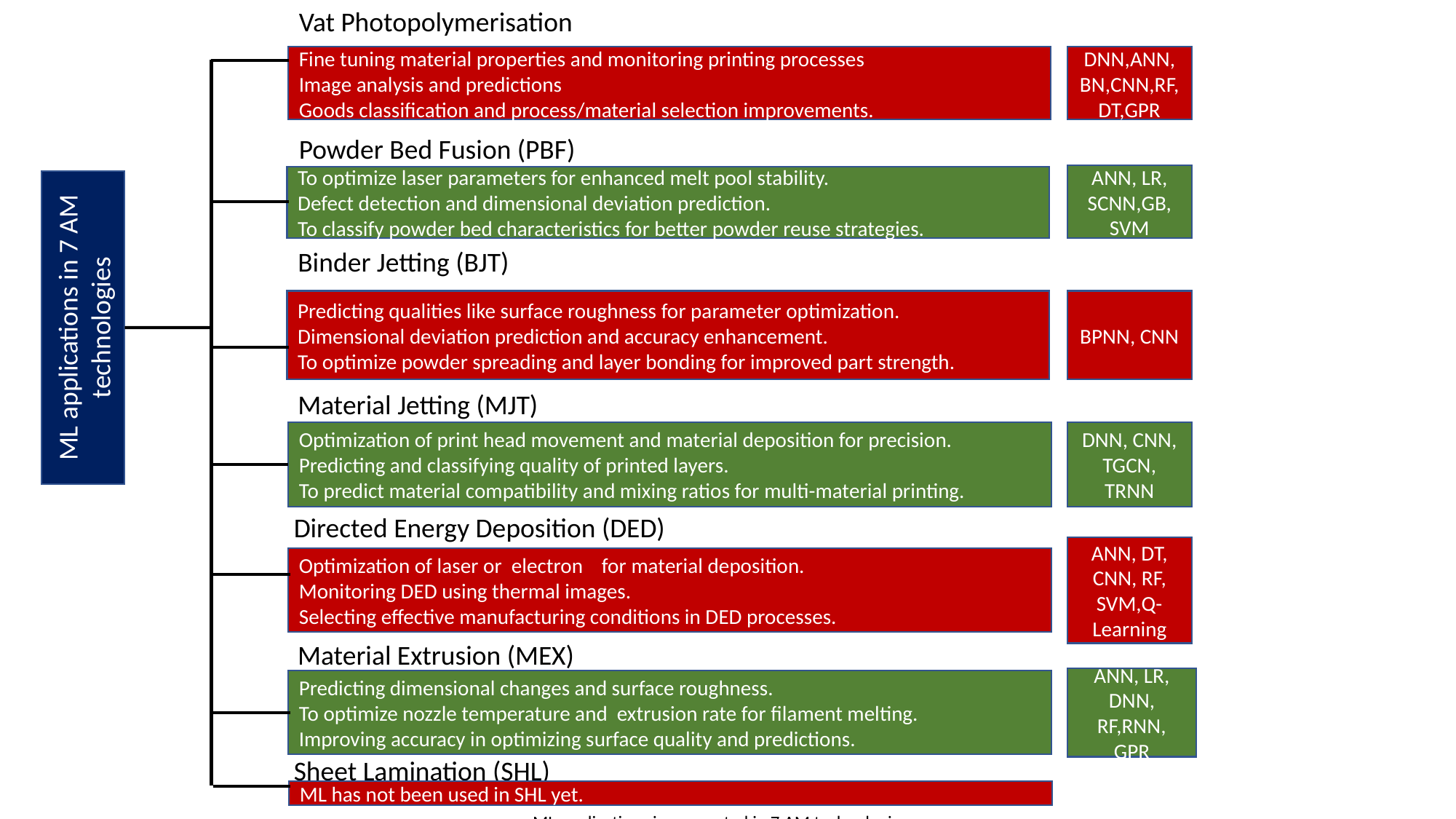

Vat Photopolymerisation
Fine tuning material properties and monitoring printing processes
Image analysis and predictions
Goods classification and process/material selection improvements.
DNN,ANN,
BN,CNN,RF,DT,GPR
Powder Bed Fusion (PBF)
ANN, LR, SCNN,GB,
SVM
To optimize laser parameters for enhanced melt pool stability.
Defect detection and dimensional deviation prediction.
To classify powder bed characteristics for better powder reuse strategies.
Binder Jetting (BJT)
ML applications in 7 AM technologies
Predicting qualities like surface roughness for parameter optimization.
Dimensional deviation prediction and accuracy enhancement.
To optimize powder spreading and layer bonding for improved part strength.
BPNN, CNN
Material Jetting (MJT)
Optimization of print head movement and material deposition for precision.
Predicting and classifying quality of printed layers.
To predict material compatibility and mixing ratios for multi-material printing.
DNN, CNN, TGCN, TRNN
Directed Energy Deposition (DED)
ANN, DT, CNN, RF, SVM,Q- Learning
Optimization of laser or electron for material deposition.
Monitoring DED using thermal images.
Selecting effective manufacturing conditions in DED processes.
Material Extrusion (MEX)
ANN, LR, DNN, RF,RNN, GPR
Predicting dimensional changes and surface roughness.
To optimize nozzle temperature and extrusion rate for filament melting.
Improving accuracy in optimizing surface quality and predictions.
Sheet Lamination (SHL)
ML has not been used in SHL yet.
ML applications incorporated in 7 AM technologies

## Slide 36
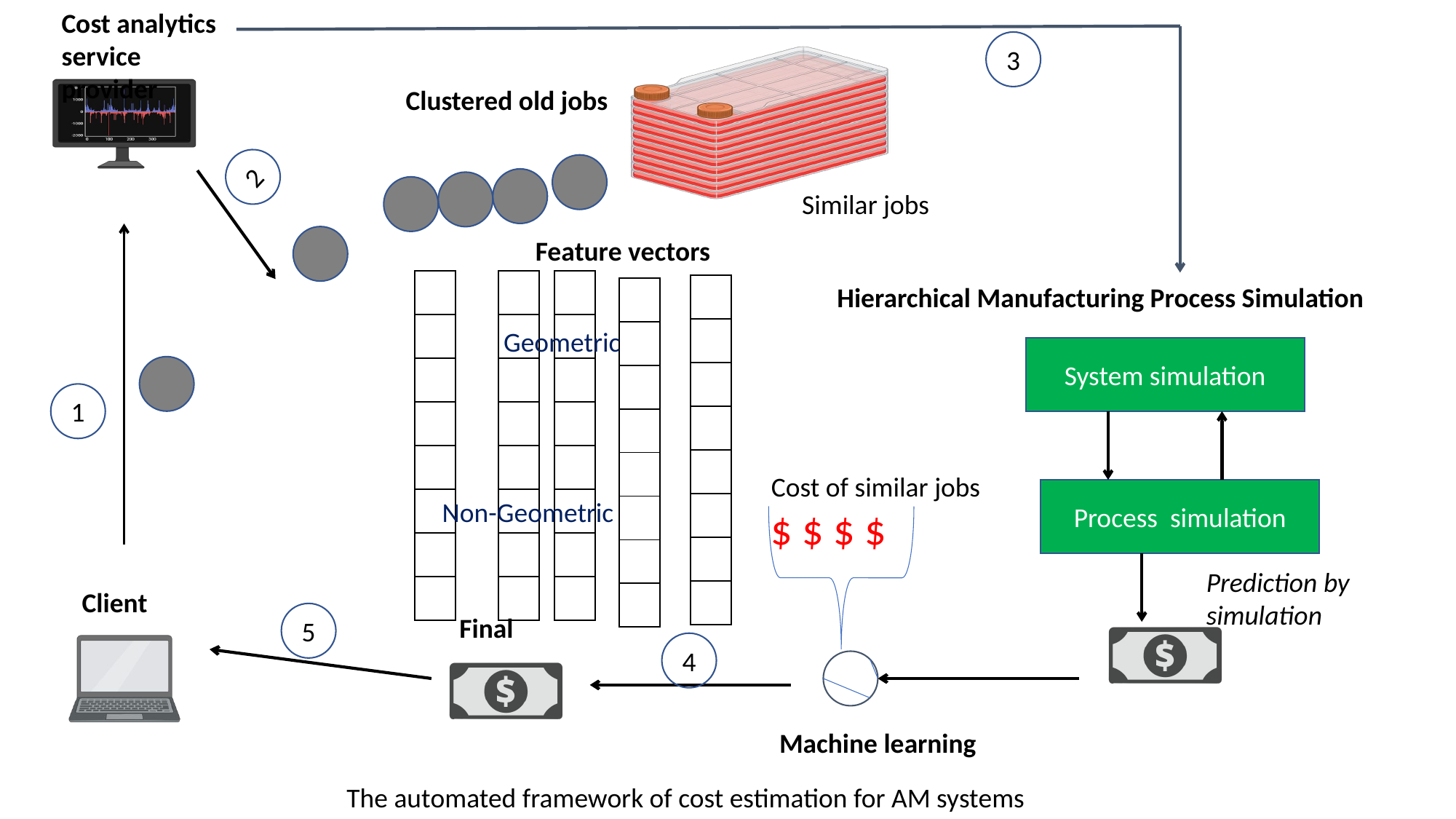

Cost analytics service provider
3
Clustered old jobs
2
Similar jobs
Feature vectors
| |
| --- |
| |
| |
| |
| |
| |
| |
| |
| |
| --- |
| |
| |
| |
| |
| |
| |
| |
| |
| --- |
| |
| |
| |
| |
| |
| |
| |
Hierarchical Manufacturing Process Simulation
| |
| --- |
| |
| |
| |
| |
| |
| |
| |
| |
| --- |
| |
| |
| |
| |
| |
| |
| |
Geometric
System simulation
1
Cost of similar jobs
$ $ $ $
Process simulation
Non-Geometric
Prediction by simulation
Client
5
Final
4
Machine learning
The automated framework of cost estimation for AM systems

## Slide 37
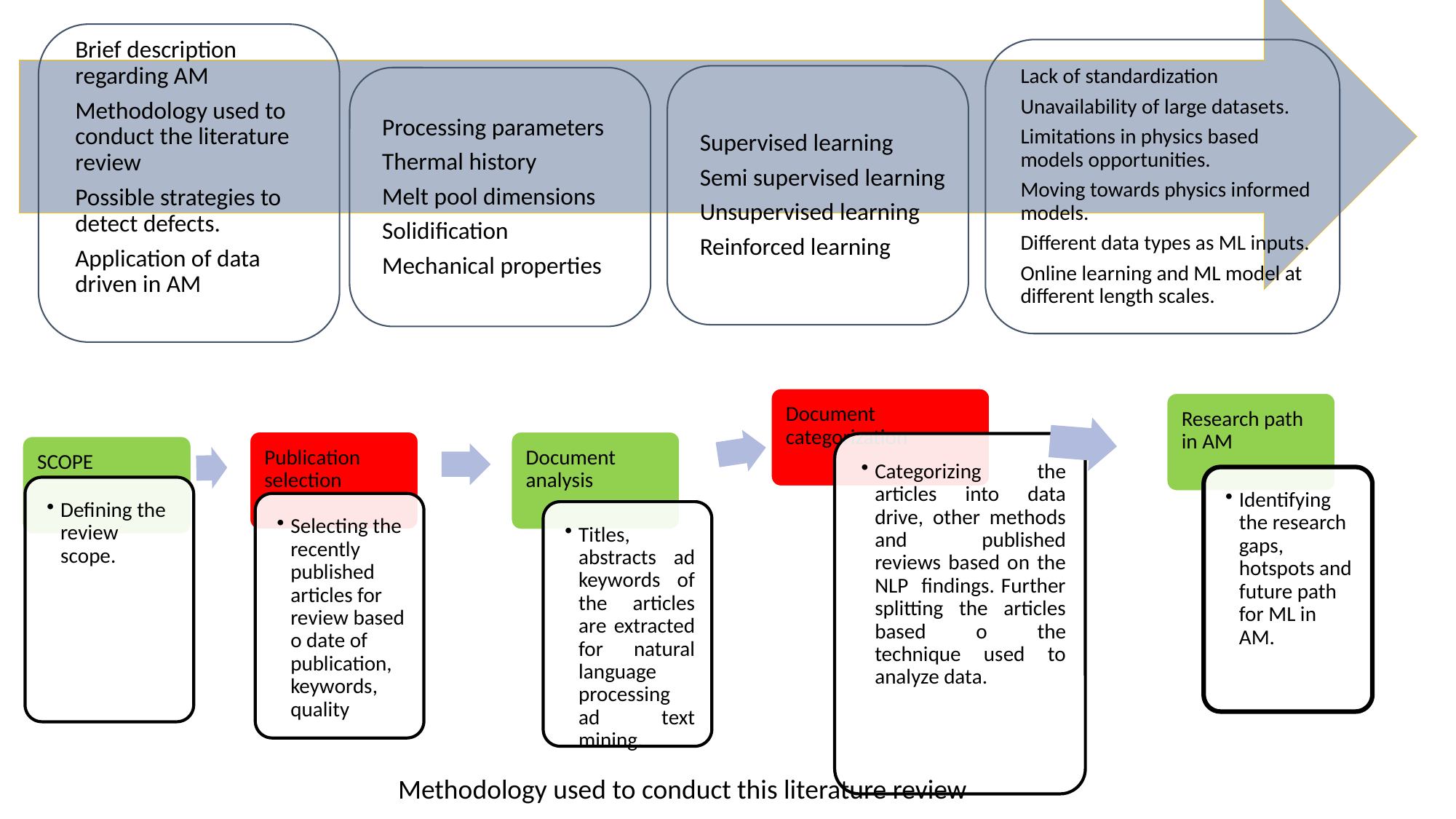

Methodology used to conduct this literature review

## Slide 38
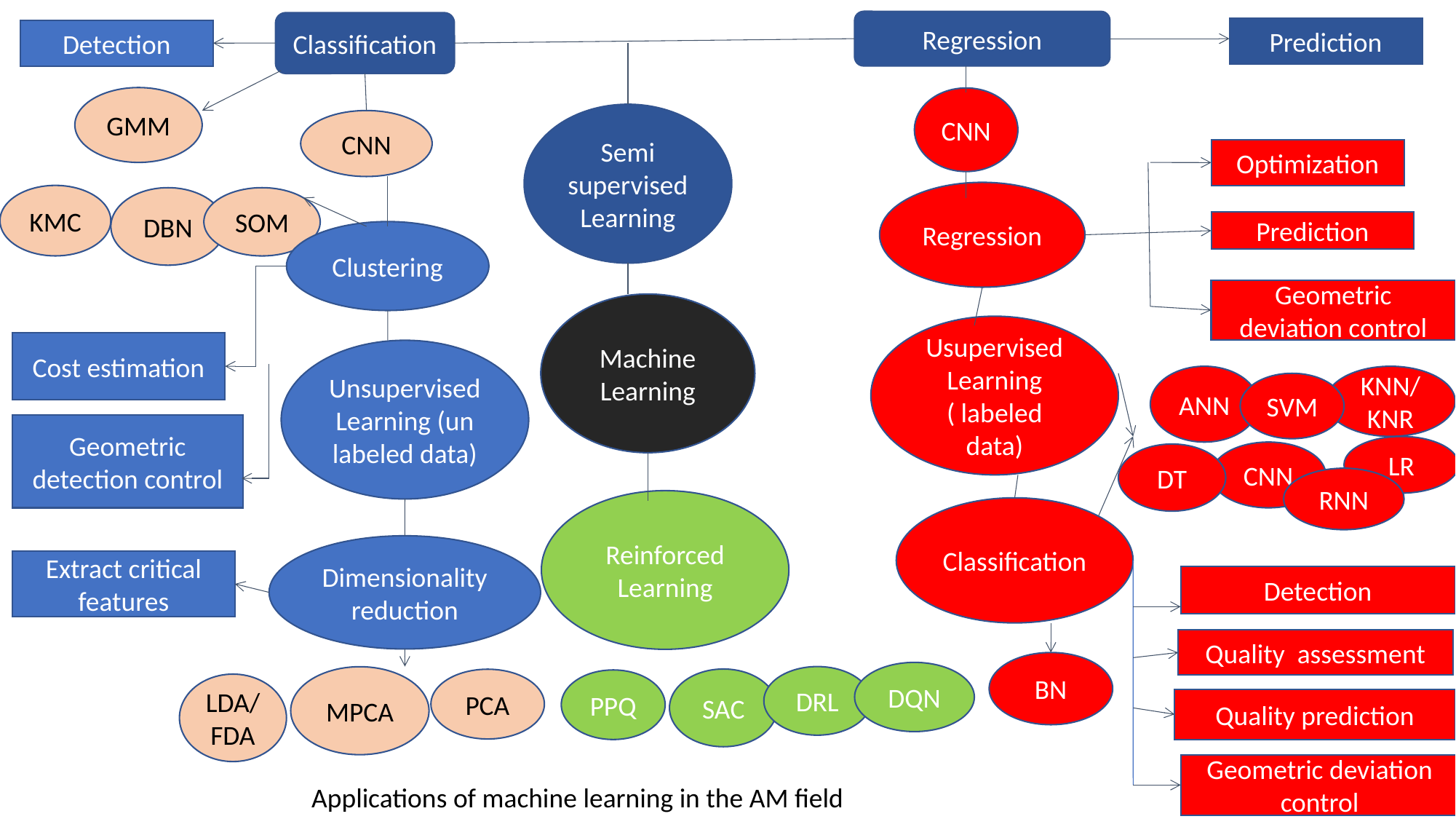

Regression
Classification
Prediction
Detection
GMM
CNN
Semi supervised Learning
CNN
Optimization
Regression
KMC
DBN
SOM
Prediction
Clustering
Geometric deviation control
Machine
Learning
Usupervised Learning ( labeled data)
Cost estimation
Unsupervised Learning (un labeled data)
ANN
KNN/ KNR
SVM
Geometric detection control
LR
CNN
DT
RNN
Reinforced Learning
Classification
Dimensionality reduction
Extract critical features
Detection
Quality assessment
BN
DQN
DRL
MPCA
SAC
PCA
PPQ
LDA/ FDA
Quality prediction
Geometric deviation control
Applications of machine learning in the AM field

## Slide 39
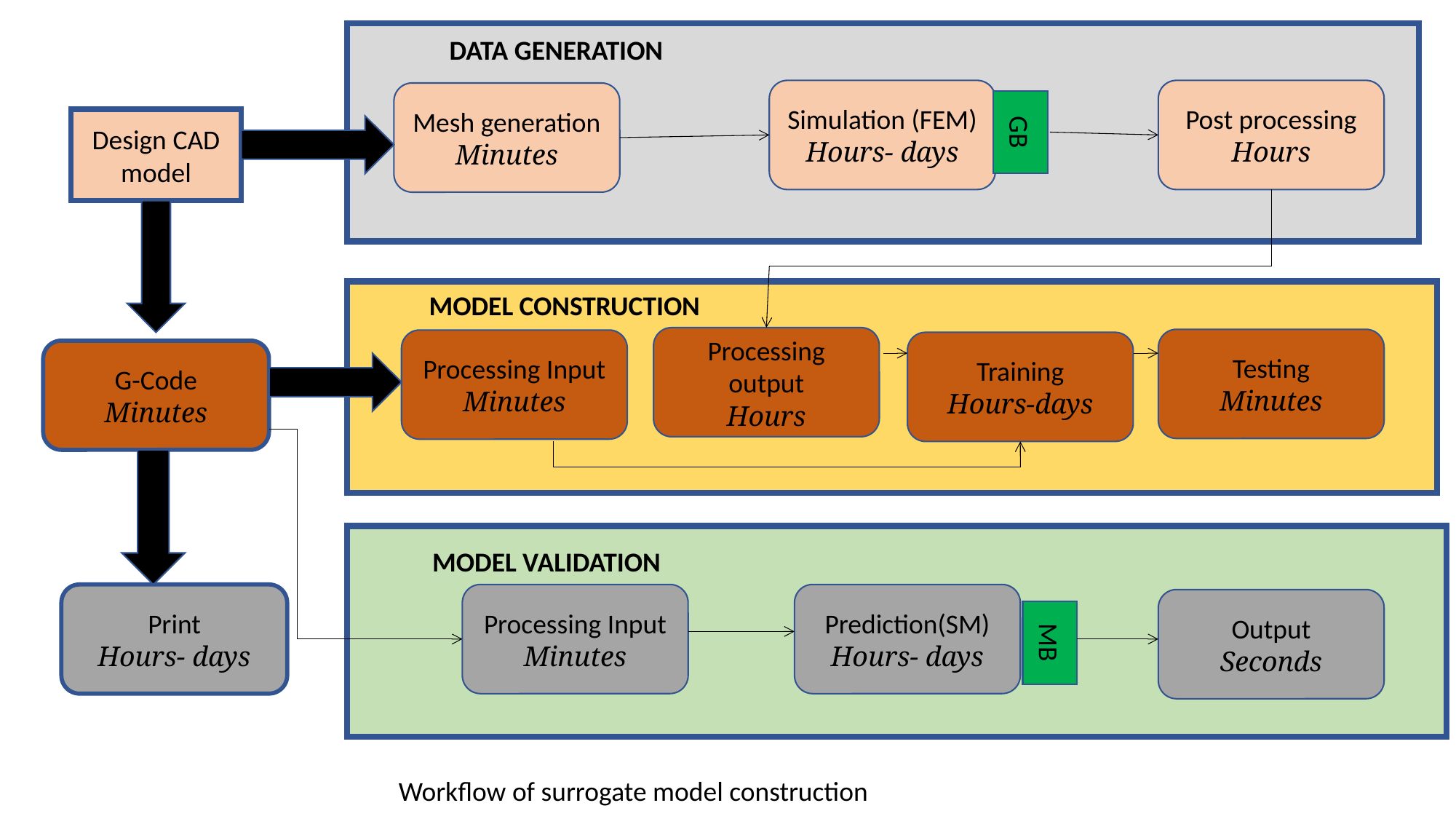

DATA GENERATION
Simulation (FEM)
Hours- days
Post processing
Hours
Mesh generation Minutes
GB
Design CAD model
MODEL CONSTRUCTION
Processing output
Hours
Testing
Minutes
Processing Input
Minutes
Training
Hours-days
G-Code
Minutes
MODEL VALIDATION
Print
Hours- days
Processing Input
Minutes
Prediction(SM)
Hours- days
Output
Seconds
MB
Workflow of surrogate model construction

## Slide 40
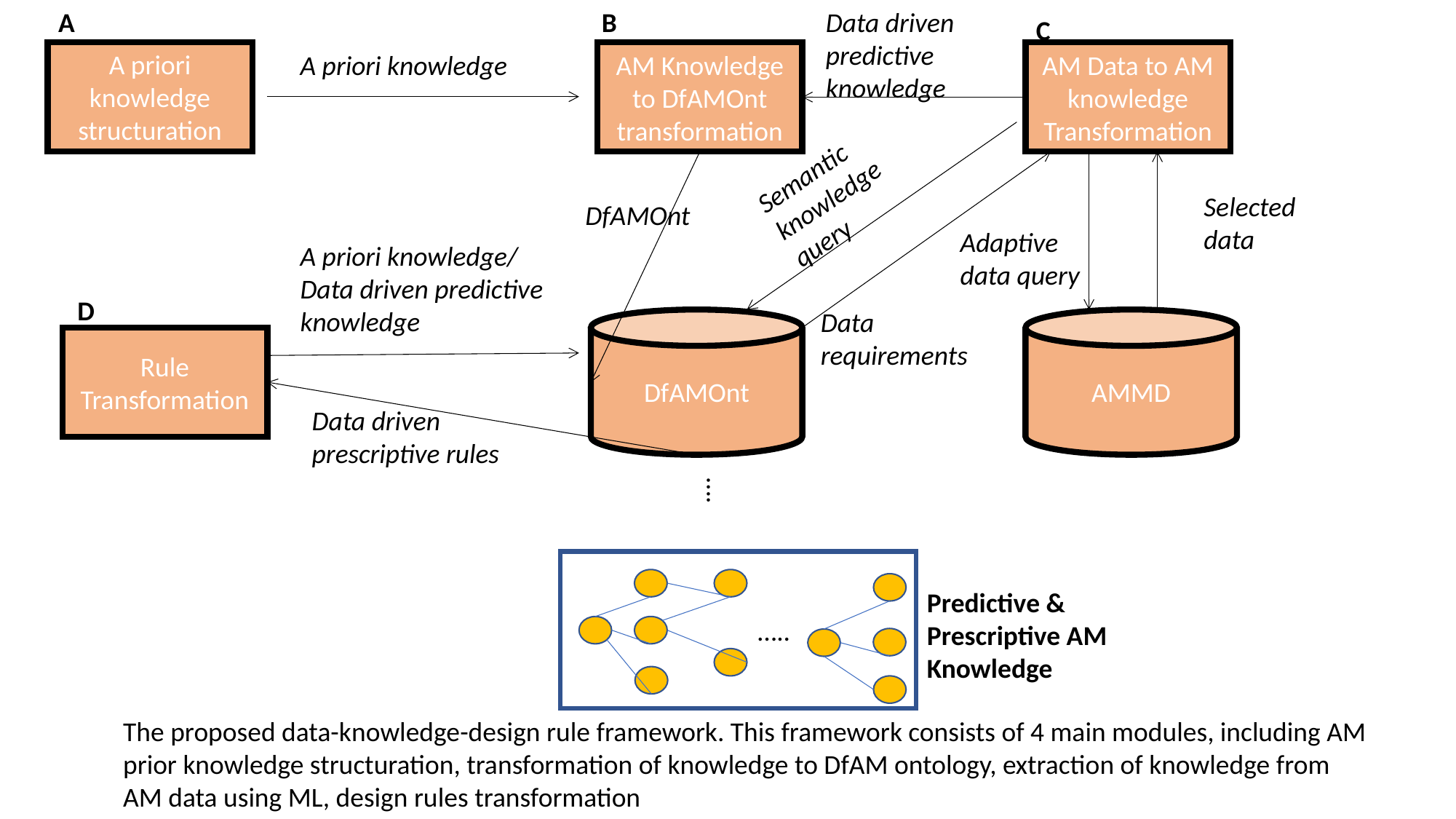

A
B
Data driven predictive knowledge
C
A priori knowledge structuration
A priori knowledge
AM Knowledge to DfAMOnt transformation
AM Data to AM knowledge Transformation
Semantic knowledge query
Selected data
DfAMOnt
Adaptive
data query
A priori knowledge/ Data driven predictive knowledge
D
Data requirements
DfAMOnt
AMMD
Rule Transformation
Data driven prescriptive rules
….
Predictive & Prescriptive AM Knowledge
…..
The proposed data-knowledge-design rule framework. This framework consists of 4 main modules, including AM prior knowledge structuration, transformation of knowledge to DfAM ontology, extraction of knowledge from AM data using ML, design rules transformation

## Slide 41
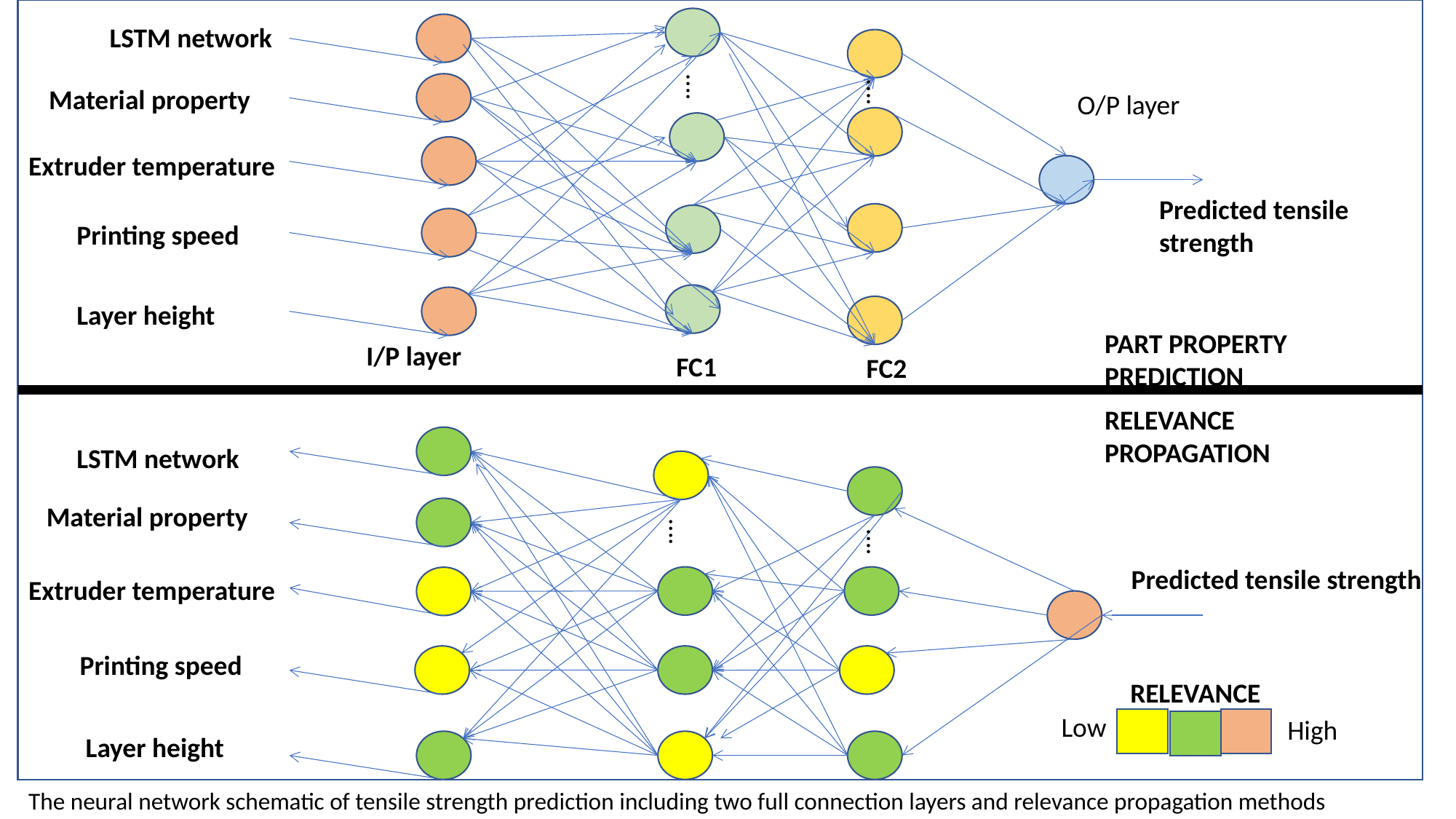

LSTM network
….
….
Material property
O/P layer
Extruder temperature
Predicted tensile strength
Printing speed
Layer height
PART PROPERTY PREDICTION
I/P layer
FC1
FC2
RELEVANCE PROPAGATION
LSTM network
Material property
….
….
Predicted tensile strength
Extruder temperature
Printing speed
RELEVANCE
Low
High
Layer height
The neural network schematic of tensile strength prediction including two full connection layers and relevance propagation methods

## Slide 42
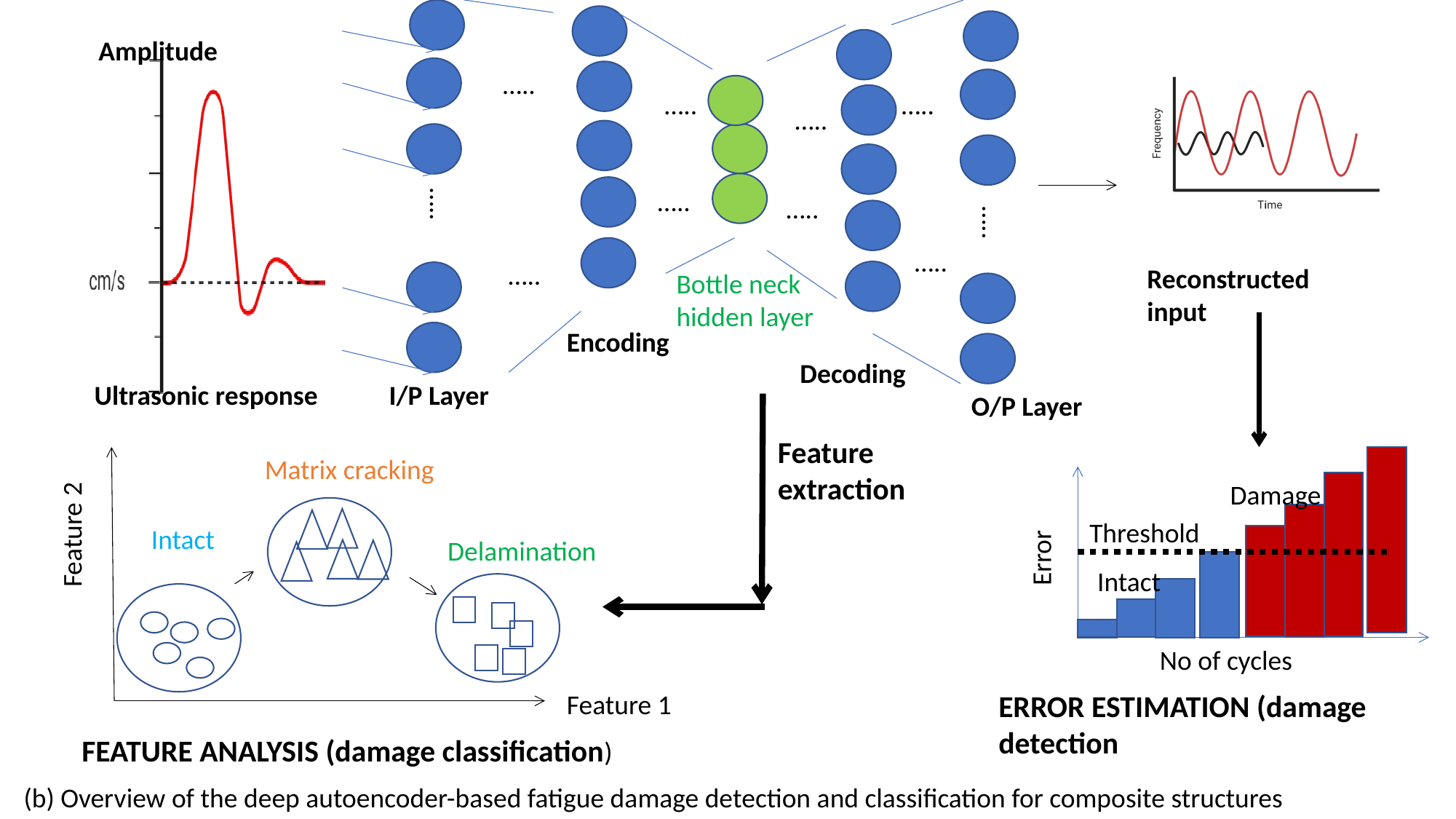

Amplitude
…..
…..
…..
…..
…..
…..
…..
…..
…..
…..
Reconstructed input
Bottle neck hidden layer
Encoding
Decoding
Ultrasonic response
I/P Layer
O/P Layer
Feature extraction
Matrix cracking
Damage
Feature 2
Threshold
Intact
Delamination
Error
Intact
No of cycles
Feature 1
ERROR ESTIMATION (damage detection
FEATURE ANALYSIS (damage classification)
(b) Overview of the deep autoencoder-based fatigue damage detection and classification for composite structures

## Slide 43
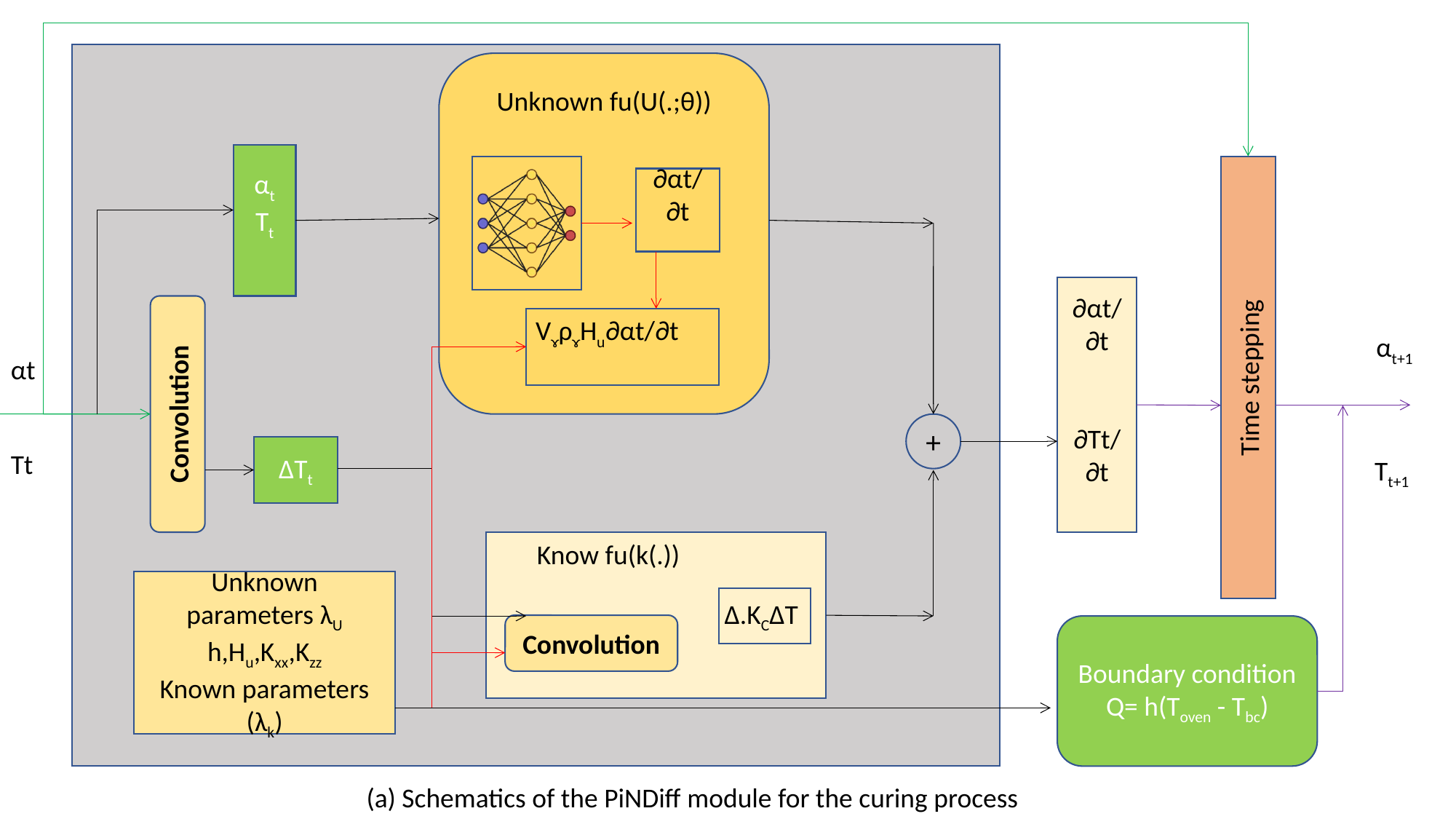

Unknown fu(U(.;θ))
 VɤρɤHu∂αt/∂t
αt Tt
∂αt/∂t
∂αt/∂t
∂Tt/∂t
αt+1
αt
Time stepping
Convolution
+
ΔTt
Tt
Tt+1
 Δ.KCΔT
Know fu(k(.))
Unknown parameters λU h,Hu,Kxx,Kzz
Known parameters (λk)
Convolution
Boundary condition
Q= h(Toven - Tbc)
(a) Schematics of the PiNDiff module for the curing process

## Slide 44
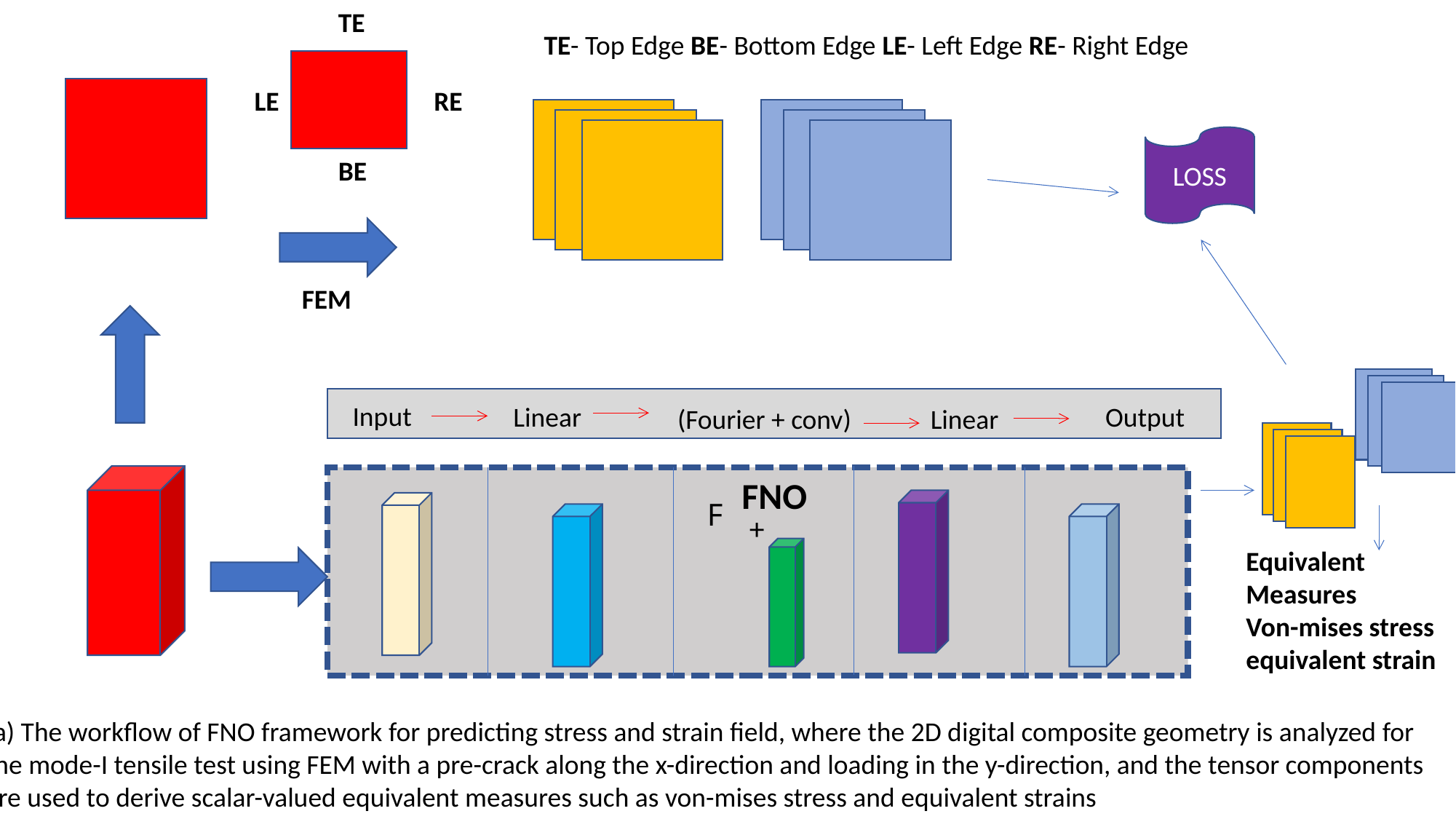

TE
TE- Top Edge BE- Bottom Edge LE- Left Edge RE- Right Edge
LE
RE
LOSS
BE
FEM
Input
Linear
Output
 (Fourier + conv)
 Linear
FNO
F
+
Equivalent Measures
Von-mises stress equivalent strain
(a) The workflow of FNO framework for predicting stress and strain field, where the 2D digital composite geometry is analyzed for the mode-I tensile test using FEM with a pre-crack along the x-direction and loading in the y-direction, and the tensor components are used to derive scalar-valued equivalent measures such as von-mises stress and equivalent strains

## Slide 45
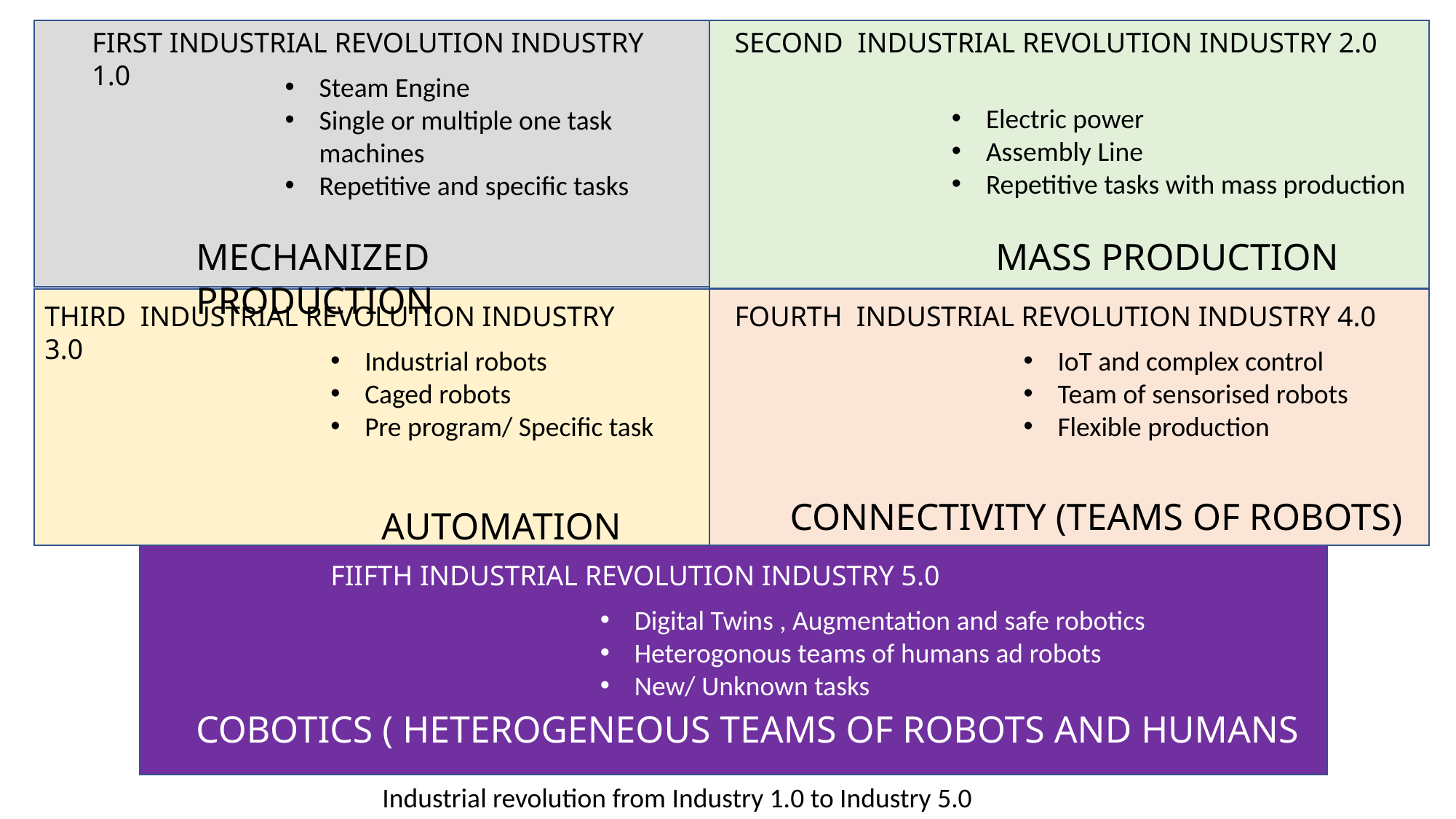

FIRST INDUSTRIAL REVOLUTION INDUSTRY 1.0
SECOND INDUSTRIAL REVOLUTION INDUSTRY 2.0
Steam Engine
Single or multiple one task machines
Repetitive and specific tasks
Electric power
Assembly Line
Repetitive tasks with mass production
MECHANIZED PRODUCTION
MASS PRODUCTION
THIRD INDUSTRIAL REVOLUTION INDUSTRY 3.0
FOURTH INDUSTRIAL REVOLUTION INDUSTRY 4.0
Industrial robots
Caged robots
Pre program/ Specific task
IoT and complex control
Team of sensorised robots
Flexible production
CONNECTIVITY (TEAMS OF ROBOTS)
AUTOMATION
FIIFTH INDUSTRIAL REVOLUTION INDUSTRY 5.0
Digital Twins , Augmentation and safe robotics
Heterogonous teams of humans ad robots
New/ Unknown tasks
COBOTICS ( HETEROGENEOUS TEAMS OF ROBOTS AND HUMANS
Industrial revolution from Industry 1.0 to Industry 5.0

## Slide 46
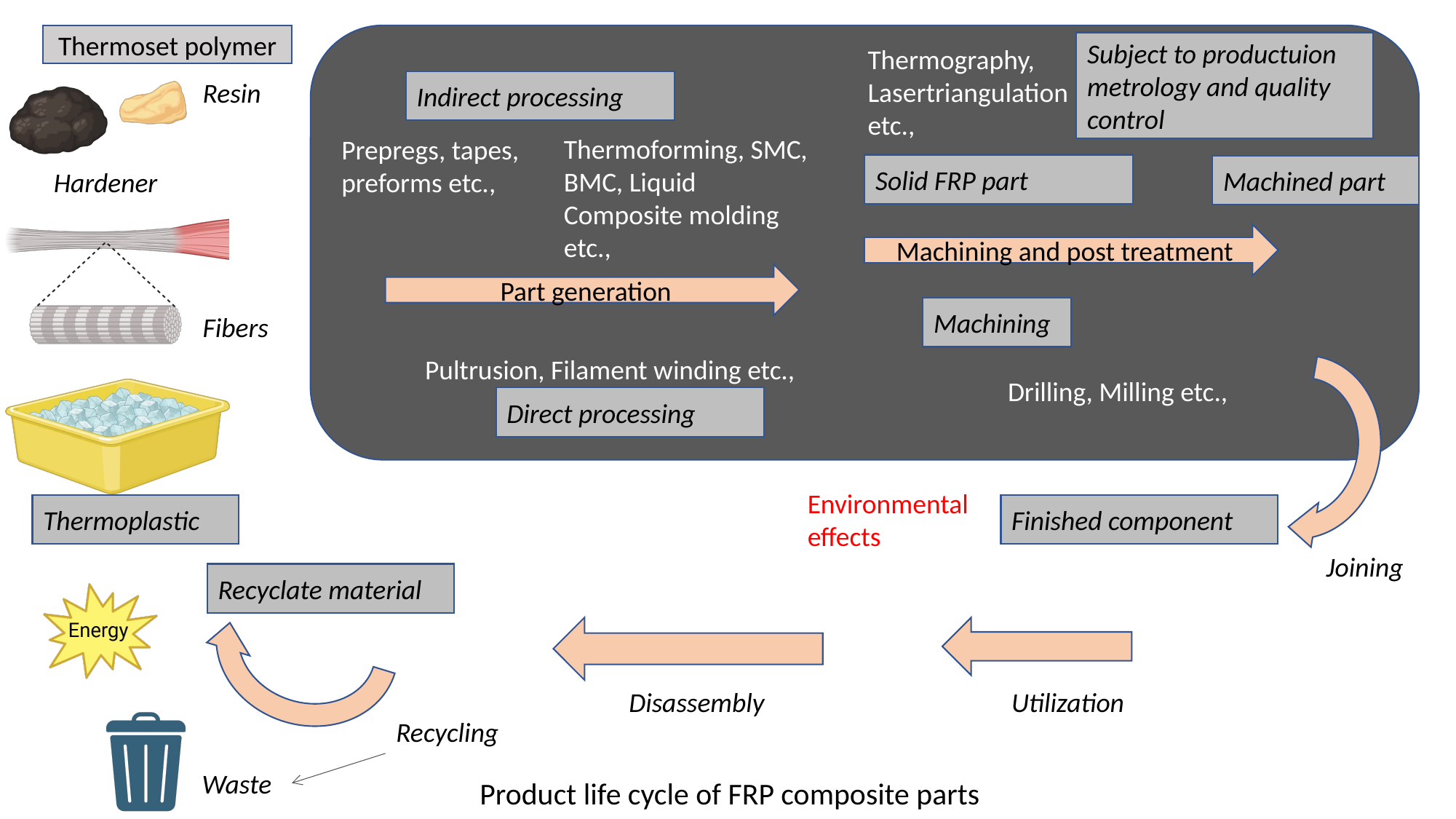

Thermoset polymer
Subject to productuion metrology and quality control
Thermography, Lasertriangulation etc.,
Resin
Indirect processing
Thermoforming, SMC, BMC, Liquid Composite molding etc.,
Prepregs, tapes, preforms etc.,
Solid FRP part
Machined part
Hardener
Machining and post treatment
Part generation
Machining
Fibers
Pultrusion, Filament winding etc.,
Drilling, Milling etc.,
Direct processing
Environmental effects
Thermoplastic
Finished component
Joining
Recyclate material
Disassembly
Utilization
Recycling
Waste
Product life cycle of FRP composite parts

## Slide 47
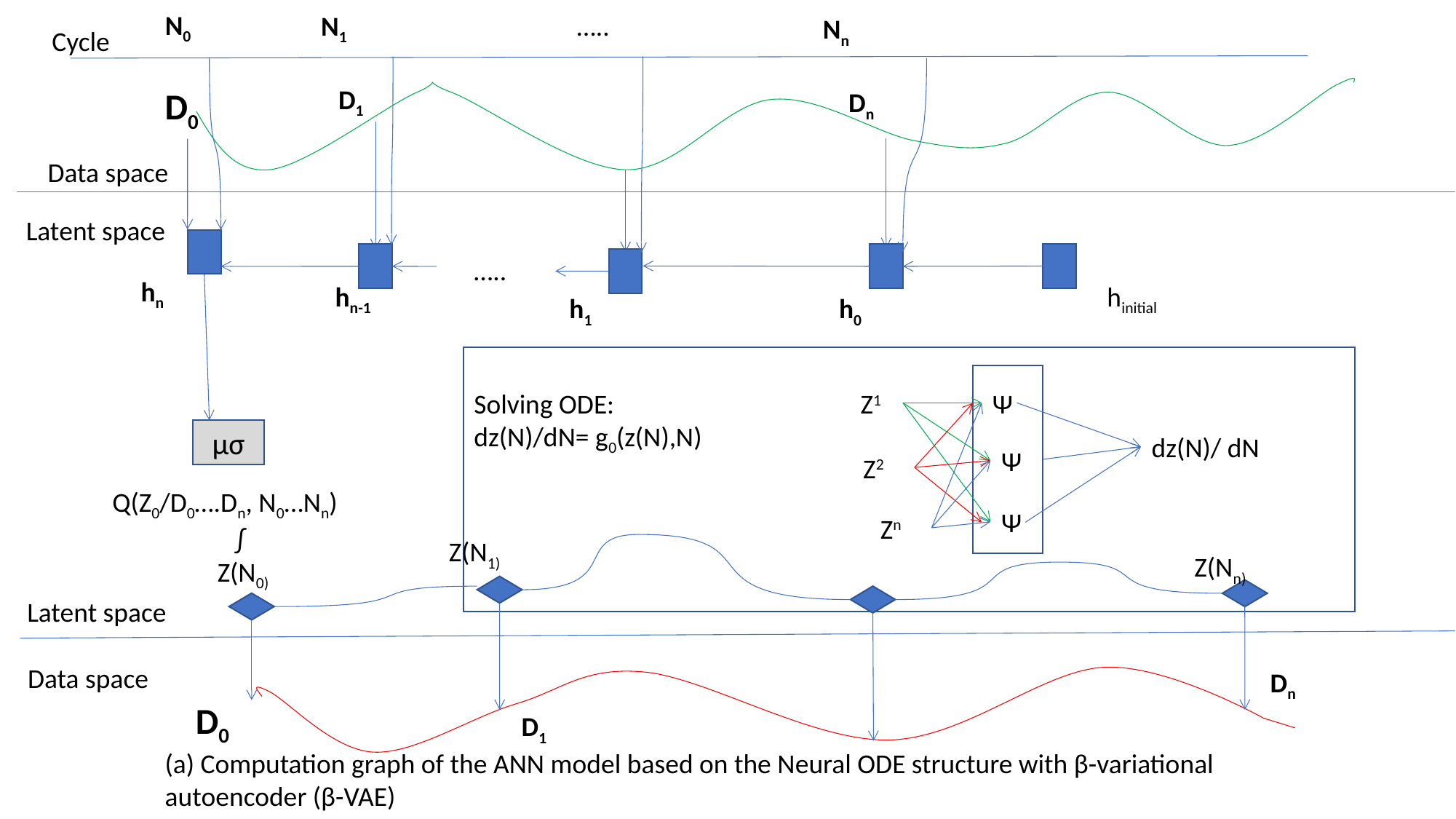

N0
N1
…..
Nn
Cycle
D1
D0
Dn
Data space
Latent space
…..
hn
hn-1
hinitial
h1
h0
Solving ODE:
dz(N)/dN= g0(z(N),N)
Z1
Ψ
μσ
dz(N)/ dN
Ψ
Z2
Q(Z0/D0….Dn, N0…Nn)
 ∫
 Z(N0)
Ψ
Zn
Z(N1)
Z(Nn)
Latent space
Data space
Dn
D0
D1
(a) Computation graph of the ANN model based on the Neural ODE structure with β-variational autoencoder (β-VAE)

## Slide 48
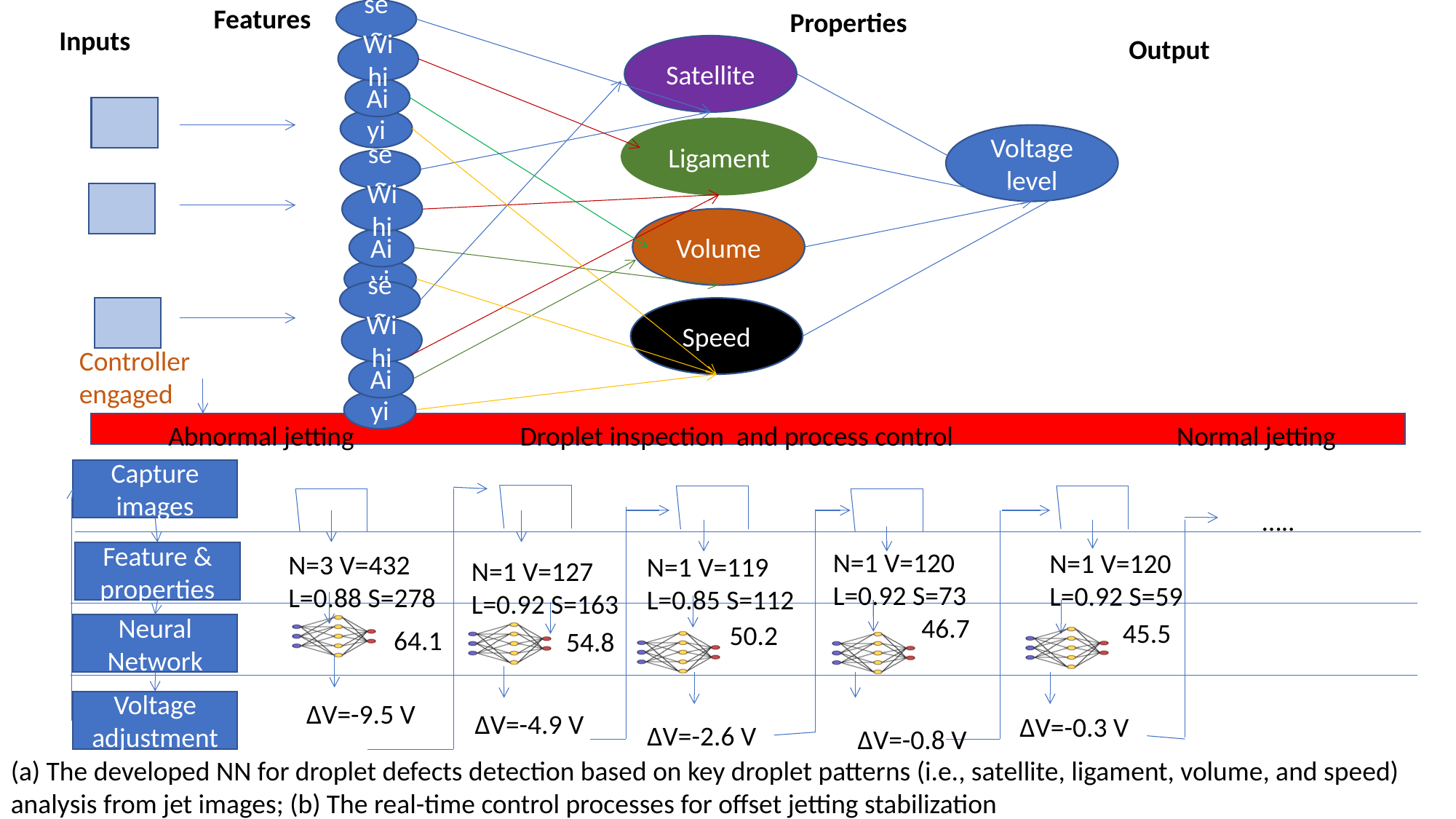

seg
Wi
hi
Ai
yi
Properties
Features
Inputs
Output
Satellite
Ligament
Voltage level
seg
Wi
hi
Ai
yi
Volume
seg
Wi
hi
Ai
yi
Speed
Controller engaged
Abnormal jetting
Droplet inspection and process control
Normal jetting
Capture images
…..
N=1 V=120
L=0.92 S=73
N=1 V=120
L=0.92 S=59
Feature & properties
N=3 V=432
L=0.88 S=278
N=1 V=119
L=0.85 S=112
N=1 V=127
L=0.92 S=163
46.7
45.5
50.2
Neural
Network
64.1
54.8
Voltage adjustment
ΔV=-9.5 V
ΔV=-4.9 V
ΔV=-0.3 V
ΔV=-2.6 V
ΔV=-0.8 V
(a) The developed NN for droplet defects detection based on key droplet patterns (i.e., satellite, ligament, volume, and speed) analysis from jet images; (b) The real-time control processes for offset jetting stabilization

## Slide 49
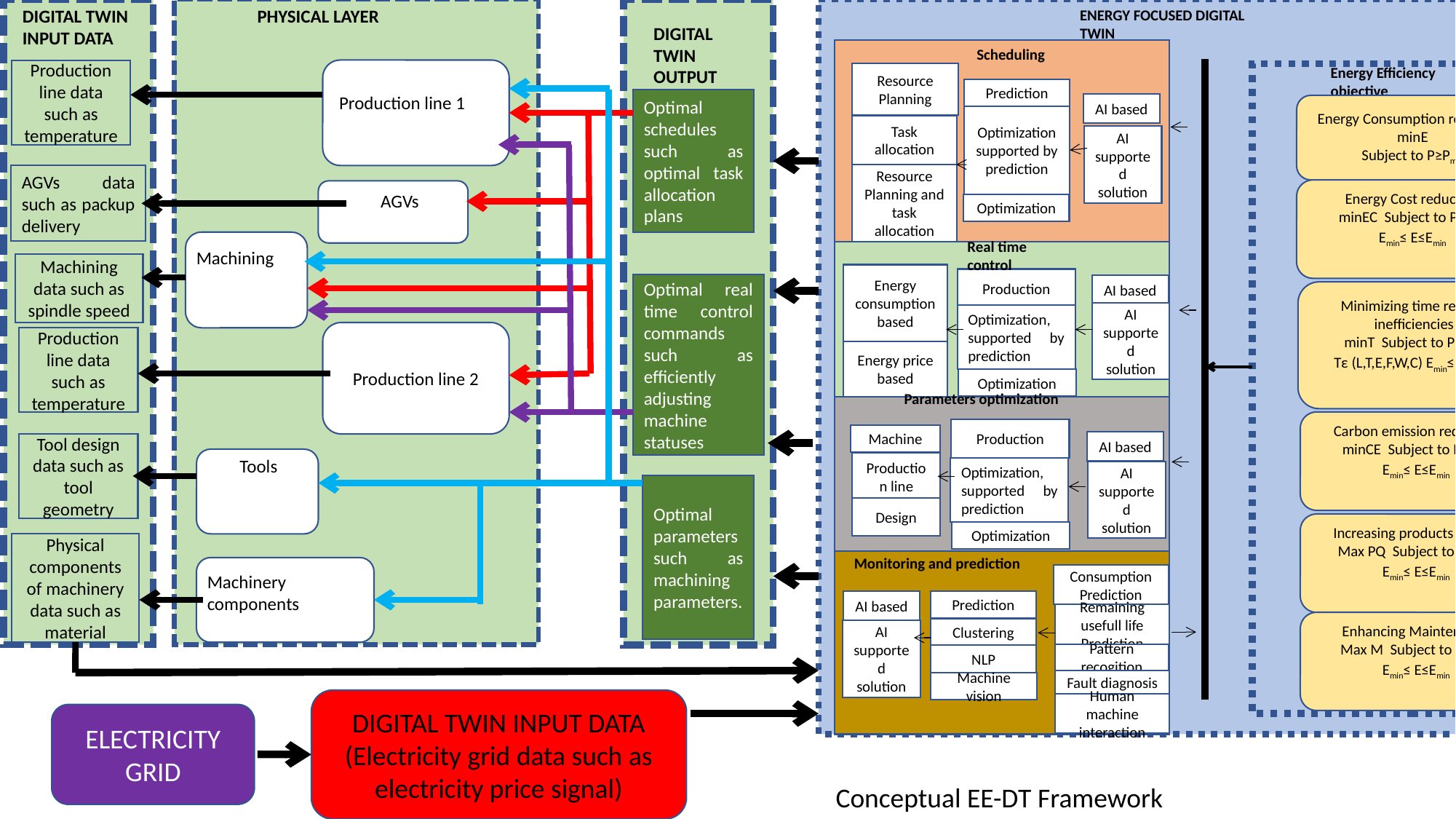

DIGITAL TWIN INPUT DATA
PHYSICAL LAYER
DIGITAL TWIN OUTPUT DATA
Production line data such as temperature
Production line 1
Optimal schedules such as optimal task allocation plans
AGVs data such as packup delivery
AGVs
Machining
Machining data such as spindle speed
Optimal real time control commands such as efficiently adjusting machine statuses
Production line 2
Production line data such as temperature
Tool design data such as tool geometry
Tools
Optimal parameters such as machining parameters.
Physical components of machinery data such as material
Machinery components
ENERGY FOCUSED DIGITAL TWIN
Scheduling
Energy Efficiency objective
Resource Planning
Prediction
AI based
Energy Consumption reduction
minE
Subject to P≥Pmin
Optimization supported by prediction
Task allocation
AI supported solution
Resource Planning and task allocation
Energy Cost reduction
minEC Subject to P≥Pmin
Emin≤ E≤Emin
Optimization
Real time control
Energy consumption based
Production
AI based
Minimizing time related inefficiencies
minT Subject to P≥Pmin
Tε (L,T,E,F,W,C) Emin≤ E≤Emin
AI supported solution
Optimization, supported by prediction
Energy price based
Optimization
Parameters optimization
Carbon emission reduction
minCE Subject to P≥Pmin
Emin≤ E≤Emin
Production
Machine
AI based
Production line
Optimization, supported by prediction
AI supported solution
Design
Increasing products quality
Max PQ Subject to P≥Pmin
Emin≤ E≤Emin
Optimization
Monitoring and prediction
Consumption Prediction
AI based
Prediction
Remaining usefull life Prediction
Enhancing Maintenance
Max M Subject to P≥Pmin
Emin≤ E≤Emin
Clustering
AI supported solution
Pattern recogition
NLP
Fault diagnosis
Machine vision
Human machine interaction
DIGITAL TWIN INPUT DATA
(Electricity grid data such as electricity price signal)
ELECTRICITY GRID
Conceptual EE-DT Framework

## Slide 50
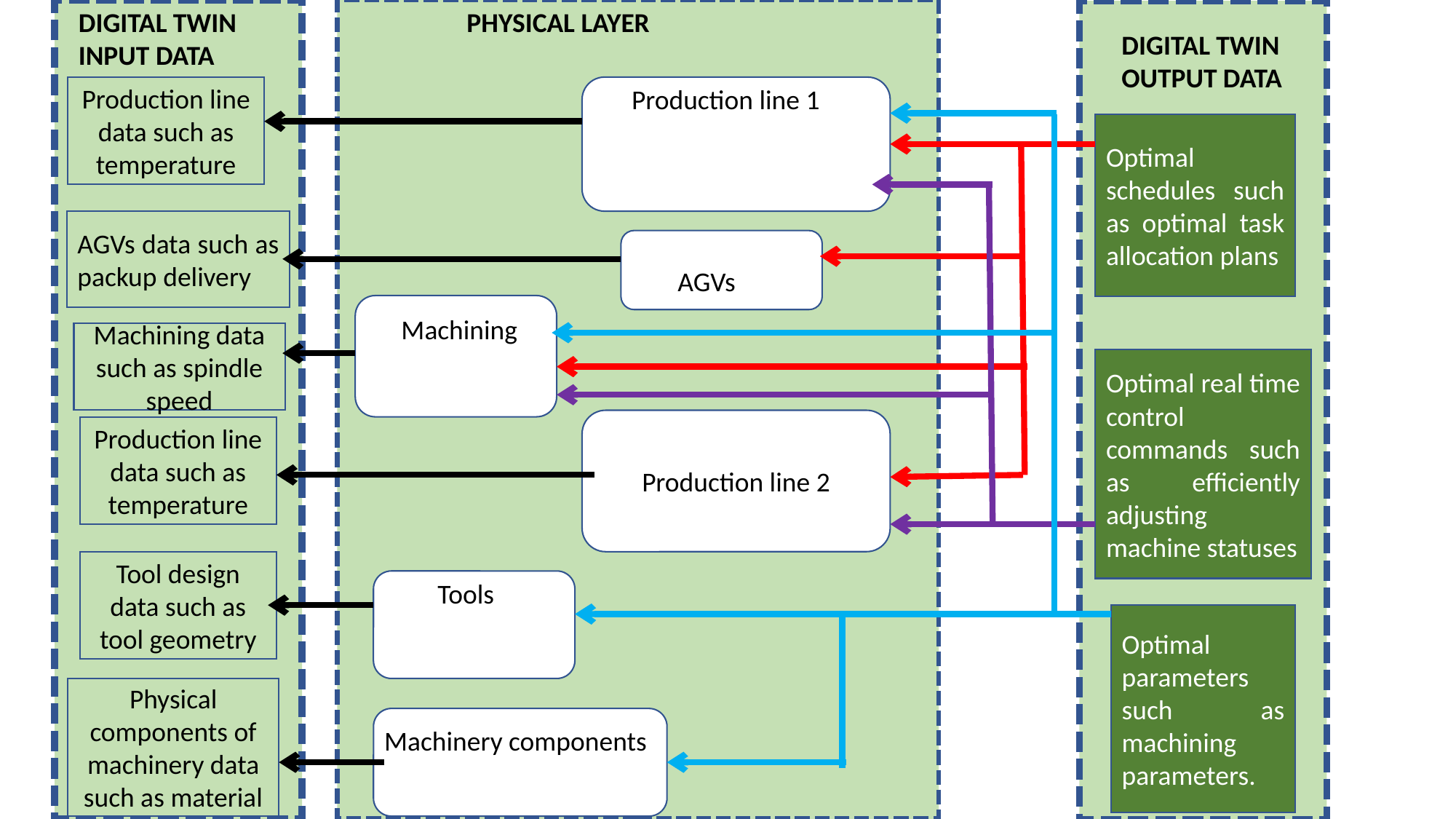

DIGITAL TWIN INPUT DATA
PHYSICAL LAYER
DIGITAL TWIN OUTPUT DATA
Production line data such as temperature
Production line 1
Optimal schedules such as optimal task allocation plans
AGVs data such as packup delivery
AGVs
Machining
Machining data such as spindle speed
Optimal real time control commands such as efficiently adjusting machine statuses
Production line 2
Production line data such as temperature
Tool design data such as tool geometry
Tools
Optimal parameters such as machining parameters.
Physical components of machinery data such as material
Machinery components

## Slide 51
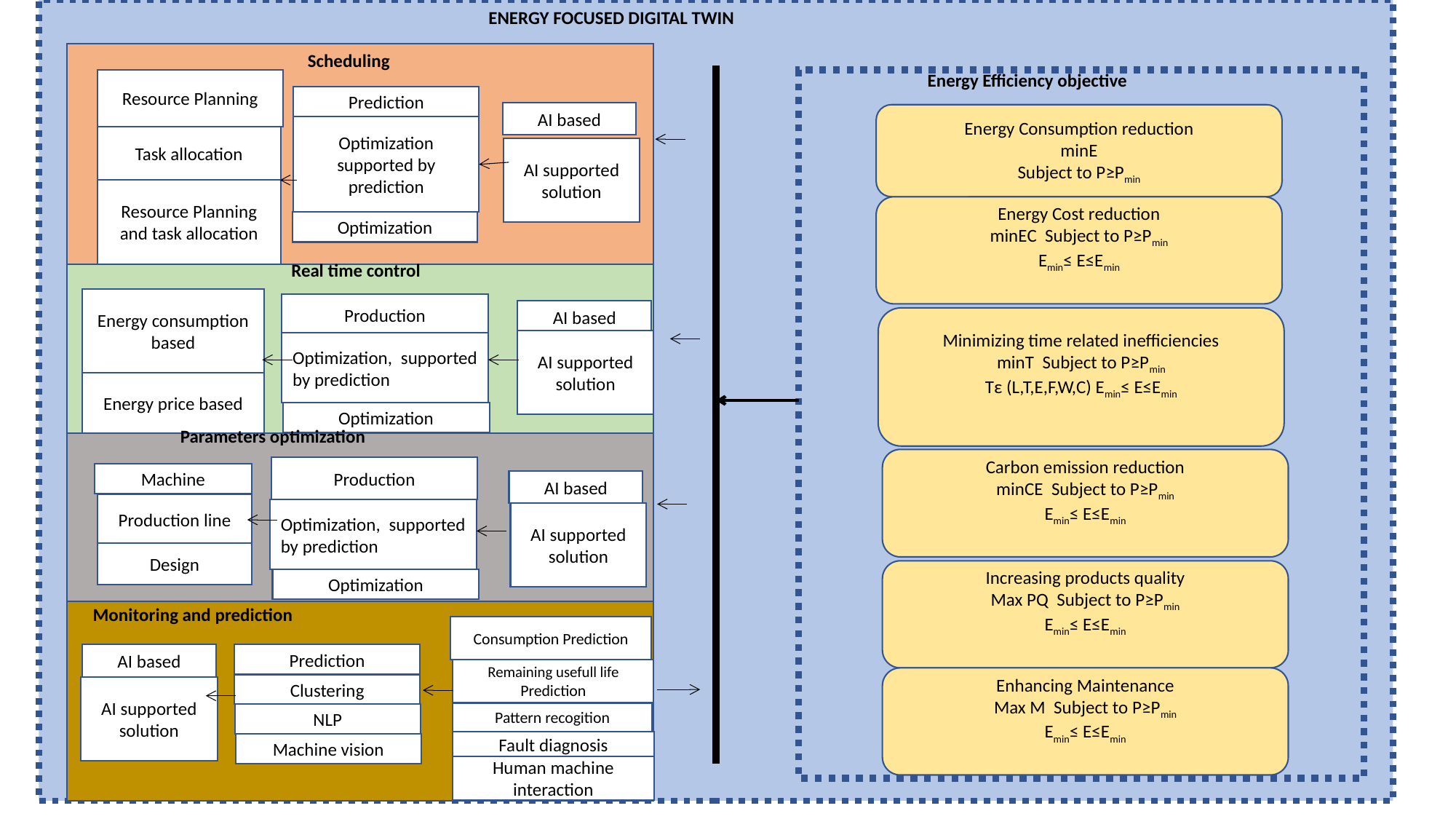

ENERGY FOCUSED DIGITAL TWIN
Scheduling
Energy Efficiency objective
Resource Planning
Prediction
AI based
Energy Consumption reduction
minE
Subject to P≥Pmin
Optimization supported by prediction
Task allocation
AI supported solution
Resource Planning and task allocation
Energy Cost reduction
minEC Subject to P≥Pmin
Emin≤ E≤Emin
Optimization
Real time control
Energy consumption based
Production
AI based
Minimizing time related inefficiencies
minT Subject to P≥Pmin
Tε (L,T,E,F,W,C) Emin≤ E≤Emin
AI supported solution
Optimization, supported by prediction
Energy price based
Optimization
Parameters optimization
Carbon emission reduction
minCE Subject to P≥Pmin
Emin≤ E≤Emin
Production
Machine
AI based
Production line
Optimization, supported by prediction
AI supported solution
Design
Increasing products quality
Max PQ Subject to P≥Pmin
Emin≤ E≤Emin
Optimization
Monitoring and prediction
Consumption Prediction
AI based
Prediction
Remaining usefull life Prediction
Enhancing Maintenance
Max M Subject to P≥Pmin
Emin≤ E≤Emin
Clustering
AI supported solution
Pattern recogition
NLP
Fault diagnosis
Machine vision
Human machine interaction

## Slide 52
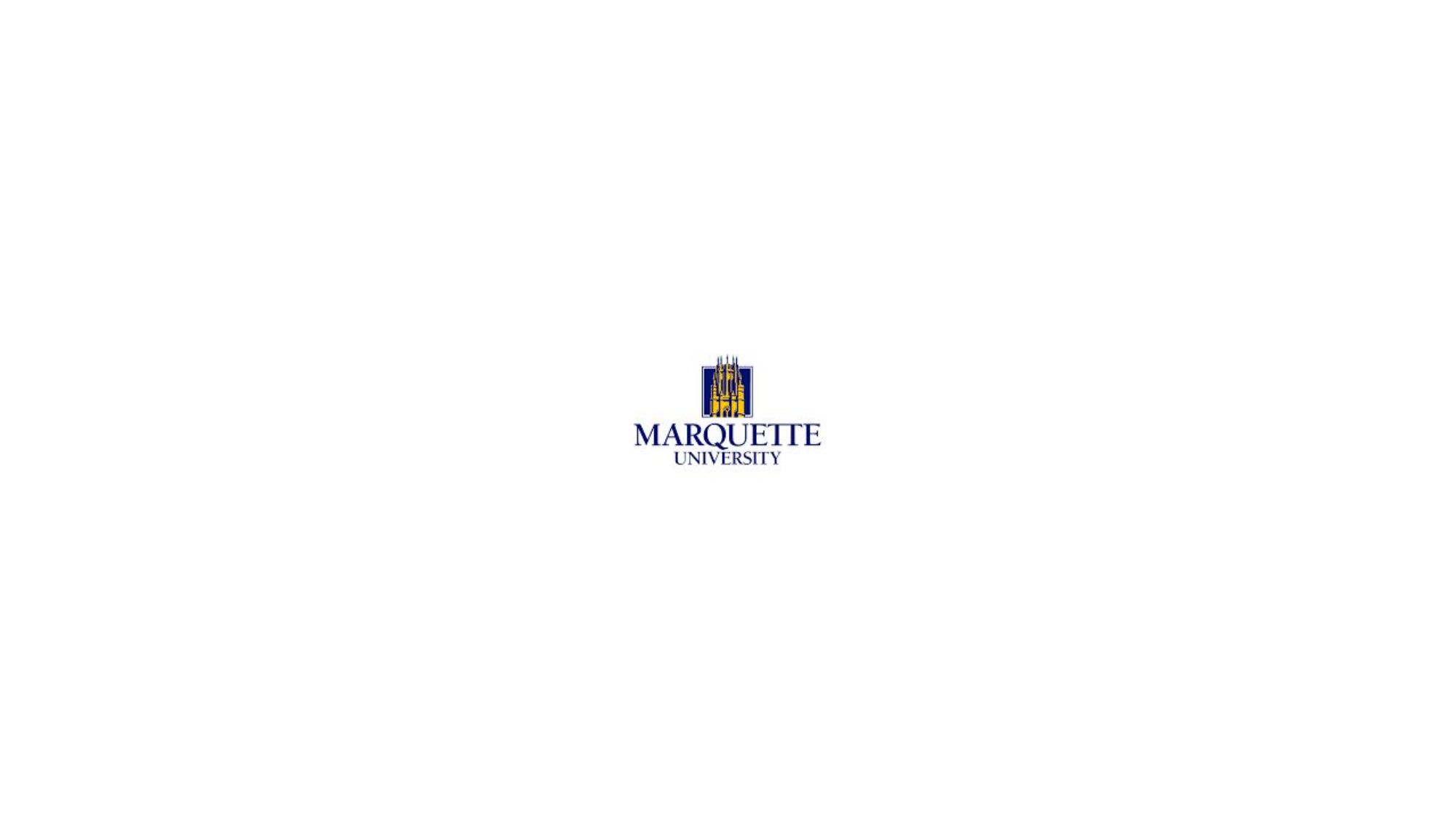

Supplement: Supplementary file 1 [file Supplementaryfile1.pptx]
